# Advances in Noble Metal-Decorated Metal Oxide Nanomaterials for Chemiresistive Gas Sensors: Overview

**DOI:** 10.1007/s40820-023-01047-z

**Published:** 2023-04-07

**Authors:** Li-Yuan Zhu, Lang-Xi Ou, Li-Wen Mao, Xue-Yan Wu, Yi-Ping Liu, Hong-Liang Lu

**Affiliations:** 1 State Key Laboratory of ASIC and System, Shanghai Institute of Intelligent Electronics and Systems, School of Microelectronics, Fudan University, Shanghai, 200433 People’s Republic of China; 2 School of Opto-Electronic Information and Computer Engineering, University of Shanghai for Science and Technology, Shanghai, 200093 People’s Republic of China; 3 State Key Laboratory of Metal Matrix Composites, School of Material Science and Engineering, Shanghai Jiao Tong University, Shanghai, 200240 People’s Republic of China

**Keywords:** Noble metal, Bimetal, Semiconducting metal oxide, Chemiresistive gas sensor, Electronic sensitization, Chemical sensitization

## Abstract

Highly sensitive gas sensors with remarkably low detection limits are attractive for diverse practical application fields including real-time environmental monitoring, exhaled breath diagnosis, and food freshness analysis. Among various chemiresistive sensing materials, noble metal-decorated semiconducting metal oxides (SMOs) have currently aroused extensive attention by virtue of the unique electronic and catalytic properties of noble metals. This review highlights the research progress on the designs and applications of different noble metal-decorated SMOs with diverse nanostructures (e.g., nanoparticles, nanowires, nanorods, nanosheets, nanoflowers, and microspheres) for high-performance gas sensors with higher response, faster response/recovery speed, lower operating temperature, and ultra-low detection limits. The key topics include Pt, Pd, Au, other noble metals (e.g., Ag, Ru, and Rh*.*), and bimetals-decorated SMOs containing ZnO, SnO_2_, WO_3_, other SMOs (e.g., In_2_O_3_, Fe_2_O_3_, and CuO), and heterostructured SMOs. In addition to conventional devices, the innovative applications like photo-assisted room temperature gas sensors and mechanically flexible smart wearable devices are also discussed. Moreover, the relevant mechanisms for the sensing performance improvement caused by noble metal decoration, including the electronic sensitization effect and the chemical sensitization effect, have also been summarized in detail. Finally, major challenges and future perspectives towards noble metal-decorated SMOs-based chemiresistive gas sensors are proposed.

## Highlights

- Recent progress in noble metal-decorated (NM-D) semiconducting metal oxides (SMOs) gas sensors are summarized.
- Gas sensing mechanisms related to noble metal decoration are carefully discussed.
- Crucial challenges facing the development of NM-D SMOs gas sensors are analyzed.

## Graphical abstract

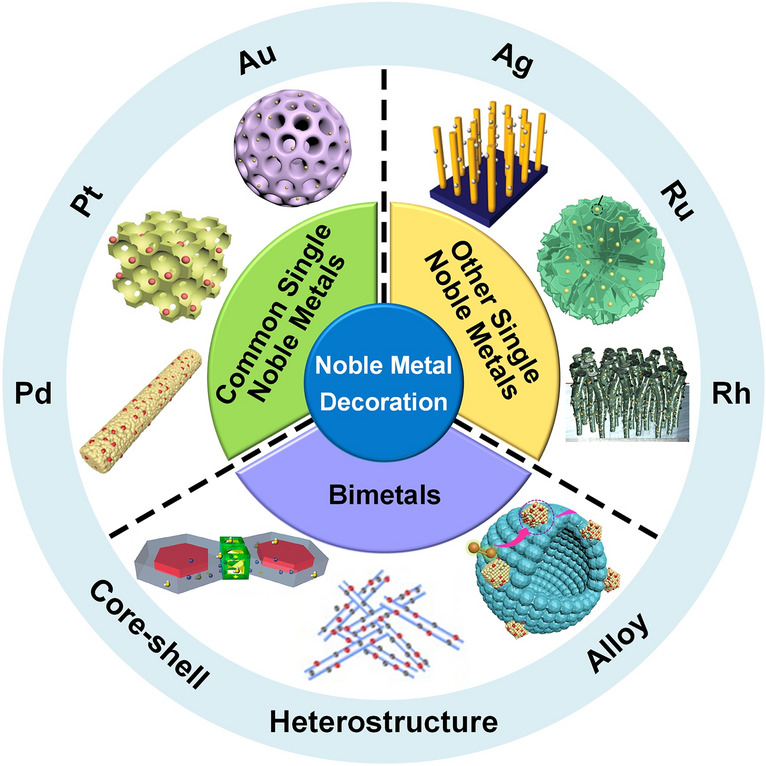

## Introduction

**Fig. 1:**
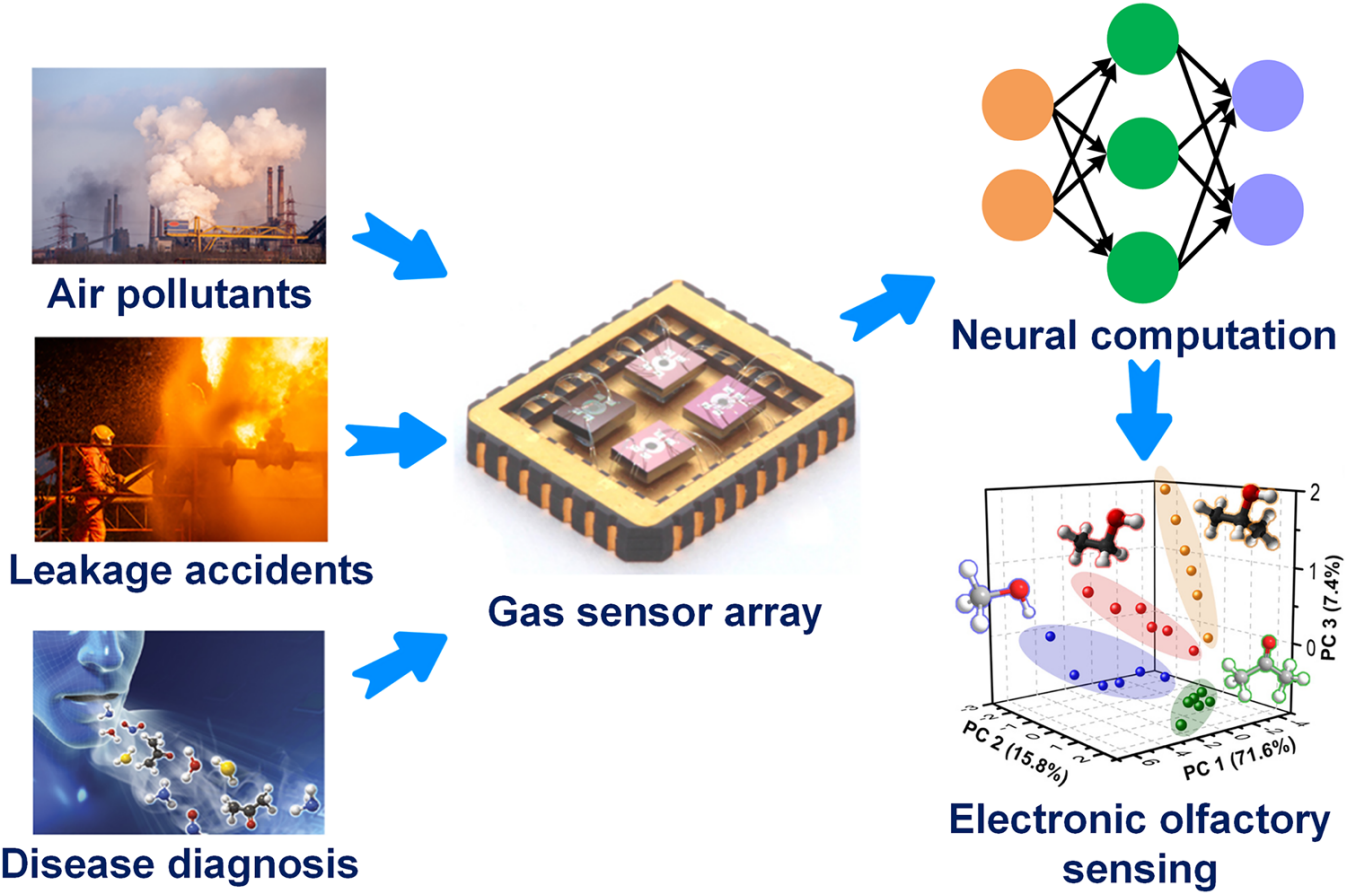
Process of electronic olfactory sensing realization for important applications based on gas sensor arrays. The disease diagnosis figure is reproduced with permission from Ref. [38]. Copyright 2017, Royal Society of Chemistry. The gas sensor array figure is reproduced with permission from Ref. [39]. Copyright 2016, American Chemical Society. The electronic olfactory sensing figure is reproduced with permission from Ref. [40]. Copyright 2021, American Chemical Society

With the rapid development of Internet of Things (IoTs), highly sensitive and selective gas sensors with remarkably low limit of detection (LOD), fast response/recovery speed, and excellent long-term stability and reversibility are in ever increasing demand for smart cities, smart plants, and even smart healthcare [1–4]. On the one hand, ultra-sensitive gas sensors can monitor even trace level hazardous, toxic, or explosive gases like volatile organic compounds (VOCs), hydrogen sulfide (H_2_S), ammonia (NH_3_), formaldehyde (HCHO), nitrogen dioxide (NO_2_), methane (CH_4_), and hydrogen (H_2_), protecting human health from environmental pollutants or leakage accidents [5–7]. On the other hand, highly selective and reliable gas sensors with precise accuracy have shown great application potential in various emerging fields closely related to our daily life, such as food freshness monitoring [8], drunk driving inspecting [9], and non-invasive disease diagnosis through human exhaled breath analysis [10, 11], as shown in Fig. 1. For example, with the advance of modern medicine, endogenous ammonia in exhaled breath have been demonstrated to be important biomarkers for the non-invasive diagnosis of chronic kidney diseases [12].

Among various types of gas sensors including electrochemical [13], optical [14], mass sensitive [15], thermoelectric [16], and magnetic [17] gas sensors, chemiresistive sensors have attracted tremendous research enthusiasm for owning unique advantages of higher sensitivity, smaller size, lower cost, easily manipulated, and even highly integrated for micro-electromechanical systems (MEMS)-based sensors [18, 19]. Generally, chemiresistive gas sensors are mainly comprised of highly sensitive materials, a pair of sensing electrodes, and a pair of heating electrodes providing a high enough operating temperature to active the sensing materials [20]. Since the sensitive material is regarded as the most crucial component of a chemeresistive gas sensor, many efforts have been devoted to develop satisfactory gas sensing materials. Ever since Seiyama invented the first oxide-based gas sensor around the world in 1962 [21], nanostructured semiconducting metal oxides (SMOs) have been considered as prospective gas sensing materials by virtue of their high specific surface area, abundant active adsorption sites, superior electrical properties, and low cost [22–24].

However, single SMO nanomaterial-based gas sensors suffer from low response, poor selectivity, and too high operating temperature, which cannot satisfy the requirements of practical applications. Therefore, diverse methods, mainly consisted of constructing heterostructures [25, 26], decorating catalysts [28], designing charge transfer hybrids [29], and introducing molecular probing and sieving effect [29, 30], have been widely explored for the improvement of gas sensing performance [31]. Among all these strategies, the noble metal decoration, which involves hybrid nanocomposites with synergistic effects and introduces highly active catalysts for gas chemisorption, exactly paves a new extraordinary road for gas sensing performance enhancement and has attracted widespread research attention [27, 32]. Recently, the widely used noble metals mainly include platinum (Pt), palladium (Pd), gold (Au), silver (Ag), ruthenium (Ru), rhodium (Rh), and their bimetal composites. The relevant mechanisms for the gas sensing performance improvement include the electronic sensitization effect of constructing a metal–semiconductor contact [33] and the chemical sensitization effect of the spillover effect [34]. As a result, the synergistic effects not only promote fast interaction between the noble metal decorated SMOs and target gases, but also effectively decrease the operating temperatures by lowering the gas sensing activation energy.

**Fig. 2:**
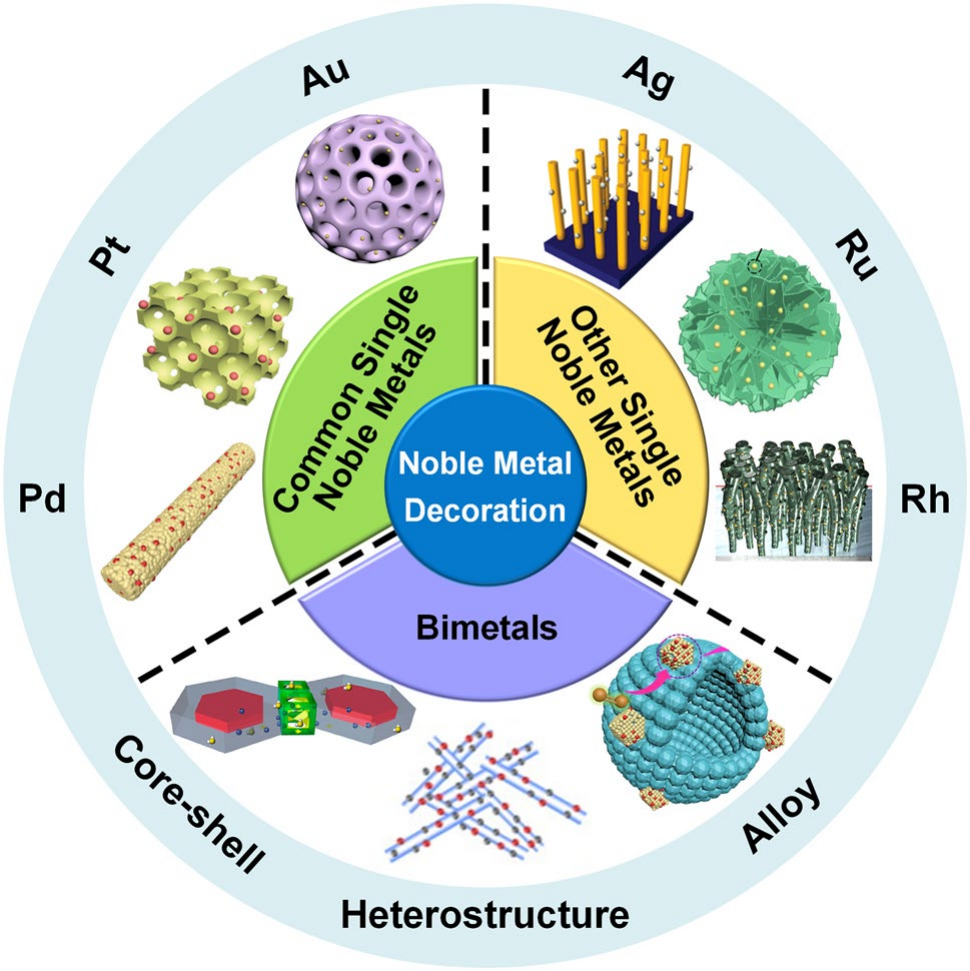
Overview schematic representation of various noble metal decorated SMOs for gas sensors. The Pt NPs figure is reproduced with permission from Ref. [32]. Copyright 2017, Wiley–VCH. The Au NPs figure is reproduced with permission from Ref. [41]. Copyright 2022, Elsevier. The Ag NPs figure is reproduced with permission from Ref. [42]. Copyright 2011, American Chemical Society. The Ru NPs figure is reproduced with permission from Ref. [43]. Copyright 2020, Elsevier. The Rh NPs figure is reproduced with permission from Ref. [44]. Copyright 2019, American Chemical Society. The core–shell NPs figure is reproduced with permission from Ref. [45]. Copyright 2020, American Chemical Society. The heterostructure NPs figure is reproduced with permission from Ref. [46]. Copyright 2013, Elsevier. The alloy NPs figure is reproduced with permission from Ref. [47]. Copyright 2021, Elsevier. The Pd NPs figure is reproduced with permission from Ref. [48]. Copyright 2016, American Chemical Society

Actually, several reviews about noble metal-decorated SMOs-based gas sensors are focused more on either certain type of noble metals, such as Ag-modified SMOs-based gas sensors [35] and Pd-decorated nanostructures-based gas sensors [36], or certain type of SMOs, such as noble metal-decorated nanostructured ZnO-based H_2_ gas sensors [37]. However, few comprehensive reviews focusing on the recent advances in diverse noble metal-decorated SMOs for high-performance gas sensors have been reported. Herein, this review will comprehensively reflect and summarize recent progress in SMOs-based chemiresistive gas sensors decorated by not only common noble metals including Pt, Pd, and Au, but also other unusual noble metals like Ag, Ru, and Rh, and even well-structured bimetals containing alloy structures, core–shell structures, and heterostructures, as shown in Fig. 2. Meanwhile, the different effects of noble metal decoration on different SMOs containing common SMOs of ZnO, SnO_2_, WO_3_, other SMOs (e.g., In_2_O_3_, Fe_2_O_3_, TiO_2_, CuO, NiO, and Co_3_O_4_), and heterostructured SMOs will be comprehensively discussed and summarized as well. In addition to the detailed summarization on conventional gas sensing properties, the innovative applications like photo-assisted room temperature gas sensors and mechanically flexible smart wearable devices are discussed at the same time. Moreover, the relevant mechanisms for the sensing performance improvement caused by noble metal decoration, including the electronic sensitization and chemical sensitization effect will also be summarized in detail. Finally, crucial challenges facing the development of noble metal-decorated SMOs-based gas sensors are identified and feasible routes for effectively improving gas sensing performance like selectivity, power consumption, and long-term stability by constructing integrated sensor arrays, employing neural network algorithms, as well as developing MEMS and field effect transistor (FET)-type devices are carefully considered and proposed. Constructing long-term stable noble metals-decorated SMOs-based gas sensor arrays, combined with advanced neural computation, should allow real-life electronic olfactory sensing in the future (Fig. 1). Overall, the present topical review aims to provide a comprehensive perspective on noble metal-decorated SMOs-based gas sensors, including the material structure, gas sensing mechanisms, properties, applications, challenges, and prospects, hoping to be served as an important reference for newcomers as well as experienced researchers.

## Gas Sensing Mechanisms of Noble Metal-Decorated SMOs

### General Gas Sensing Mechanism of Chemiresistive SMOs

#### Basic Gas Sensing Mechanism of Chemiresistive SMOs

The basic sensing mechanism of chemiresistive SMOs-based gas sensors is based on the oxygen adsorption model [49]. Oxygen molecules in air will preferably accumulate on the surface of SMOs and capture electrons from SMOs, leading to the generation of chemisorbed oxygen species (O_2_^−^, O^−^, or O^2−^) [50] and an electron depletion layer (for n-type SMOs) or a hole accumulation layer (for p-type SMOs). As a result, the resistance of n-SMOs/p-SMOs will then be increased/reduced, respectively. The reaction processes are listed as following:

1 $$ O_{{2\left( {gas} \right)}} { } \to O_{{2\left( {ads} \right)}} $$

2 $$ O_{{2 \left( {ads} \right)}} + e^{ - } \to O_{{2 \left( {ads} \right)}}^{ - } $$

3 $$ O_{{2 \left( {ads} \right) }}^{ - } + e^{ - } \to 2O_{{\left( {ads} \right)}}^{ - } $$

4 $$ O_{{\left( {ads} \right)}}^{ - } + e^{ - } \to O_{{\left( {ads} \right)}}^{2 - } $$

When SMOs are exposed to target gases, the adsorbed gas molecules would react with the chemisorbed oxygen intermediates, releasing the electrons back to SMOs (for reducing gas) or instead extracting more electrons from SMOs (for oxidizing gas), which depends on the redox characteristics of the target gases. Thus, the width of the electron depletion layer/hole accumulation layer and further the resistance of SMOs will be changed accordingly. As a result, the resistance variation indicates the sensitivity of SMOs-based gas sensors.

More specifically, for an n-SMO material, when a reducing gas is introduced, the width of the electron depletion layer will decrease, and thus the resistance of the n-SMO material will be reduced correspondingly. When an oxidizing gas is introduced, the width of the electron depletion layer and therefore the resistance of the n-SMO material will be both increased. For a p-SMO material, when facing a reducing gas, the width of the hole accumulation layer will be reduced due to the recombination of the re-injected electrons and holes, leading to an increase of the resistance. Conversely, when introducing an oxidizing gas, the width of the hole accumulation layer will be further increased because of the further extraction of electrons, thus resulting in a decrease of the p-SMOs resistance.

#### Mechanism of Heterojunction for Improved Gas Sensing Performance

The heterojunction is formed at the contact interface of two different SMO materials, including anisotype heterojunction (i.e., p–n or n–p) and isotype heterojunction (i.e., n–n or p–p) [51]. In order to balance the different Fermi levels at the interface, the charge transfer and depletion/barrier layers formation are introduced, which are positive factors for improving gas sensing performance [51, 52]. Actually, the additional depletion layers at the interface could also participate in the gas sensing behaviors and help increase the resistance variation of the sensing SMOs, leading to the improvement of gas sensing properties.

First considering anisotype heterojunction, take the p–n heterojunction as an example, depletion layers are created by electron–hole recombination on the both p-side and n-side. Thus, a potential conduction barrier is formed and resistance is increased. Exposure of a reducing gas will help consume surface oxygen species and inject electrons into the surface to shrink the depletion layer. It leads to a significant reduction in electrical resistance and achieves a higher gas sensing response.

As for isotype heterojunction, in the n–n heterojunction, for example, an additional electron depletion layer will be formed at the n–n interface causing an increase of the material resistance. After exposed to a reducing gas, the electron depletion layer will be further decreased, resulting in a sharp decrease of resistance and an enhancement of gas sensing response.

### Mechanism of Noble Metal Decoration for Improved Gas Sensing Performance

#### Mechanism of Single Noble Metal Decoration for Enhanced Response

**Fig. 3:**
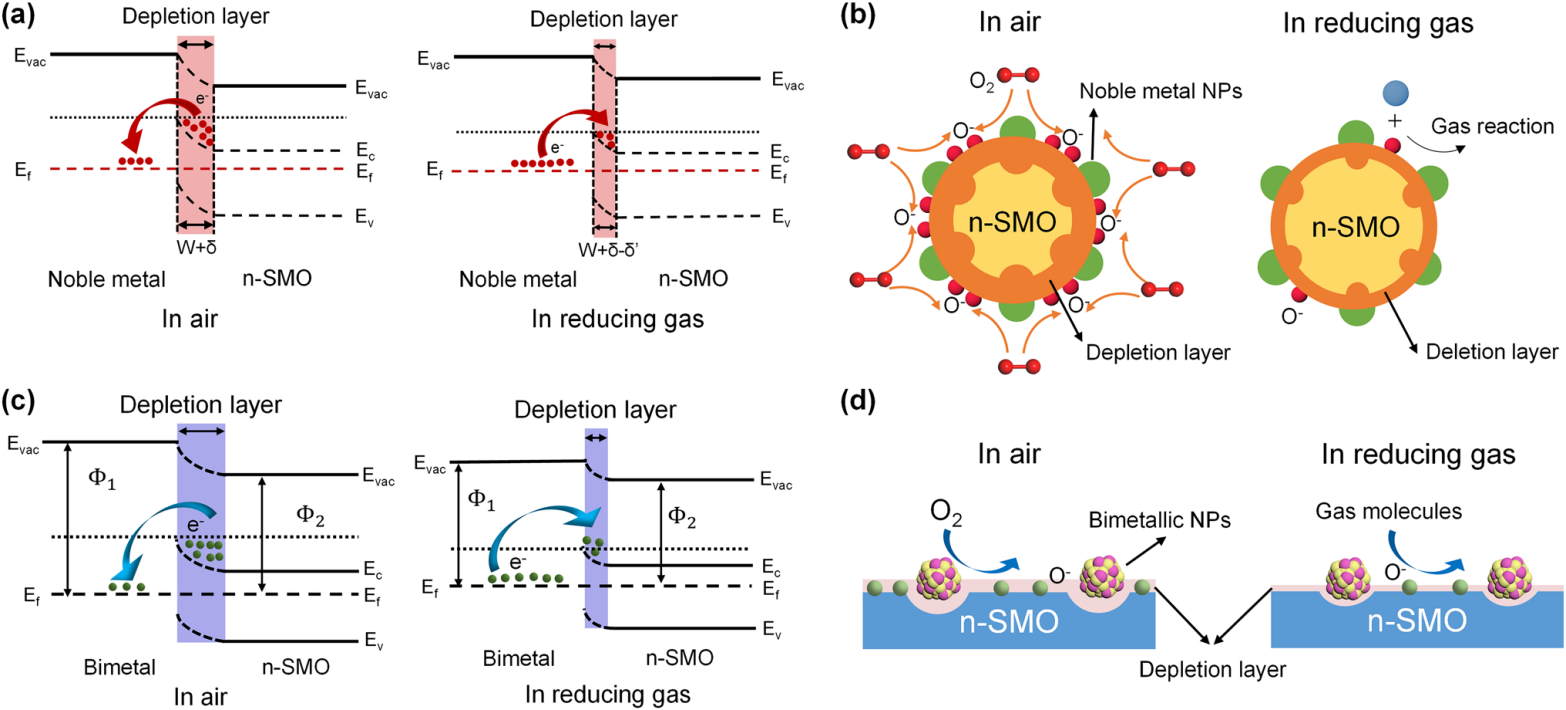
**a** Schematic energy band diagram of noble metal decorated n-SMO. **b** Schematic illustration of the chemical sensitization of noble metal decorated n-SMO. **c** Schematic energy band diagram of bimetal decorated n-SMO. **d** Schematic illustration of the chemical sensitization of bimetal decorated n-SMO

The remarkable improvement of gas sensing performance after noble metal decoration could be attributed to two main mechanisms, the electronic sensitization and the chemical sensitization. First, during the electronic sensitization process, the majority carriers will transfer between noble metals and SMOs due to the mismatch of work functions, leading to the generation of potential barriers. As representatively shown in Fig. 3a, due to the higher work function of noble metals, the electrons generated during the sensing process will be transferred from the conduction band of SMOs into noble metals until the Fermi level is equal [33, 48, 53]. This behavior leads to the formation of a Schottky barrier and an increase in the thickness of the electron depletion layer, which could inhibit the recombination of separated electron–hole pairs and cause a significant change of the resistance when exposure to target gases, resulting in a much higher response. Second, during the chemical sensitization process, the noble metals could facilitate the dissociation of oxygen molecules to produce more reactive chemisorbed oxygen ions which then spill over the surface of SMO to react with more target gas molecules [54–56]. Thus, the chemical sensitization is also known as the spillover effect. As exhibited in Fig. 3b, oxygen molecules will preferentially adsorb on the noble metal nanoparticles (NPs) and then be dissociated into O^−^ which then spill over to the surface of SMOs. Taking the reducing gas as an example, the large amount of O^−^ could react with more target reducing gas molecules and cause the release of electrons, resulting in the rapid resistance change and thus the significant improvement of the gas sensing performance. Besides, the decoration of noble metal NPs indeed increases the specific surface area of the sensing materials, providing more catalytic active sites for gas diffusion and adsorption [57].

#### Mechanism of Bimetal Decoration for Enhanced Response

The mechanisms of bimetallic decoration on SMOs for the gas sensing performance enhancement are also discussed here. Figure 3c–d exhibit the representative electronic sensitization and the chemical sensitization processes of bimetal-decorated SMOs [58–60], respectively, which are similar to monometallic decoration discussed before. More specifically, Fig. 3c exhibits the established Schottky barrier and the additional depletion layer caused by the much higher work function of bimetals than SMOs. Accordingly, the additional depletion layer promotes the transfer of electrons during the gas sensing process, thus improving the gas sensing properties of the bimetal-decorated SMOs [57]. In addition, as shown in Fig. 3d, the bimetals could also catalyze the dissociation of oxygen molecules and lead to the spill-over of reactive chemisorbed oxygen ions on the surface of SMO, facilitating the reaction between target gases and reactive oxygen ions and therefore enhancing the gas sensing performance [58]. Apart from the simple combination of two monometallic properties, the synergistic effects further improve the physical and chemical properties as well as promote the gas sensing performance [61]. Specifically, bimetal composites could introduce tunable electronic structure, morphology, and stoichiometry, thus providing designable energy band structures and catalytic characteristics [62]. Moreover, bimetallic decoration could further lower the activation energy of the sensing reaction via synergistic catalysis, which contributes to a decrease of the operating temperature and an acceleration of the response/recovery process [63, 64].

#### Mechanism of Noble Metal Decoration for Improved Selectivity

Based on various reported literatures, different noble metal decoration tends to promote the gas sensing performance towards different certain gases due to the special catalytic sensitization. Herein, some of the most important affecting mechanisms related to the catalytic sensitization are discussed. First, the selective reaction occurred between a specific noble metal and a certain gas greatly attributes to the enhanced selectivity. Most obviously, the Pd-decorated SMOs have a specific enhancement for H_2_ gas molecules, which could be ascribed to the reversible conversion of Pd to PdH_x_ [65, 66]. Pd possesses the unique ability to adsorb and dissociate H_2_ molecules into H atoms. Particularly, by combing with dissociated H atoms, Pd can be converted into PdH_x_ according to the reaction of 2Pd + xH_2_ $\leftrightarrow$ 2PdH_x_ [67]. Subsequently, due to the reduced work function of PdH_x_ compared with Pd, more electrons will be injected into SMOs promoted by the lower Schottky barrier, leading to a further decrease of the material resistance [68]. Therefore, Pd is considered as the best emerging noble metal for the decoration of SMOs-based H_2_ sensors.

**Fig. 4:**
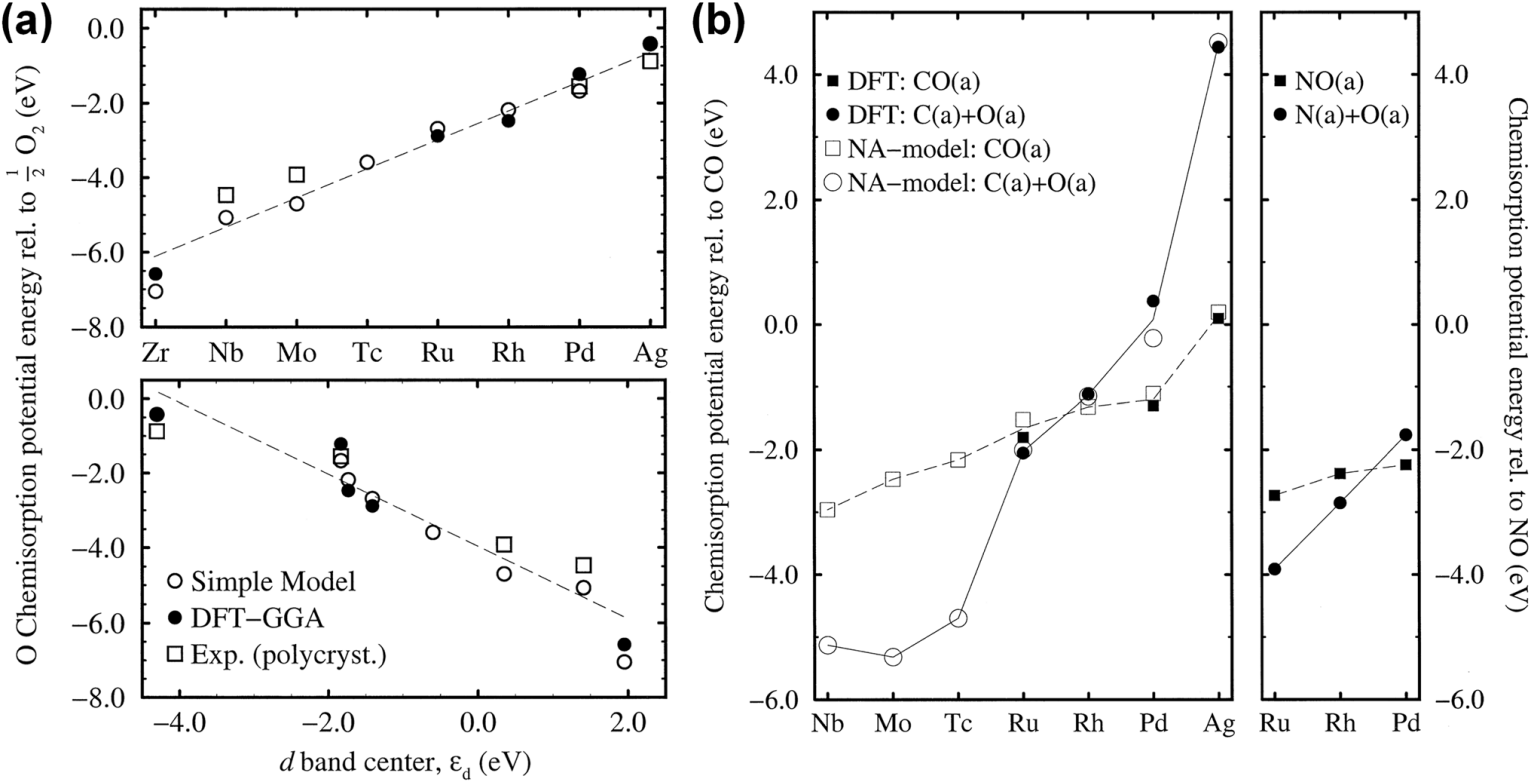
**a** Calculated, model estimates, and experimental results of the variation in the oxygen adsorption energy of a series of 4*d* transition metals, which exhibited a good correlation with *ε*_*d*_. **b** Calculated and model estimates of the variation in the adsorption energy of a series of 4*d* transition metals to CO and NO. Reproduced with permission from Ref. [69]. Copyright 2000, Elsevier

Second, the strength of the coupling effects between specific noble metals and certain gas molecules will greatly influence the improvement of selective detection. The strong electronic effect of gas molecules will form a coupling effect with specific noble metals and therefore significantly affect the molecule adsorption behaviors on metals, which will even surpass the steric effect of certain molecules. More specifically, the coupling effect originates from the interactions between the adsorbate valence states and the *s*- and *d*-states of a certain metal [69]. Since the influence of the coupling to the metal *s*-states is approximately the same for different noble metals, the strength of the coupling effect largely depends on the coupling to the *d* electrons. Moreover, since the *d* bands are recognized to be narrow, the coupling of molecules to the *d* electrons will cause a splitting of the molecule resonance and generate both bonding state and antibonding state above the *d* bands, leading to the strong interactions [69]. Therefore, the *d*-band model could effectively reflect the trends in the molecule coupling strengths on noble metal surfaces, which will help to guide the selection of noble metals for highly selective target gases detection. Meanwhile, the band center (*ε*_*d*_) could well describe the characteristics of the *d* bands, which is commonly chosen as the representative parameter. For instance, as shown in Fig. 4a, both the theoretical calculations and experimental results reveal that, as *ε*_*d*_ relative to the Fermi level becomes more positive, the oxygen adsorption energy gets larger [69], which will greatly affect the gas sensing response. Similar theoretical calculation results were observed in the adsorption energies of a series of 4*d* transition metals to CO and NO exhibited in Fig. 4b.

Last but not least, since the adsorption of gas molecules on the noble metal surface is the primary condition for any possible catalytic reactions, the selective adsorption of gas molecules on a certain noble metal surface under different temperature will also affect the selectivity of noble metal decorated SMOs-based sensors. For example, Kim et al. [70] reported that Au decorated SnO_2_ NWs exhibited special enhancement of selectivity to carbon monoxide (CO) rather than benzene (C_6_H_6_) and toluene (C_7_H_8_) at 300 °C, which can be attributed to the desorption of all C_6_H_6_ molecules and their dissociated biphenyl groups from Au surfaces above 127 °C observed by the previous experimental result [71].

### Recent Advanced Strategies for Further Understanding on the Gas Sensing Mechanism of Noble Metal-Decorated SMOs

#### Density Functional Theory Calculations

Recently, the first-principle calculations based on the density functional theory (DFT) have been widely employed for further exploration on the adsorption properties of various noble metal-decorated SMOs-based gas sensing materials [72–74]. Specifically, different parameters including the adsorption energy, adsorption distance, and charge transfer could all reflect the adsorption properties.

First, the adsorption energy (*E*_*ads*_) is defined as *E*_*ads*_ = *E*_*sub-gas*_–(*E*_*sub*_ + *E*_*gas*_), where *E*_*sub-gas*_ represents the total energy of the gas molecule–substrate adsorption system, and *E*_*sub*_/*E*_*gas*_ denotes the energy of the substrate and the isolated gas molecule, respectively. Accordingly, *E*_*ads*_ < 0 implies that the adsorption process is exothermic and spontaneous, leading to an energetically stable adsorption system [19]. Meanwhile, the more negative adsorption energy indicates a much stronger adsorption reaction as well as a higher sensing response. For example, Liu et al. [75] constructed geometrically optimized Pd_4_ cluster-decorated SnO_2_ and intrinsic SnO_2_ models for H_2_ and acetylene (C_2_H_2_) adsorption and calculated the corresponding adsorption energies via the DFT method. As a result, the adsorption energies of H_2_/SnO_2_, H_2_/Pd_4_-SnO_2_, C_2_H_2_/SnO_2_, and C_2_H_2_/Pd_4_-SnO_2_ are − 0.205, − 0.574, − 0.974, and − 1.282 eV, respectively, indicating a better gas sensing performance of Pd decorated SnO_2_ than intrinsic SnO_2_. Their follow-up experimental measurements further demonstrate much higher responses of Pd decorated SnO_2_ than intrinsic SnO_2_ towards H_2_ and C_2_H_2_, which corresponds well with the theoretical calculation results.

Second, since the chemical adsorption takes place when the bonds between the target gas molecules and the exposed atoms on the substrate materials are formed, the specific adsorption distance therefore can help reveal the gas adsorption behaviors [76]. More specifically, the shorter the adsorption distance, the stronger the adsorption interaction. For instance, for the comparison of the gas adsorption performance between intrinsic ZnO and Pd decorated-ZnO, Liangruksa et al. [77] investigated the adsorption distances between the H_2_ molecule and the nearest atoms of the substrates after adsorption. In detail, the adsorption distances for *d*_O(ZnO)−H(gas)_ and *d*_Pd(Pd2 cluster)−H(gas)_ are calculated to be 3.70 and 1.58 Å, respectively, implying the enhanced H_2_ gas adsorption interaction after Pd decoration on the ZnO surface. In addition, the H–H bond length of the H_2_ molecule stretches longer after the chemical adsorption, which benefits the dissociation of the H–H bond and in turn promotes the H_2_ adsorption reaction.

Third, the charge transfer (∆Q) refers to the quantity of electons transferred from gas molecules to substrate materials. Consequently, the greater value of ∆Q means the more electrons transferred, further indicating the higher gas sensing response [78]. For example, based on Barder charge analysis, Chen et al. [79] discovered that the charge transfer for the C_6_H_6_ molecule adsorbed on intrinsic, Pd-decorated, and Pt-decorated ZnO monolayers are calculated to be approximately 0.01798*e*, 0.0231*e*, and 0.0243*e*, respectively. Accordingly, the noble metal (Pd, Pt) decoration could enhance the charge transfer as well as the gas sensing performance.

#### In-situ Transmission Electron Microscopy Analysis

With the rapid progress of modern science and technology, increasingly advanced characterization techniques have been applied in the field of gas sensing to further reveal the morphology and composition changes before and after the gas sensing process. Significantly, the emerging *in-situ* transmission electron microscopy (TEM) technique can help to directly observe the solid–gas reaction process and record the morphological and compositional evolution of the sensing materials during the real-time gas sensing process at the desired temperature [80, 81], which is urgently deserved to be used in gas sensing mechanism investigation. For example, Wang et al. [82] revealed the failure mechanisms of bimetal Pd–Ag nanoparticles in ZnO-based H_2_ sensors under operation conditions with the help of gas-cell *in-situ* TEM, which further guided the optimized design of satisfactory H_2_ sensors with excellent long-term stability. In detail, the morphological evolution of bimetal Pd–Ag nanoparticles on the surface of ZnO nanowires at two characteristic operating temperatures of 300 and 500 °C was observed in real time on the *in-situ* TEM characterization platform. Based on the *in-situ* TEM analysis, two reasonable failure mechanisms of the bimetal Pd–Ag nanoparticles are proposed: particles coalescence at 300 °C and phase segregation at 500 °C. However, particles coalescence will lead to the formation of large nanoparticles with degraded catalytic activities, and phase segregation of Ag migrating from the Pd–Ag alloy nanoparticles will result in the decrease of the synergistic effect, both causing the degradation of gas sensing performance. In addition, Hui et al. [83] synthesized Au/WO_2.7_ nanocomposites and carefully investigated the changes in morphology and electronic structure under H_2_ atmosphere by *in-situ* TEM, which helped to analyze the intrinsic interaction between Au/WO_2.7_ nanocomposites and H_2_. As a result, both swing and sintering processes of Au NPs were observed at the heating environment, which could indicate the inner mechanism for the change rule of sensing performance with increasing operating temperature. Meanwhile, no injection of H atoms was observed in the surface of Au/WO_2.7_ nanocomposites after the introduction of H_2_ into the vacuum environment, which suggested that the H_2_ sensing mechanism for Au/WO_2.7_ nanocomposites under a vacuum are dominated by mass transport pathways.

## Single Noble Metal-Decorated SMOs-Based Gas Sensors

### Pt-Decorated SMOs-Based Gas Sensors

#### Pt-decorated ZnO Gas Sensors

**Fig. 5:**
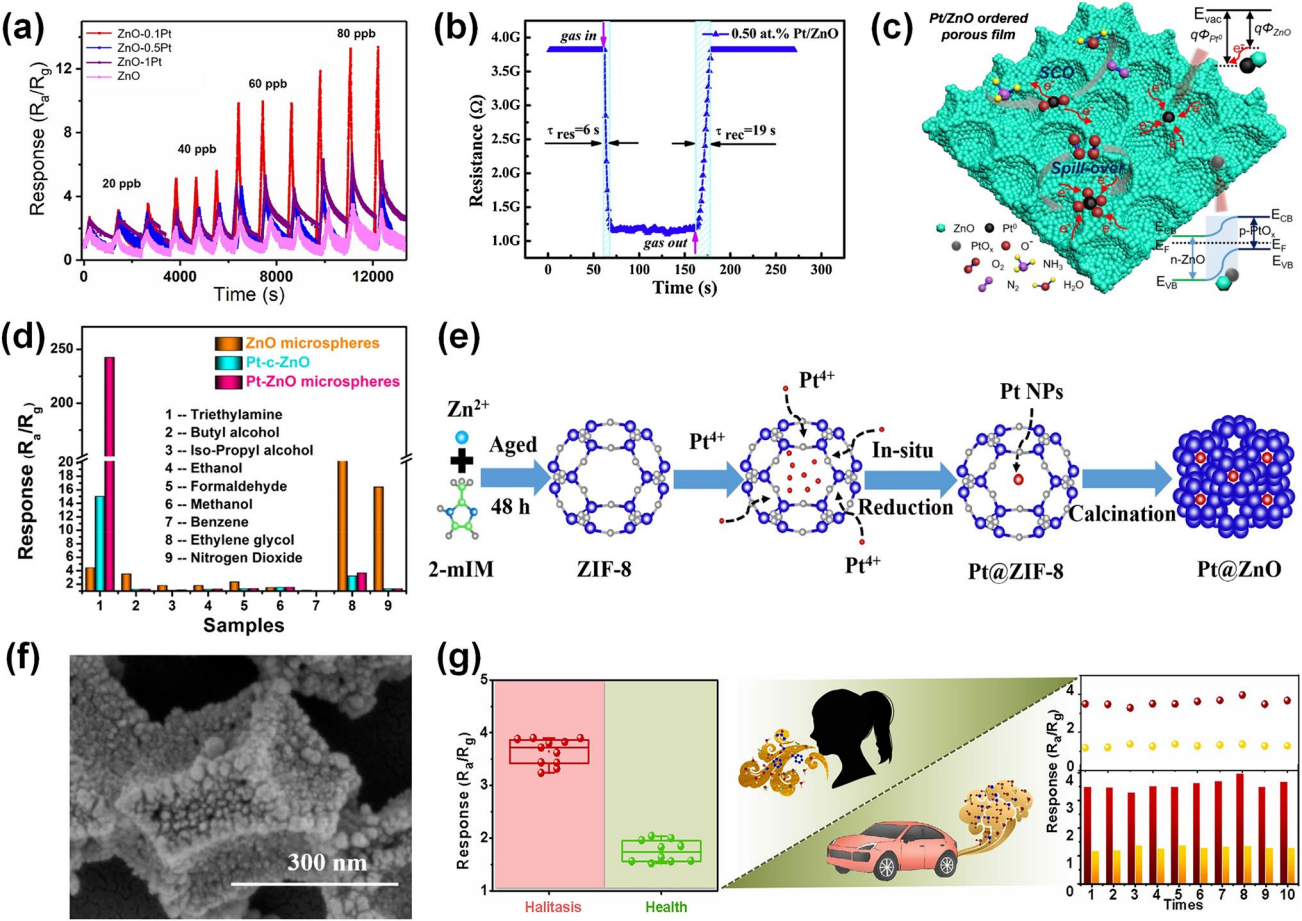
**a** Response of the pristine and Pt-decorated ZnO sensors towards different concentrations varying from 20 to 80 ppb. Reproduced with permission from Ref. [89]. Copyright 2021, Elsevier. **b** Response and recovery behavior of 0.50 at% Pt-decorated ZnO to 50 ppm CO at 180 °C. Reproduced with permission from Ref. [34]. Copyright 2020, Elsevier. **c** Mechanism of Pt-decorated ZnO sensor toward NH_3_. Reproduced with permission from Ref. [90]. Copyright 2020, Elsevier. **d** Responses of pure ZnO, Pt-commercial ZnO, and Pt-decorated ZnO microspheres-based sensors to various gases. Reproduced with permission from Ref. [72]. Copyright 2021, Elsevier. **e** Fabrication process of Pt@ZnO polyhedrons. **f** SEM image of Pt@ZnO polyhedrons. Reproduced with permission from Ref. [91]. Copyright 2021, Elsevier. **g** Responses of 3DIO ZnO and 3DIO Pt-decorated ZnO@ZIF-8 based sensors to simulated H_2_S abnormal/healthy breath samples. Reproduced with permission from Ref. [92]. Copyright 2022, Elsevier

Pt-decorated ZnO materials were synthesized with various morphologies including zero-dimensional (0D) nanoparticles [84], one-dimensional (1D) nanorods [85], nanotubes [86], two-dimensional (2D) nanosheets [87], and three-dimensional (3D) hierarchical structures [88]. Benefiting from high specific surface area and continuous electron transfer pathways, Pt-decorated 1D ZnO nanostructures exhibit enhanced sensing behaviors such as reduced working temperature, remarkable sensitivity and selectivity, and fast response/recovery process. For instance, Yu et al. [89] reported a Pt-decorated ZnO nanorods sensor through a facile immersion-calcination method, which was highly sensitive to target ppb-level H_2_S gas. As shown in Fig. 5a, the Pt-decorated ZnO-based sensor exhibited the highest response at each concentration (20–80 ppb) than that of other sensors. Owing to the excessive enhanced surface catalytic activity, when the concentration of the Pt NPs was increased, the response of the sensor was degraded. Moreover, the response of the Pt-decorated ZnO-based sensor was 23.1 towards at 260 °C at a low H_2_S concentration of 100 ppb, which was 5.8-fold higher than the pure ZnO sensor (3.4). Besides, the lowest H_2_S detection of the Pt-ZnO-based sensor could obtain as low as 1.1 ppb. In brief, the excellent H_2_S sensor can be achieved by combining the sensitization effect of Pt and the specific surface area of nanorods. Young et al. [85] prepared Pt NPs-decorated ZnO nanorods via a hydrothermal strategy. Due to the spillover effect of Pt, the response of Pt-decorated ZnO nanorods-based sensor to 1000 ppm methanol was significantly improved from 1.34 to 121.03 at 270 °C (Table 1).

**Table 1:** Summary of the reported Pt-decorated SMOs-based gas sensors

Materials	Structure	Synthesis method	Target gas	O. T. (°C)	Conc. (ppm)	Response	t_res_/t_re_	LOD	Refs.
ZnO	Nanorods	Hydrothermal	H_2_S	260	0.3	65^a^	40/430 s	1.1 ppb	[89]
ZnO	Nanorods	Hydrothermal	Methanol	270	1000	121.03^a^	–	–	[85]
ZnO	Nanosheets	Hydrothermal	CO	180	50	3.57^a^	6/19 s	100 ppb	[34]
ZnO	Ordered porous thin film	PS template	NH_3_	330	100	324^a^	102/5731 s	–	[90]
ZnO	Microspheres	Hydrothermal	TEA	200	100	242^a^	15/70 s	–	[72]
ZnO	Polyhedrons	Pyrolysis of ZIF-8	CO	300	0.5	9%^b^	1.5/14 s	100 ppb	[91]
ZnO	3DIO	PMMA template	H_2_S	310	5.5	118^a^	136/200 s	40 ppb	[92]
SnO_2_	Microbelts	Electrospinning	Acetone	350	2	93.56^a^	9.2 s/–	–	[55]
SnO_2_	Fibers	Electrospinning	Acetone	300	3	3.47^a^	11/6 s	120 ppb	[95]
SnO_2_	Porous nanotubes	Electrospinning	Acetone	350	5	192^a^	–	10 ppb	[96]
SnO_2_	Nanoneedles	Hydrothermal	CO	250	100	23.18^a^	15/14 s	–	[98]
SnO_2_	Nanorods	Hydrothermal	H_2_	RT	1000	87.35%^b^	0.33/29.6 s	–	[99]
SnO_2_	Mesoporous nanoflowers	Double-template technique	H_2_S	30	1	68^a^	11/75 s	100 ppb	[101]
SnO_2_	Sheets	Solution combustion	Isopropanol	220	100	190.5^a^	–	–	[56]
SnO_2_	Ultrathin film	ALD	TEA	200	10	136.2^a^	3/6 s	7 ppb	[104]
WO_3_	Nanorods	GLAD	H_2_	200	3000	2.2 × 10^5a^	~ 5.6/ ~ 15 min	–	[53]
WO_3_	Nanorods	Evaporation	H_2_	110	5000	68%^b^	0.64/6.29 s	10 ppm	[110]
WO_3_	Nanorods	Hydrothermal	H_2_S	200	10	1638.2^a^	42/37 s	5 ppb	[112]
WO_3_	Porous belts	Electrospinning	H_2_S	365	5	378.12^a^	6.1/288.2 s	400 ppb	[113]
WO_3_	Microporous nanofibers	Electrospinning	H_2_S	350	5	834.2^a^	–	100 ppb	[114]
WO_3_	Ordered mesoporous	Evaporation induced co-assembly	CO	125	100	10.1^a^	16/1 s	–	[32]
WO_3_	Films	GLAD	NO_2_	150	1	11.24^a^	27/34 s	80 ppb	[115]
WO_3_	Nanoflakes	Hydrothermal	Acetone	250	3.8	290%^b^	–	237 ppb	[54]
WO_3_	Square-like	Hydrothermal	NH_3_	270	1000	100.09^a^	8/11 s	1 ppm	[116]
In_2_O_3_	Mesoporous nanofibers	Electrospinning	NO_2_	RT	1	23.9^a^	–/358 s	10 ppb	[117]
In_2_O_3_	Nanorods	Vapor–liquid–solid growth	NO_2_	300	200	11^a^	45/60 s	–	[119]
In_2_O_3_	Mesoporous nanofibers	Electrospinning	Acetone	180	1	15.1^a^	6/9 s	10 ppb	[120]
In_2_O_3_	Nanowires	Electrospinning	Acetone	320	1	6.23^a^	11/13 s	10 ppb	[121]
NiO	Nanotubes	Electrospinning	Ethanol	200	100	20.85^a^	–	–	[122]
NiO	Core–shell structure	Sol–gel	H_2_	RT	5000	4.25^a^	91/8 s	–	[123]
NiO	Thin films	RF sputtering	NH_3_	300	1000	1278%^b^	15/76 s	10 ppb	[124]
NiO	Porous nanosheets	Hydrothermal	HCHO	300	100	7.16^a^	–	10 ppm	[125]
Fe_2_O_3_	Nanocubes	Hydrothermal	Acetone	139	100	25.7^a^	3/22 s	–	[126]
Fe_2_O_3_	Nanowires	Electrospinning	H_2_S	175	10	157^a^	–	–	[127]
CuO	Porous structure	Calcination	HCHO	225	1.5	5.46^a^	–	100 ppb	[128]
SnO_2_/ZnO	Core–shell nanowires	ALD	Toluene	300	0.1	279^a^	–	–	[130]
SnO_2_/ZnO	Core–shell nanosheets	ALD	H_2_S	375	5	30.43^a^	67/116 s	1 ppm	[131]
SnO_2_/α-Fe_2_O_3_	Hollow nanospheres	*In-situ* reduction	Styrene	206	10	10.56^a^	3/15 s	50 ppb	[132]
In_2_O_3_/WO_3_	Nano powder	Impregnation	NO	RT	0.5	330^a^	10.5/132 min	25 ppb	[133]
ZnO/In_2_O_3_	Nanofibers	Electrospinning	Acetone	300	100	57.1^a^	1/44 s	500 ppb	[134]

*O. T.* operating temperature; *Conc*. concentration; *t*_*res*_*/t*_*rec*_ response time/recovery time; *LOD* limit of detection

^a^Response is defined as R_a_/R_g_ or R_g_/R_a_, R_a_: resistance of the sensor in air, R_g_: resistance of the sensor exposed to target gas

^b^Response is defined as ∆R/R_a_ $\times$ 100% or ∆R/R_g_ $\times$ 100%, ∆R: the change in resistance, which equals to |R_a_–R_g_|

Pt-decorated 2D ZnO structures have attracted much attention owing to their controllable morphology, exhibiting diverse sensing properties [90]. Wang et al. [34] fabricated the Pt-decorated ZnO nanosheets with various Pt concentration through a simple wet-chemical method. The 0.50 at% Pt-decorated ZnO displayed the most excellent sensing properties among 0.25, 0.75, and 1.00 at% Pt/ZnO, demonstrating that a reasonable Pt concentration was crucial to promote the gas sensing behavior. Specially, the working temperature of Pt-decorated ZnO senor was decreased from 210 to 180 °C because that the catalyst Pt could decrease the activation energy of gas chemisorption. As shown in Fig. 5b, the response/recovery speed of the as-prepared ZnO sensor was shortened from 108/285 to 6/19 s toward 50 ppm CO, which benefited from the addition of Pt decreasing the activation energy of gas reaction as well as accelerating the gas adsorption and desorption. Li et al. [90] prepared Pt/ZnO ordered porous monolayer films by a polystyrene sphere (PS) monolayer colloidal crystal template method. The Pt-decorated ZnO-based sensor showed an excellent response of 324 towards 100 ppm NH_3_, which was 200 times of the pure ZnO-based sensor. The mechanism of Pt-decorated ZnO toward NH_3_ is exhibited in Fig. 5c. When the as-prepared gas sensors were surrounded by air, the oxygen molecules were adsorbed on the material, extracting electrons, and thus forming the chemisorbed oxygen ions (O^−^, O_2_^−^). The spillover effect of Pt catalyzed the adsorbed oxygen molecules to produce more chemisorbed oxygen ions and reacts with more NH_3_ molecules, resulting in larger response. Another advantage of using Pt is the modulation of the electronic character of ZnO. The work function of Pt was 5.65 eV, which was higher than the ZnO (3.30 eV) Therefore, the electron transferred from ZnO to Pt NPs and then widened the depletion layers. Moreover, PtO_x_ and ZnO could construct a p-n heterojunction to enhance sensing performance, which could enhance the oxygen adsorption and modulate the conduction channel. The mechanism agrees well with the experimental results.

Compared to 1D or 2D structures, Pt-ZnO with 3D structures demonstrate enhanced performance owing to their large effective specific surface area. Liu et al. [72] prepared a hierarchical Pt-decorated ZnO microspheres with porous nanosheets through a hydrothermal strategy, followed by a reduction method. The as-prepared sensor exhibited high response towards 100 ppm triethylamine (TEA) of 242 at 200 °C, which was 50-fold enhancement for the pristine ZnO sensor. Notably, it had excellent selectivity to react with TEA gas while repressing other VOC gases, shown in Fig. 5d. Moreover, the DFT calculation indicated the adsorption energy of TEA on Pt-ZnO (−3.16 eV) was lower than ZnO (−1.21 eV), proving Pt-ZnO was of great advantages to sense TEA gas. In recent years, metal organic framework (MOF) structures have been introduced to improve the capability of electron transference between the target gas and the noble metal. For instance, Qin et al. [91] reported a Pt-decorated ZnO polyhedrons via an *in-situ* reduction method using with a template of zeolitic imidazolate framework-8 (ZIF-8). The synthesis process and the scanning electron microscopy (SEM) image of Pt@ZnO polyhedrons were exhibited in Fig. 5e–f, respectively. In contrast to the pure ZnO sensor, the obtained Pt@ZnO polyhedrons could not only promote the response value but also lower the temperature owing to the catalytic effect of Pt. The 2% Pt@ZnO sensor displayed good response of 10 to 50 ppm CO at 100 °C, high selectivity, and long-term stability. Notably, the 2% Pt@ZnO sensor showed an ultra-low detection limit (100 ppb), which was ascribed to the highly dispersed Pt NPs, enhancing the utilization rate of active sites. Overall, the introduction of the MOF structure effectively improved the dispersity of Pt NPs on the ZnO, thus enhancing the gas sensing behavior especially for detecting ppb-level gas. Zhou et al. [92] synthesized a 3D inverse opal (3DIO) microporous ZnO, further coated a ZIF-8 molecular sieve membrane through *in-situ* growth from the ZnO skeleton, and then functionalized with small Pt NPs. The microporous ZnO structure and the ZIF-8 filter membrane provided more active adsorption sites and eliminated the VOC interference, facilitating the gas sensing behaviors. Thus, this 3DIO Pt-decorated ZnO@ZIF-8 sensor exhibited great sensing behaviors that the response of the sensor was 118 toward 5.5 ppm H_2_S and the lowest H_2_S detection of sensor was 40 ppb. Moreover, the 3DIO Pt-decorated ZnO@ZIF-8 sensor was widely used in various fields, such as food quality evaluation, diagnosis of halitosis, and environment monitoring. For instance, as shown in Fig. 5g, the response of the 3DIO Pt-decorated ZnO@ZIF-8 sensor towards halitosis breath was 4.0, which was 2.4-fold enhancement of healthy counterparts (1.7), demonstrating that 3DIO Pt-decorated ZnO@ZIF-8 sensor could efficiently detect halitosis. Moreover, compared with untreated automobile exhaust gas, the response of 3DIO Pt-decorated ZnO@ZIF-8 sensor towards 1 L automobile exhaust with 330 ppb H_2_S was 3 times higher, indicating that it could be utilized to detect microscale concentrations of H_2_S. In summary, combining the MOF as outer filter membrane and Pt modification was an efficient strategy to develop an efficient sensor for trace H_2_S detection.

#### ***Pt-decorated SnO***_***2***_*** Gas Sensors***

**Fig. 6:**
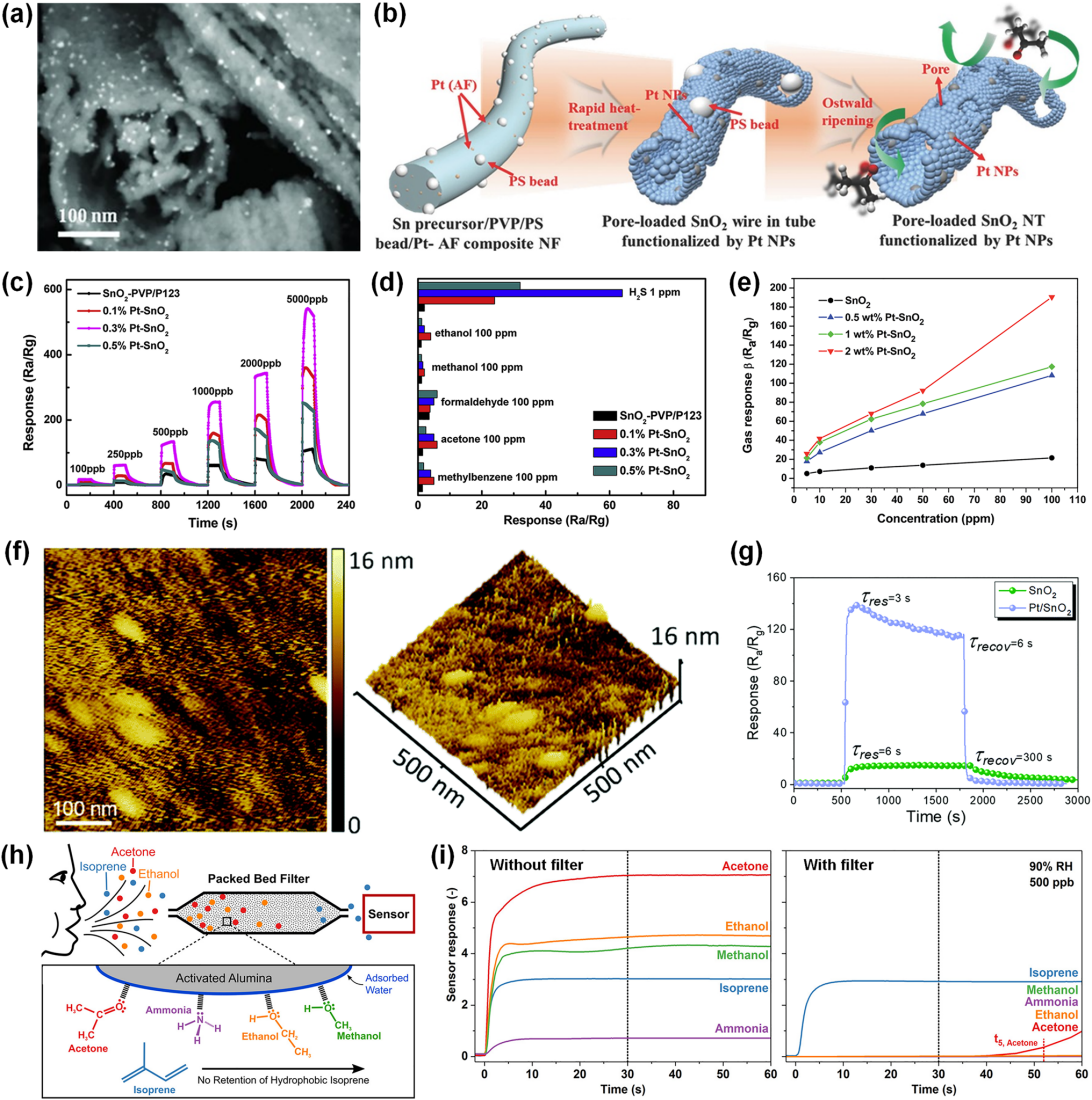
**a** Back scattered electrons (BSE) images of 20 wt% Pt-modified thin-wall assembled SnO_2_ fibers. Reproduced with permission from Ref. [95]. Copyright 2012, Wiley–VCH. **b** Synthesis of pore loaded SnO_2_ nanotubes decorated by Pt NPs. Reproduced with permission from Ref. [96]. Copyright 2016, Wiley–VCH. **c** Response versus time curves of the SnO_2_-PVP, 0.1% Pt-SnO_2_, 0.3% Pt-SnO_2_, and 0.5% Pt-SnO_2_ based-sensors to 100 ppb ~ 5 ppm H_2_S. **d** Selectivity behaviors of SnO_2_-PVP, 0.1% Pt-SnO_2_, 0.3% Pt-SnO_2_, and 0.5% Pt-SnO_2_ based-sensors [101]. Reproduced with permission from Ref. [101]. Copyright 2020, Elsevier. **e** Response of pure SnO_2_, 0.5 wt% Pt-decorated SnO_2_, 1 wt% Pt-decorated SnO_2_, and 2 wt% Pt-decorated SnO_2_ sensors towards various isopropanol concentrations at 220 °C. Reproduced with permission from Ref. [56]. Copyright 2014, Royal Society of Chemistry. **f** AFM characterization of Pt-SnO_2_ thin films. **g** Response and recovery behaviors of SnO_2_ and Pt-decorated SnO_2_ films sensor to 10 ppm TEA at 200 °C. Reproduced with permission from Ref. [104]. Copyright 2020, Royal Society of Chemistry. **h** Schematic of the well-designed filter-sensor system, exhibiting the adsorption of hydrophilic compounds on the activated alumina filter with no retention of hydrophobic isoprene. **i** Dynamic response curves of a Pt-SnO_2_ NPs sensor to 500 ppb isoprene, acetone, ethanol, methanol, and ammonia without and with an activated alumina filter at 90% RH. Reproduced with permission from Ref. [106]. Copyright 2018, American Chemical Society

As a typical n-type SMO, SnO_2_ is currently attracting tremendous attention owing to its environmentally friendly synthesis and wide application in the detection of various gases. In terms of various Pt decorated SnO_2_ nanostructures, 1D nanostructures like nanotubes and nanowires are efficient structures for SnO_2_ to detect gas owing to the specific surface area and high gas accessibility [93]. Electrospinning was utilized to synthesize 1D nanotubes, nanofibers, nanowires, and core–shell structures, due to its easy control of morphological [94]. It is suggested that various porous structures are obtained by changing the solution components and electrospinning conditions. For example, Bulemo et al. [55] reported Pt-decorated SnO_2_ hollow microbelts by electrospinning and calcination. The SnO_2_ hollow microbelts structure exhibited large specific surface area, small mean crystal size, and high porosity, which were advantages for promoting oxygen adsorption–desorption kinetics. The Pt (0.12%)-SnO_2_ hollow microbelts sensor exhibited a high response of 93.7 towards a low acetone concentration of 2 ppm, short response time (9.2 s), and long-term stability for over six-month. The better sensing behavior of Pt-SnO_2_ was owing to the specific surface area of microbelts and electrical and chemical sensitization of Pt. In addition, the Pt (0.12%)-SnO_2_ hollow microbelts-based sensor also showed high stability with response of 93.70 ± 0.89 to 2 ppm acetone for 25 repeated tests in a humid environment of 90% relative humidity (RH). Shin et al. [95] reported the hierarchical SnO_2_ fibers with wrinkled thin tubes produced by electrospinning and then decorated by Pt NPs via polyol method. The structure of Pt-decorated SnO_2_ fibers composed of wrinkled thin tubes is shown in Fig. 6a. The hierarchical SnO_2_ fibers with wrinkled thin tubes with high porosity not only promoted the decoration of Pt NPs, but also made gas molecules transfer to the entire sensing layer, shortening the response/recovery time less than 11/6 s. In conclusion, synthesizing the structures with high porosity and large specific surface area under various preparation conditions would facilitate the uniform distribution of Pt NPs, enhancing the sensing behaviors for the potential use of exhaled breath sensor. In addition, it is essential to uniformly decorate Pt NPs on SnO_2_ nanostructures to further maximize the catalyzation of Pt NPs. Jang et al. [96] synthesized the Pt-decorated meso/macro SnO_2_ nanotubes through an advanced electrospinning method, which is shown in Fig. 6b. In this work, the hollow protein nanocage was used to synthesize Pt NPs with different diameters distributing on SnO_2_ nanotubes. Thus, combining the porous nanotubes could lead to enhanced sensing performance because of the large specific surface area and high gas accessibility. The Pt decorated-SnO_2_ nanotubes sensor displayed high response of 192 to 5 ppm acetone at 350 °C.To sum up, electrospinning is an efficient method to synthesize the porous 1D structure composited with uniformly distributed Pt NPs, which is useful for developing excellent sensing behaviors.

Hydrothermal is a facile process of synthesizing 1D SnO_2_ nanostructures, which is widely used for fabricating gas sensors [97]. For example, Zhou et al. [98] fabricated Pt-modified SnO_2_ nanoneedles through a hydrothermal method. For 3.125 at% Pt- modified SnO_2_ nanoneedle composites, a high sensing response of 23.18 is recorded towards 100 ppm CO gas at 250 °C with the response/recovery speed of 15/14 s, respectively. Chen et al. [99] synthesized SnO_2_ nanorods via hydrothermal strategy, followed by the ultraviolet (UV)-irradiated photochemical reduction method to functionalize Pt NPs. With decoration of 3.63% Pt NPs, the response of sensor toward 1000 ppm H_2_ was enhanced from 1.4 to 7.9 at room temperature (RT). Generally speaking, sensors work at RT are attractive for reducing cost and improving stability, but they often need long response time and recovery time, limiting their practical application [100]. Functionalizing of Pt NPs on the SnO_2_ could promote the oxygen adsorption, which are spilled over on the SnO_2_ to react with more gas molecules. Moreover, Pt NPs were beneficial to the electrons transfer owing to the higher work function than the SnO_2_. The Pt-SnO_2_ nanorods-based sensor responded to 1000 ppm H_2_ with short response time (0.4 s), owing to the sensitization effect of Pt. Therefore, functionalizing Pt NPs was potential to enhance the response speed of RT sensors. In addition, Sun et al. [101] reported a Pt-decorated SnO_2_ mesoporous nanoflowers H_2_S sensor working at RT through a mixed template method followed by selective calcination. As exhibited in Fig. 6c, the response towards ppb-level H_2_S at RT was significantly improved by decorating Pt NPs. The 0.3 wt% Pt-decorated SnO_2_ nanoflowers sensor showed high response of 68 towards 1 ppm H_2_S at 30 °C, which was 11-time improvement of pristine SnO_2_ sensor. Meanwhile, as shown in Fig. 6d, the decoration of Pt NPs significantly improved the selectivity of Pt-SnO_2_ sensors compared with pristine SnO_2_ sensor. The catalytic Pt NPs could effectively promote electron transfer, thus accelerating the reaction not only between chemisorbed oxygen and H_2_S but also between SnO_2_ and H_2_S molecules ($Sn{O}_{2}+{H}_{2}S\to Sn{S}_{2}+{H}_{2}O$). Therefore, the selectivity of Pt-decorated SnO_2_ sensors were further enhanced. Besides, the sensor also displayed short response/recovery time of 11/75 s. The enhanced sensing behavior was due to the porous structure with high specific surface area and the catalyzation effects by Pt NPs. In summary, the surface decoration of Pt is a promise method to promote the gas sensing behaviors at the field of RT H_2_S gas sensors.

There are various techniques to fabricate 2D SnO_2_ structures. Solution combustion synthesis has been regarded as an efficient, rapid, and energy-effective method for producing large-scale nanostructures [102]. For instance, Dong et al. [56] synthesized Pt-decorated SnO_2_ porous sheets with different Pt concentration via solution combustion method. The 2 wt% Pt-decorated SnO_2_ sensor exhibited the highest response to a wide range concentration (5 ~ 100 ppm) of isopropanol gas, as displayed in Fig. 6e. Specifically, the response of the 2 wt% Pt–decorated SnO_2_ sensor towards 100 ppm isopropanol was 190.50, which was 9-time enhancement of the pure SnO_2_ based sensor. The enhanced response was ascribed to the electronic sensitization as well as the catalytic oxidation of Pt NPs. Atomic layer deposition (ALD) technique has a great advantage of precisely controlling the film thickness at the atomic level [103], which is beneficial to prepare efficient gas sensors. Besides, Xu et al. [104] reported Pt decorated SnO_2_ films sensor by ALD with different thicknesses varying from 4 to 18 nm. The morphologies of pristine SnO_2_ and Pt-decorated SnO_2_ film were analyzed by atomic force microscopy (AFM). The Pt-SnO_2_ film exhibited in Fig. 6f had a surface root mean square roughness of 420 pm, which was similar with SnO_2_ thin film (470 pm), indicating both have similar surface features. Thus, ALD technique could synthesize thin film with uniform thickness, which was advantageous to fabricate stable sensing layers. The Pt-decorated SnO_2_ ultrathin film sensor could reduce the working temperature from 260 to 200 °C. The Pt-decorated 9 nm SnO_2_ film sensor exhibited high response of 136.2 to 10 ppm TEA, which was 9 times improvement to the pure SnO_2_ sensor, and ultrafast response/recovery speed (3/6 s), as shown in Fig. 6g. Moreover, the Pt-decorated 9 nm SnO_2_ film sensor could detect the TEA as low as 7 ppb, illustrating the high potential development for sensitive detection of VOCs at ppb-levels. Owing to the appropriate thickness of SnO_2_ film synthesized by ALD, oxygen vacancies in films, and catalyzation of single atom Pt, the Pt-SnO_2_ thin film sensor showed an outstanding sensing performance.

Moreover, since selectivity is considered as one of the most crucial aspects for gas sensors in practical applications surrounded with diverse interfering gases, many efforts on constructing an efficient filter have also been devoted to further improve the selectivity of Pt-decorated SnO_2_ gas sensors. The filters, including sorption, size-selective, and catalytic filters, will help to change the composition and/or concentration of molecules in gas mixtures before reaching the sensor as expected [105], thus contributing to the enhancement of selectivity. For example, Broek et al. [106] designed an effective filter consisted of a packed bed of activated alumina powder with high porosity upstream of the flame-made Pt-SnO_2_ NPs sensor (Fig. 6h), achieving highly selective and rapid breath isoprene detection at the ppb-level and high humidity. Specifically, as shown in Fig. 6h, the well-designed filter can adsorb hydrophilic breath analytes like acetone, ethanol, methanol, and ammonia, as well as water molecules, leaving hydrophobic isoprene unhindered and transported to the downstream sensor. Finally, the well-designed filter-sensor system exhibited remarkable selectivity (> 100) to ppb-level isoprene at 90% RH in Fig. 6i, which was significantly enhanced comparing to the pristine nonspecific Pt-SnO_2_ NPs sensor. In addition, Oliaee et al. [107] reported a catalytic filter comprised of Au NPs decorated Fe_2_O_3_ for the Pt-SnO_2_ sensor and realized highly selective detection to propane or methane in the presence of CO and ethanol. Actually, CO will be completely converted to insensitive CO_2_ by the catalytic filter at room temperature, and ethanol will be adsorbed/oxidized by the filter at room temperature/temperatures higher than 200 °C. Similarly, a Au NPs-promoted Ce_0.8_Zr_0.2_O_2_ catalytic filter was designed for the Pt-SnO_2_ sensor by Fateminia et al. [108], demonstrating selective detection of ethanol and methane in the presence of interfering gases. From their observations, the operating temperatures of the filter and the sensor will both affect the selectivity of the filter-sensor system, which require meticulously selection and control for different applicaion scenarios.

#### ***Pt-decorated WO***_*3*_*** Gas Sensors***

WO_3_ is an important n-type SMO with a wide band gap (2.8 eV), which is a potential material for developing excellent gas sensors toward various gases due to its excellent physical and chemical properties and superior sensing characteristics [109]. Functionalization of WO_3_ with Pt NPs could enhance the sensing performance of the resultant material due to the catalytic properties of Pt.

**Fig. 7:**
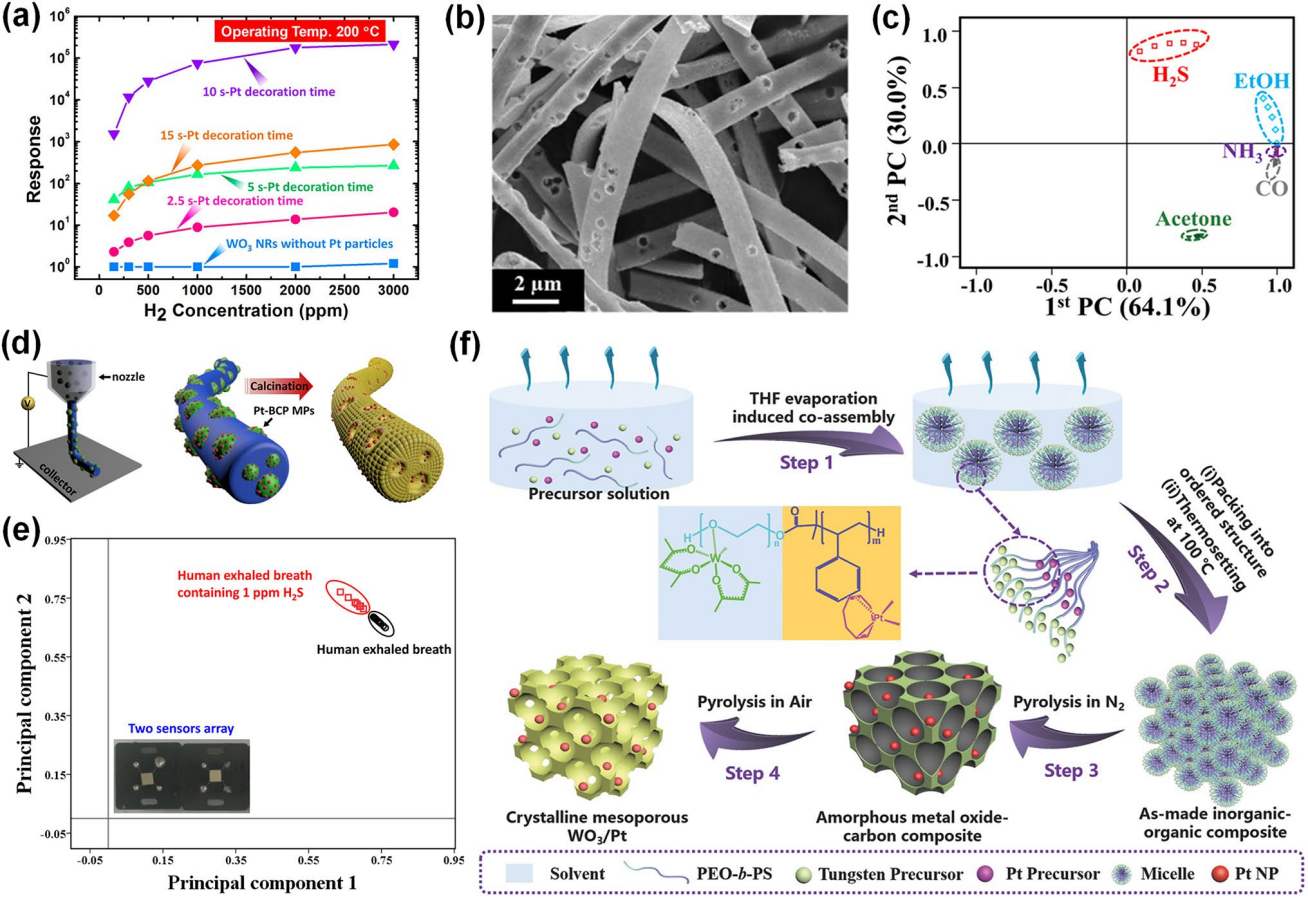
**a** Response of different Pt decoration times decorated WO_3_ nanorods sensors toward 100–3000 ppm of H_2_ at 200 °C. Reproduced with permission from Ref. [53]. Copyright 2014, American Chemical Society. **b** SEM image of meso- and macroporous Pt-decorated WO_3_ microbelts. **c** PCA result of the sensor array. Reproduced with permission from Ref. [113]. Copyright 2018, American Chemical Society. **d** Synthesis route of Pt-decorated macroporous WO_3_ nanofibers. **e** PCA result of the healthy bodies and halitosis patients through the human exhaled breath. Reproduced with permission from Ref. [114]. Copyright 2016, American Chemical Society. **f** Fabrication process of Pt loaded ordered mesoporous WO_3_ composites. Reproduced with permission from Ref. [32]. Copyright 2017, Wiley–VCH

Pt-decorated WO_3_ is often used for detecting H_2_ due to the ability of Pt to dissociate hydrogen atoms effectively at RT [110]. Horprathum et al. [53] successfully developed Pt-loaded WO_3_ nanorods through the glancing angle deposition (GLAD) method. As exhibited in Fig. 7a, the high response of the optimal Pt-loaded WO_3_ nanorods sensor was 2.2 × 10^5^ (*R*_a_/*R*_g_) towards 3000 ppm H_2_ at 200 °C, while pristine WO_3_ nanorods sensor was almost no response, indicating that Pt decoration was an efficient method to promote the H_2_ detection. Moreover, the optimal Pt-loaded WO_3_ sensor showed high sensitivity to 150 ppm at 150 °C and high selectivity toward H_2_ among various gases. The improved sensing behavior was ascribed to the chemical sensitization of Pt, which dissociated H_2_ molecules into H atoms and reacted with WO_3_ through spillover effect, decreasing the depletion width and electrical resistance. Fan et al. [110] developed a Pt-decorated WO_3_ nanorods sensor through evaporation method and sputtering process. The Pt-decorated WO_3_ sensor exhibited very fast response of 80 ms toward pure H_2_ at 110 °C due to the chemical sensitization of Pt. Nishijima et al. [111] deposited Pt-WO_3_ nano/micro powder films on a silica glass substrate using nanosecond pulsed laser ablation. They reported that the low limit of H_2_ detection (10 ppm) and short response time of 20 s towards 40,000 ppm H_2_ was obtained.

Pt-decorated WO_3_ sensors are also utilized for effectively detecting H_2_S gas. Yao et al. [112] synthesized Pt-decorated WO_3_ nanorods hydrothermal and chemical reduction methods. The response of the 0.2 at% Pt-decorated WO_3_ sensor was 1,638 towards 10 ppm H_2_S at 200 °C, with the short response/recovery time of 42/37 s, respectively. In comparison with pristine WO_3_ sensor, Pt-WO_3_ sensor exhibited enhanced gas sensing behavior because of the electronic modulation and its catalysis reaction between H_2_S and oxygen ions. The porous microstructure has advantages of numerous reaction sites and gas diffusing channels, leading to better response performance. In this regard, Kim et al. [113] reported Pt-decorated WO_3_ microbelts with a large amount of porosity via the electrospinning combined with sacrificial templates and subsequent calcination. Figure 7b showed the structure of meso- as well as macroporous Pt-modified WO_3_ microbelts. Moreover, Pt NPs were fabricated by using biological protein cages, such as apoferritin to prevent the agglomeration of particles after calcination, thus further improving the gas sensing properties. The 0.05 wt% Pt-decorated WO_3_ microbelts sensor displayed excellent sensing performance including high response of 372 towards 5 ppm H_2_S in 95% humid condition and excellent selectivity to H_2_S among other gases, which were owing to the bimodally porous nanostructure and catalysis effect of Pt NPs. Furthermore, as exhibited in Fig. 7c, the principal component analysis (PCA) result showed that the sensor arrays could clearly classify H_2_S from the other gases without overlapping, indicating the potential application of the sensor arrays to detect H_2_S to diagnose halitosis. In addition, Choi et al. [114] developed Pt decorated microporous WO_3_ nanofibers via electrospinning technique and calcination. In addition, Pt infiltrated block copolymers microparticles (Pt-BCP MPs) were made through an oil-in-water emulsion technique, which could be evenly distributed on the WO_3_, further enhancing the gas sensing behavior. The fabrication process of Pt-modified macroporous WO_3_ nanofibers is shown in Fig. 7d. The 0.042 wt% Pt-WO_3_ nanofibers sensor exhibited an excellent response of 834.2 ± 20.1 toward 5 ppm H_2_S at 350 °C in a high humid condition (95% RH) and low H_2_S detection of 100 ppb. The PCA result indicated that the sensor arrays achieve distinguish pattern recognition of various gases, demonstrating their high selectivity. Moreover, as shown in Fig. 7e, the healthy breath and the halitosis breath was obviously distinguished, indicating the sensor arrays was a potential method for the breath diagnosis. In this regard, the porous structure and catalytic character of uniformly distributed Pt NPs are crucial to enhance the sensing performance to target gases.

In addition, Pt-loaded WO_3_ sensors are reported to detect other gases including CO, NO_2_, acetone, and ammonia. Ma et al. [32] synthesized Pt-decorated highly ordered mesoporous WO_3_ via a multicomponent co-assembly method. The fabrication process of the Pt-decorated WO_3_ nanocomposite was shown in Fig. 7f. Owing to the sensitizing effect of the Pt NPs, the Pt-WO_3_ sensor showed the response of 10 to 100 ppm CO at 125 °C, short response/recovery speed (16/1 s), and remarkable selectivity. Besides, Liu et al. [115] reported a Pt decorated WO_3_ thin film sensor based on the MEMS devices through GLAD and conventional planar deposition. The Pt-decorated WO_3_ film sensor displayed high response of 1,308.26 toward 10 ppm NO_2_, low detection of limit (80 ppb), and excellent selectivity to NO_2_ among NH_3_, CO, acetone, and ethanol. Alev et al. [54] fabricated WO_3_ nanoflakes through a hydrothermal route and further loaded Pt NPs via a sputtering method. The catalytic effect of Pt could dramatically enhance the gas sensing behaviors of Pt-WO_3_ sensor which could detect acetone even at a low concentration of 237 ppb at 250 °C. Chao et al. [116] fabricated Pt loaded square-like WO_3_ via a facile hydrothermal and reduction method. It was found that the response of the 1 wt% Pt-decorated WO_3_ sensor was 100.09 towards 1000 ppm NH_3_ with short response/recovery time (8 /11 s), low detection of limit (1 ppm), and long-term stability (70 days). In summary, Pt functionalized WO_3_ sensor exhibits improved sensing properties toward target gases due to the sensitizing effect of Pt.

#### Pt-decorated Other SMOs-Based Gas Sensors

Apart from ZnO, SnO_2_, and WO_3_, there are various other metal oxide semiconductors like In_2_O_3_, NiO, CuO, and Fe_2_O_3_ have been investigated to enhance the gas sensing performance by decorating Pt NPs.

**Fig. 8:**
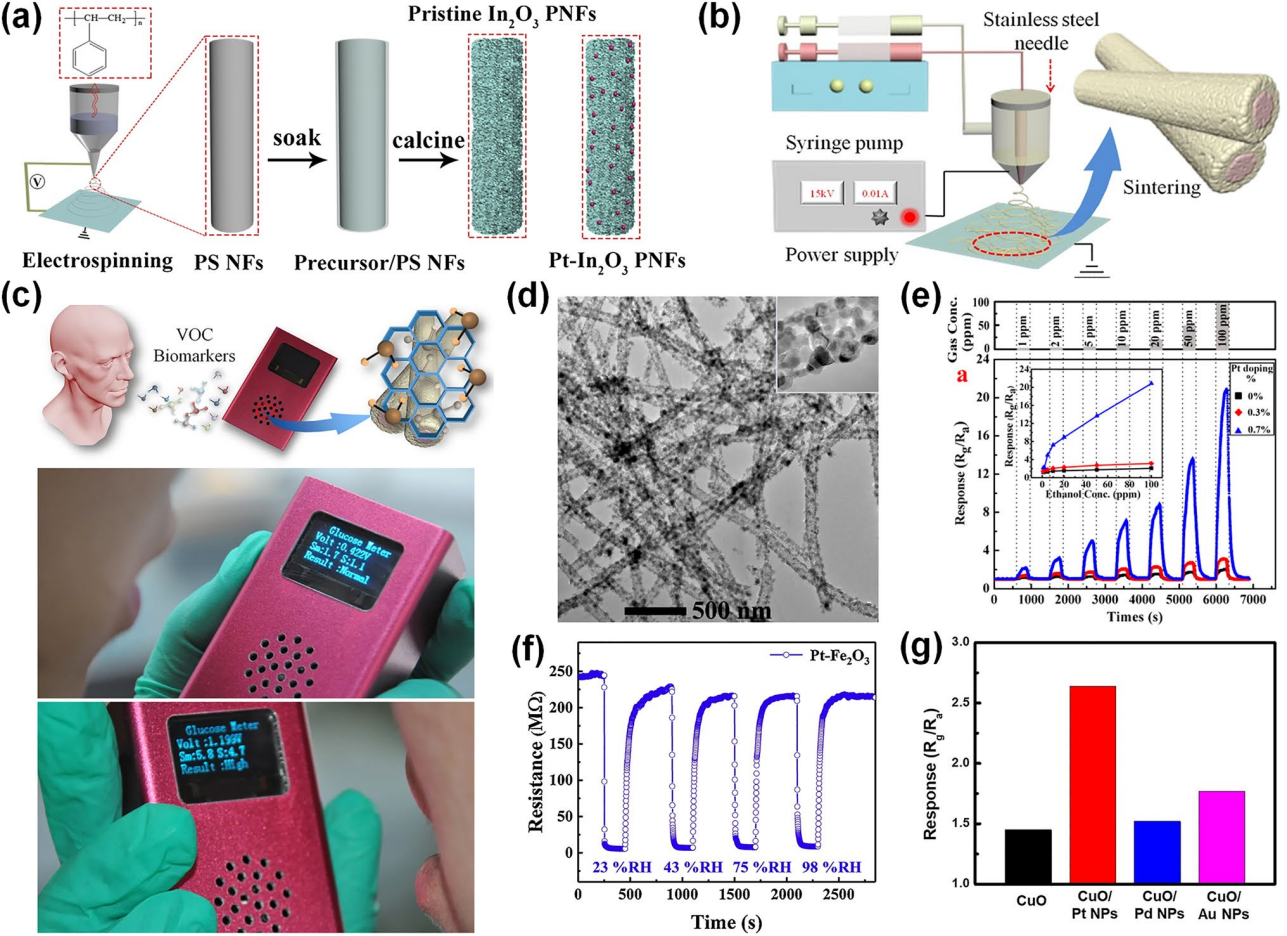
**a** Synthesis process of pristine In_2_O_3_ porous nanofibers and Pt-decorated In_2_O_3_ porous nanofibers. Reproduced with permission from Ref. [120]. Copyright 2019, Elsevier. **b** Fabrication process for Pt-In_2_O_3_ nanowires. **c** Schematic diagram of a portable device including the Pt-decorated In_2_O_3_ nanowire sensor. Reproduced with permission from Ref. [121]. Copyright 2018, Springer Nature. **d** TEM image of Pt-decorated NiO nanotubes. **e** Response curves of the pristine NiO, 0.3% Pt-decorated NiO, and 0.7% Pt-decorated NiO nanotube gas sensors towards different concentration of ethanol varying from 1 to 100 ppm at 200 °C; the inset image displayed linear relation of the response and the gas concentration. Reproduced with permission from Ref. [122]. Copyright 2013, American Chemical Society. **f** Resistance changes of Pt-Fe_2_O_3_ to acetone under different conditions. Reproduced with permission from Ref. [126]. Copyright 2019, Elsevier. **g** Responses of pristine CuO, Pt-CuO, Pd-CuO, and Au-CuO-based sensors to 100 ppb HCHO at 225 °C. Reproduced with permission from Ref. [128]. Copyright 2019, Elsevier

In_2_O_3_ with wide band gap (3.6 eV), is a potential gas sensing material owing to its high electrical conductivity and stability [117]. In particular, Pt functionalized 1D In_2_O_3_ structures were widely used for detecting NO_2_ and acetone. Liu et al. [118] fabricated Pt-loaded mesoporous nanofibers through electrospinning and subsequent reduction method. Because of the porous microstructure, specific surface area, and catalytic effect of Pt, the Pt-In_2_O_3_ mesoporous nanofibers sensor could detect NO_2_ at as low as 10 ppb at 40 °C (*R*_g_/*R*_a_ = 2.8). Lee et al. [119] synthesized Pt decorated In_2_O_3_ nanorods through vapor–liquid-solid growth and calcination treatment. The Pt-modified In_2_O_3_ nanorods calcined at 600 °C displayed highest response towards NO_2_, which was 7 times improvement of the pure In_2_O_3_ nanorods. Liu et al. [120] reported a Pt-loaded In_2_O_3_ porous nanofibers sensor fabricated through electrospinning method and calcination, as exhibited in Fig. 8a. Due to the spillover effect of Pt NPs and the large specific surface area of the porous 1D structure, the Pt-In_2_O_3_ sensor displayed improved gas sensing properties towards 1 ppm acetone with high response of 15.1 at 180 °C with fast response/recovery speed (6/9 s) and excellent long-term stability (50 days). In addition, Liu et al. [121] fabricated Pt-In_2_O_3_ core–shell nanowires via co-electrospinning method, as displayed in Fig. 8b. The Pt-In_2_O_3_ core–shell nanowires-based sensor exhibited enhanced sensing performance with a high response up towards 27 to 10 ppm acetone which was six-fold enhancement of pristine In_2_O_3_ nanofibers. Beyond that, a Santa Barbara Amorphous-15 (SBA-15) molecular sieve containing 2D hexagonal channels was employed on top of the sensing layer, effectively reducing the impact of humidity on the sensor. Thus, the as designed sensor could be potential to detect microscale acetone in exhaled breath. The Fig. 8c shows a portable sensing device include the Pt-In_2_O_3_ nanowires sensor with the moisture filter layer. In summary, Pt decorated In_2_O_3_ 1D nanostructures is highly sensitive to NO_2_ and acetone, which is an effective strategy to diagnose diseases through exhaled breath detection.

NiO has been extensively investigated because of its important qualities such as wide energy bandgap (~ 3.8 eV), high chemical stability, and unique electrical properties [122]. The sensors based on Pt functionalized 1D NiO structures show improved sensing behaviors owing to their high specific surface area and electrical and chemical sensitization of Pt NPs. For instance, Fu et al. [122] synthesized Pt-decorated NiO composite nanotubes via a facile electrospinning method. The morphology of Pt-decorated NiO nanotubes is shown in Fig. 8d. The Pt-decorated NiO nanotubes sensor displayed the response to 100 ppm ethanol of 20.85 at 200 °C, which was tenfold enhancement of pristine NiO based sensor (2.06), as shown in Fig. 8e. Wu et al. [123] prepared Pt@NiO NPs through sol–gel method and investigated its sensing performance. The response of core–shell Pt@NiO (4.25) to 5000 ppm H_2_ was higher than that of NiO (1.02) with rapid response/recovery speed (91/8 s) at RT. In addition, Pt decorated 2D NiO structures sensors were widely employed owing to their numerous active sites during the adsorption and desorption of target gaseous species. For instance, Chen et al. [124] synthesized NiO thin film via radio frequency (RF) sputtering and then deposited Pt film via thermal evaporation followed by an annealing process. The Pt/NiO thin film-based sensor exhibited improved sensing behavior including high response of 13.75 towards 1000 ppm NH_3_ at 300 °C with short response/recovery time (15/76 s) and low limit of detection of 10 ppb. Liang et al. [125] synthesized Pt loaded NiO nanosheets through hydrothermal and photo reduction method. The enhanced sensing behaviors of Pt-decorated NiO porous nanosheets could be attributed to the synergistic effect and facer-selective Pt decoration. In summary, Pt decoration is regarded as a useful way to enhance the sensing behaviors of NiO gas sensors.

Moreover, Pt functionalized Fe_2_O_3_ and CuO gas sensors are often used to detect VOCs. For instance, Zhang et al. [126] reported Pt-decorated Fe_2_O_3_ nanocubes via hydrothermal and reduction method. Compared with pristine Fe_2_O_3_, the Pt-loaded Fe_2_O_3_ composites exhibited a higher response of 25.7 towards 100 ppm acetone at a 139 °C, with rapid response/recovery speed of 3/22 s. The sensitizing effect of Pt was crucial to the acetone detection. Besides, the sensor was investigated to detect acetone under various environment conditions. As displayed in Fig. 8f, the resistance changes of the Pt-Fe_2_O_3_ sensor to 100 ppm acetone were very similar under four conditions mimicking exhaled breath. Guo et al. [127] synthesized Pt-decorated Fe_2_O_3_ nanowires with the Pt content varying from 0.5 to 3 mol% via homotaxial electrospinning method. The 1 mol% Pt-decorated Fe_2_O_3_ nanowires was demonstrated to have the best sensing behavior with high response of 157 to 10 ppm H_2_S at 175 °C due to the catalytic character of Pt and high specific surface area of Fe_2_O_3_ nanowires. As for Pt decorated CuO-based sensor, Lee et al. [128] produced porous CuO structure derived from MOFs of copper benzene-1,3,5-tricarboxylate (HKUST-1), and then decorated Pt NPs with 1 ~ 2 nm diameters via sonochemical synthetic process. The 0.06 wt% Pt-decorated CuO exhibited high response to HCHO (2.64 at 100 ppb) at 225 °C, and the linear relation between the concentration and the response. Due to the porous structure derived from MOF and the sensitizing effect of Pt NPs, the Pt@CuO-based sensor achieved better sensing performance. Moreover, Pd and Au NPs could be decorated on CuO using the same method. Figure 8g shows that the response for Pd-CuO and Au-CuO to 100 ppb HCHO were 1.52 and 1.77, respectively, much lower than that of Pt-CuO, demonstrating that Pt is the best choice to functionalize CuO for HCHO detection.

#### Pt-decorated Heterostructured SMOs-based Gas Sensors

**Fig. 9:**
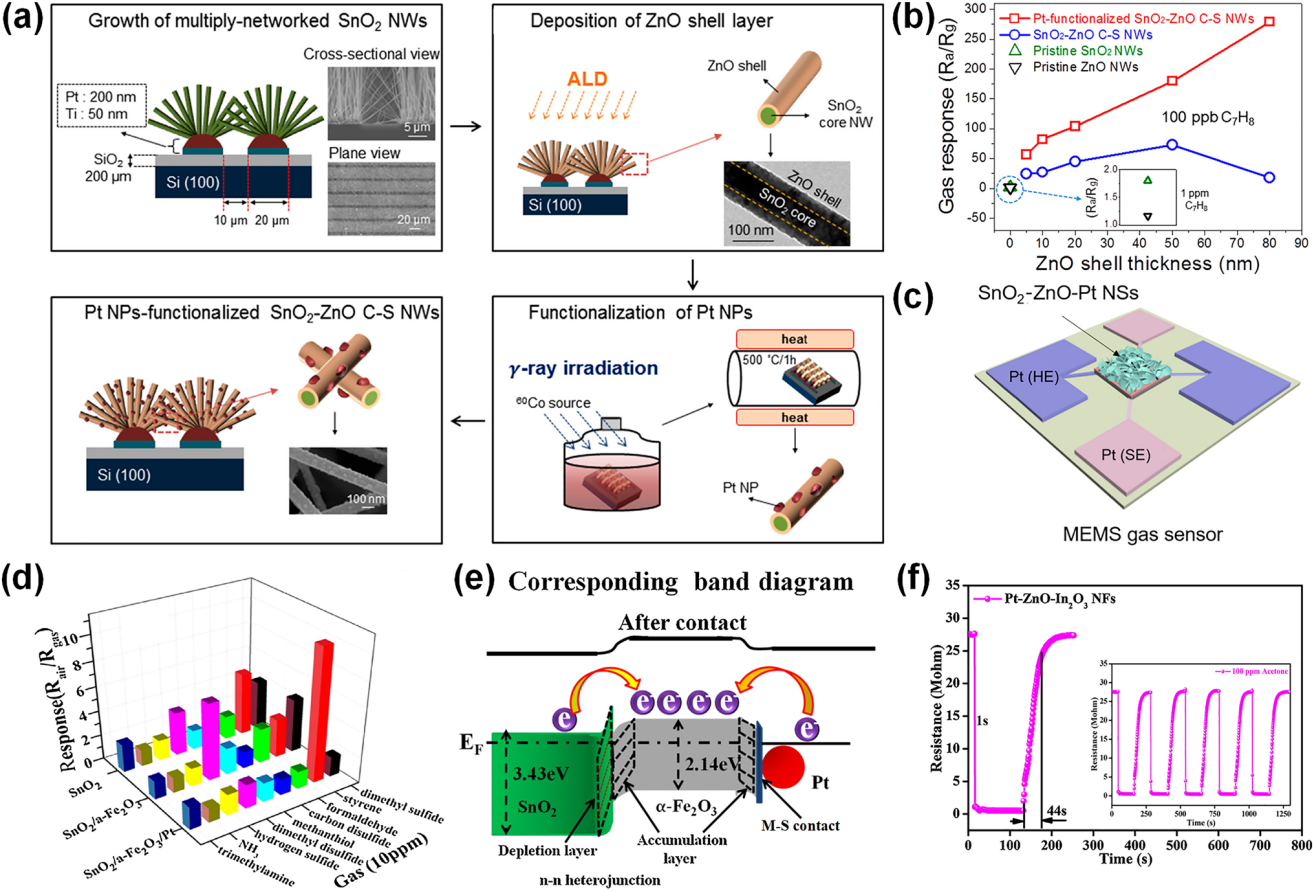
**a** Schematic illustrations of the fabrication process of the Pt-decorated SnO_2_/ZnO nanowires. **b** Responses of the Pt-decorated SnO_2_/ZnO nanowires, SnO_2_/ZnO nanowires, pristine SnO_2_ nanowires, and pristine ZnO nanowires-based sensor to toluene. Reproduced with permission from Ref. [130]. Copyright 2015, American Chemical Society. **c** Synthesis scheme of Pt-decorated SnO_2_-ZnO core–shell nanosheets *in-situ* on MEMS. Reproduced with permission from Ref. [131]. Copyright 2022, American Chemical Society. **d** Selectivity of pristine SnO_2_, SnO_2_-α-Fe_2_O_3_, and Pt-decorated SnO_2_-α-Fe_2_O_3_ sensors to various gases (10 ppm) at 206 °C. **e** Energy band diagram of Pt-SnO_2_-α-Fe_2_O_3_. Reproduced with permission from Ref. [132]. Copyright 2018, Elsevier. **f** Response/recovery speed of Pt-decorated ZnO/In_2_O_3_ sensor towards 100 ppm acetone at 300 °C, the inset is the five periods of response and recovery curves. Reproduced with permission from Ref. [134]. Copyright 2018, Elsevier

Designing hybrid structures with SMO is an efficient method to promote gas sensing behaviors. In particular, core–shell structure has been extensively utilized to enhance sensing response because of the formation of a depletion layer [129]. Moreover, the attachment of Pt NPs to the core–shell structure can modulate its resistance and further enhance the sensing behavior. For instance, Kim et al. [130] fabricated SnO_2_-ZnO core–shell nanowires through ALD and attached Pt NPs via γ-ray radiolysis, as shown in Fig. 9a. The Pt-decorated SnO_2_-ZnO core–shell nanowires based-sensor realized an extraordinarily high response of 279 to 100 ppb toluene at 300 °C, which was higher than that of SnO_2_-ZnO core–shell nanowires, pristine SnO_2_, and pristine ZnO sensor, as shown in Fig. 9b. The enhancement of the toluene sensitivity was depended on the formation of the electron-depleted region and the catalytic effect of Pt NPs. Wu et al. [131] synthesized SnO_2_-ZnO core–shell nanosheets through ALD and hydrothermal route, and then deposited Pt NPs via magnetron sputtering. Moreover, the sensing materials were fabricated on the MEMS devices which had many advantages including low power consumption, easy integration, and large-scale, as shown in Fig. 9c. The Pt-decorated SnO_2_-ZnO core–shell nanosheets-sensor exhibited the response of 30.43 towards 5 ppm H_2_S at 375 °C with high selectivity among NO_2_, NH_3_, ethanol, and CH_4_. The improved sensing behavior could be attributed to the heterojunctions and the sensitization effect of Pt NPs. In summary, core–shell heterogenous nanostructures functionalized with Pt NPs demonstrate great potential for developing excellent gas sensors towards various target gases.

In addition, many heterojunctions functionalized Pt NPs were also developed for remarkable gas sensors. For example, Liu et al. [132] fabricated SnO_2_-α-Fe_2_O_3_ hollow nano-heterojunctions via hydrothermal and then loaded ultrafine Pt NPs through *in-situ* reduction and subsequent calcination treatment. The 6.45 wt% Pt embellished SnO_2_-α-Fe_2_O_3_-based sensor in Fig. 9d exhibited outstanding gas sensing behavior with a high response of 10.56 to 10 ppm styrene with ultrafast response/recovery speed (3/15 s), low limit of detection (50 ppb), and excellent selectivity among other malodorous gases. Compared to pure SnO_2_ and SnO_2_-α-Fe_2_O_3_ sensors, the improvement in styrene sensing behavior of the Pt embellished SnO_2_-α-Fe_2_O_3_ sensor could be mainly attributed to the electronic sensitization. As exhibited in Fig. 9e, the n–n heterojunction led to band bending at the interface of SnO_2_-α-Fe_2_O_3_ and the formation of metal–semiconductor contact could induce band bending at the interface of Pt-α-Fe_2_O_3_. Thus, the electron would move from SnO_2_ to α-Fe_2_O_3_ until the Fermi level was balanced, leading to the 6electron accumulation layer in α-Fe_2_O_3_ which could adsorb more oxygen molecules and modulate its resistance. Simultaneously, the metal–semiconductor contact would migrate electron from Pt to α-Fe_2_O_3_, further increasing the electron concentration of α-Fe_2_O_3_ side. Therefore, these two factors could facilitate the adsorption of oxygen molecules on the α-Fe_2_O_3_ side and significantly change the resistance of Pt-SnO_2_-α-Fe_2_O_3_. Besides, the catalytic effect of Pt NPs was crucial to the styrene sensing owing to its selective catalytic oxidation of styrene. Overall, it is promising to design n–n heterogenous nanostructures functionalized Pt NPs in the detection of specific gases. In addition, Chang et al. [133] synthesized In_2_O_3_-WO_3_ nano powder via calcination method and then functionalized Pt NPs through reduction process. The obtained Pt functionalized In_2_O_3_-WO_3_ nano powder-based sensor could achieve ppb-level detection of nitric oxide (NO) at RT due to the n–n heterojunction of In_2_O_3_ and WO_3_ and the catalytic effect of Pt NPs. In particular, the 0.25% Pt-decorated In_2_O_3_-WO_3_ nano powder-based sensor exhibited high response of 330 and 15.2 to 0.1 ppm and 25 ppb NO, respectively. In addition, Guo et al. [134] prepared Pt functionalized ZnO-In_2_O_3_ nanofibers through using ZIF-8 and electrospinning method. Owing to the noble metal NPs could be encapsulated in the cavity of ZIF-8 resulting in ultra-small nanometers, it was found that the average diameter of Pt NPs was only 3 nm which could maximize their catalytic effect. As exhibited in Fig. 9f, the Pt-decorated ZnO-In_2_O_3_ sensor displayed an excellent gas sensing behavior toward acetone with the response of 57.1 to 100 ppm at 300 °C, short response/recovery speed (1/44 s), and low limit of detection (500 ppb). The improved sensing properties was attributed to: (i) the expanded electron depletion layer on the In_2_O_3_ caused by the n–n nano-heterojunctions, (ii) the sensitizing effect of decorated ultra-small Pt NPs, and (iii) the increased sensor resistance induced by the p–n heterojunction. In summary, MOF could be utilized as an efficient platform to produce ultra-small catalysts decorated on SMOs, promoting the development of highly sensitive gas sensors.

### Pd-Decorated SMOs-based Gas Sensors

#### Pd-Decorated ZnO Gas Sensors

**Fig. 10:**
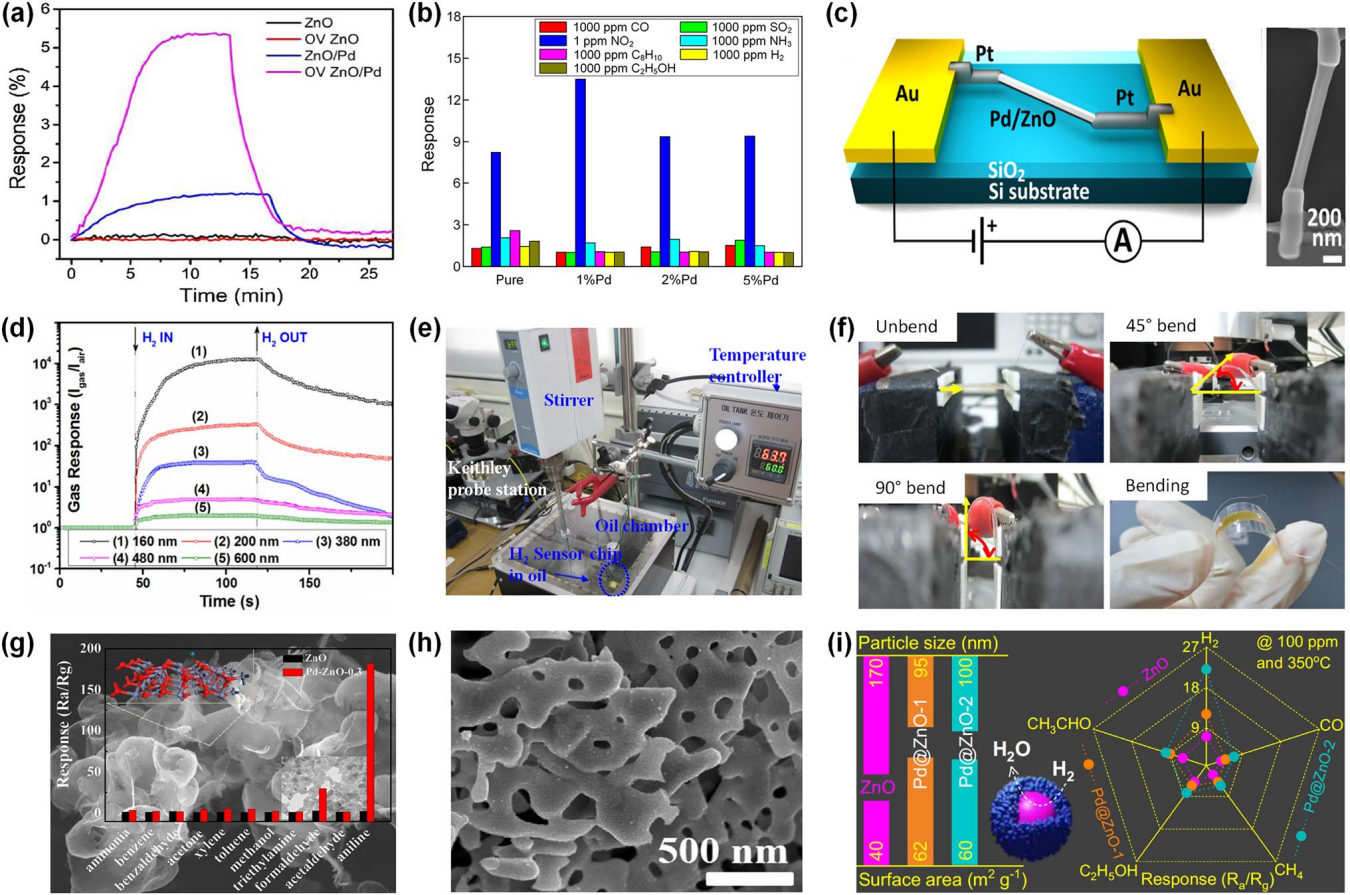
**a** Transient response of the sensors to 0.1% CH_4_ based on the ZnO, OV ZnO, ZnO/Pd and OV ZnO/Pd composites under 590 nm light illumination. Reproduced with permission from Ref. [138]. Copyright 2020, Elsevier. **b** Selectivity of the sensors based on pristine (@150 °C) and Pd-ZnO nanowires (@100 °C) to different gases. Reproduced with permission from Ref. [140]. Copyright 2019, Elsevier. **c** Schematic diagram of the Pd-ZnO nanowires-based nanosensor device. **d** Transient response of the sensors based on single Pd-ZnO nanowire with different diameters. Reproduced with permission from Ref. [141]. Copyright 2018, Elsevier. **e** Measurement system for the detection of dissolved H_2_ in transformer oil. Reproduced with permission from Ref. [144]. Copyright 2016, Elsevier. **f** Optical images of the flexible RT H_2_ sensor based on Pd/ZnO nanorods under different bending angles. Reproduced with permission from Ref. [145]. Copyright 2013, Elsevier. **g** Selectivity of the sensors based on ultrathin agaric-like Pd-decorated ZnO nanosheets with the background of Pd/ZnO SEM image. Reproduced with permission from Ref. [148]. Copyright 2020, American Chemical Society. **h** SEM image of porous coral-like Pd-decorated ZnO nanosheets. Reproduced with permission from Ref. [149]. Copyright 2021, Elsevier. **i** Selectivity performance and BET surface area of the Pd@ZnO core–shell NPs. Reproduced with permission from Ref. [150]. Copyright 2021, Elsevier

Various morphologies of nanostructures for ZnO including nanorods, nanowires, nanofibers, nanosheets, agaric-like, coral-like, and core–shell nanostructures have been fabricated and employed in Pd-decorated gas sensors to help increasing the selectivity and response value, reducing operating temperature, and shortening the response and recovery time. Gao et al. [135] fabricated Pd-decorated ZnO nanorods through the impregnation process and proposed that the Pd/PdO_x_ covered on the ZnO surface has a significant enhancement on H_2_ sensing performance. The effect of Pd^0^ and PdO_x_ content under different temperature was further studied using *in-situ* Raman technique and found out that the appropriate variation of Pd/PdO_x_ ratio can effectively improve the response value and shorten the response/recovery time of pure ZnO sensor. Cao et al. [136] grew perpendicularly aligned ZnO nanorods by chemical vapor deposition (CVD) method, followed by precise decoration of Pd NPs on their surface through magnetic sputtering and annealing process. The response of Pd-decorated ZnO nanorods sensor to ethanol was significantly enhanced owing to the catalytic effect of Pd NPs accelerating the dissociation and the chemisorption of oxygen. In addition, the Pd-decorated ZnO nanorods also respond well to trimethylamine. Meng et al. [137] reported Pd-decorated ZnO nanorod arrays which is *in-situ* synthesized on ceramic tubes by a simple wet-chemical method. Compared to pure ZnO nanorods, Pd-decorated ZnO nanorods sensor exhibited better sensing properties in the lower operating temperature region with a large response of 5.5 to 5 ppm trimethylamine. What’s more, photoactivation can also effectively improve metal oxide semiconductors gas sensing performance owing to the generation of photogenerated carrier favors the creation of reactive oxygen species at low temperature and promotes chemisorption photocatalytic oxidation reactions. Chen et al. [138] synthesized oxygen vacancy-enriched Pd-decorated ZnO (OV Pd/ZnO) nanorods by heating the solution-fabricated ZnO nanorods at 450 °C in H_2_ atmosphere for one hour. The sensors based on OV Pd/ZnO and Pd/ZnO both showed low responses of 5.4% and 1.1% to 0.1% CH_4_, respectively. As presented in Fig. 10a, under visible-light illumination, OV Pd/ZnO exhibited an ultrahigh response of 36.8% at 80 °C, while ZnO sensor showed no response and Pd/ZnO sensor showed a weak response of 2.3%. The temperature-programmed desorption of chemisorbed oxygen results revealed that the synergetic effect of various surface chemisorbed oxygen species, Pd NPs, and visible light illumination played a decisive role in forming active chemisorbed oxygen species on the surface of ZnO, which could be attributed to the improved sensing properties. Beyond that, Luo et al. [139] demonstrated a method to further enhance the selectivity of the sensor. They fabricated a core–shell nanostructured ZnO/Pd@ZIF-8 through self-templating process. Under visible light illumination, the ternary compound showed a response of 16.9% to 0.1% CH_4_ at 80 °C and successfully excluded the interference of NO_2_, as the kinetic molecule (4.5 Å) of NO_2_ is larger than the aperture size (4.0–4.2 Å) of ZIF-8, while the diffusion of CH_4_ (3.8 Å) is less influenced. Chen et al. [140] presented Pd-decorated ZnO nanowires for NO_2_ sensor via one-pot hydrothermal process. The Pd NPs were self-assembled on the surface of ZnO nanowires, helping to lower the operating temperature, increase the response value, and enhance the selectivity towards NO_2_, as shown in Fig. 10b. Lupan et al. [141] displayed a RT H_2_ sensor based on Pd/ZnO nanowires synthesized via one-step electrochemical deposition, and the schematic illustration of the nano-sensor is shown in Fig. 10c. The sensitivity of gas sensors increases as the grain size of metal oxide decreases [142, 143]. The authors also derived the response value of nanowires as a function of diameter, indicating that nanowires with smaller diameters typically have greater sensitivity. Their theory is also confirmed by the experimental results in Fig. 10d. Furthermore, the sensor exhibited high-performance sensing properties with response value of 13,100, response/recovery time of 6.4/7.4 s to 100 ppm H_2_ at RT. The low current values (pA-nA), ultralow power consumption, and great long-term stability (> 30 days) also demonstrate the prospect of handheld instruments. Uddin et al. [144] fabricated a H_2_ sensor based on Pd-modified ZnO nanorods arrays for monitoring dissolved H_2_ in transformer oil, as shown in Fig. 10e. The Pd catalysts modified on ZnO nanorods not only enhance the sensing performance of H_2_ at RT, but also protect the ZnO nanorods in liquid oil, which makes the prepared devices have long-term stability. The prepared sensor exhibited a large response in the transformer oil working environment for low concentrations (5–100 ppm) of dissolved H_2_ at oil temperatures of 40–80 °C, rendering it a potential candidate for transformer oil applications. Rashid et al. [145] reported a flexible RT H_2_ sensor based on Pd/ZnO nanorods on polyimide (PI) through aqueous solution and magnetron sputtering process. The gas sensor exhibited fabulous sensing properties with a great response of 91% to 1000 ppm H_2_, response time of 18.8 s, and LOD of 0.2 ppm. What’s more, as presented in Fig. 10f the sensor showed no degradation even under 90° bend and maintained high performance after 10^5^ bending/relaxing cycles, showing excellent flexibility properties.

Besides, Pd-decorated 2D ZnO nanomaterials have also attracted the interest of researchers due to their small thickness and high specific surface area, which are ideal for the enhanced surface reactions and detections of target gas. Kim et al. [146] prepared 2D ZnO nanosheets with a thickness of approximately 1 nm via facile hydrothermal process and modified with Pd through UV radiation. The Pd-modified nanosheet successfully detects H_2_ at the concentration down to 0.1 ppm with excellent selectivity. In addition, the Pd/ZnO nanosheets were further prepared on PI substrates and tested by bending, tilting and stretching to demonstrate the great mechanical flexibility. Xiao et al. [147] synthesized high-performance acetone sensors based on the Pd-modified porous single-crystal ZnO nanosheets. The ZnO nanosheets was produced by solvothermal method, while Pd NPs were self-assembled on their surface. The authors contributed the fabulous sensing properties to the Pd modification effect and the high percentage of the single-crystal ZnO nanosheets encased in (100) facets. Furthermore, they proposed that different 2D and 3D ZnO nanocrystals encapsulated in high index facets could greatly enhance the sensing performance of chemical sensors. Beyond that, a variety of interesting morphologies based on ZnO nanosheet have also been prepared for high-performance gas sensors. Zhang et al. [148] prepared Pd-modified ultrathin agaric-like ZnO nanosheets aniline sensors by facile hydrothermal process. As shown in Fig. 10g, the sample exhibited superior selectivity and sensitivity to aniline, which is approximately two orders larger than pristine ZnO nanosheets. Beyond that, the aniline sensor has a low detection limit of 0.5 ppm with a response/recovery time of 29/23 s. Hung et al. [149] synthesized porous coral-like ZnO nanosheets by hydrothermal method and modified them by direct reduction of Pd ions, as shown in Fig. 10h. With Pd modification, the optimal operating temperature was significantly reduced from 450 to 350 °C, with a threefold improved response to acetone, about threefold faster response/recovery time, and a very low theoretical detection limit of 17 ppt compared to the pristine ZnO nanosheets sensor. In addition to the modification of Pd NPs on the ZnO surface, a H_2_ sensor based on hydrothermally synthesized Pd@ZnO core–shell NPs was reported by Nguyen et al. [150] The high Barrett-Emmett-Teller (BET) specific surface area of the core–shell material also provides a large number of active sites for accelerating the sensing reaction, which is beneficial for enhancing the sensing performance, as illustrated in Fig. 10i. Secondly, they also found that the sensing performance of the core–shell samples calcined in argon was superior to that in air, attributed to the high content of metal Pd^0^ species.

#### ***Pd-Decorated SnO***_*2*_*** Gas Sensors***

**Fig. 11:**
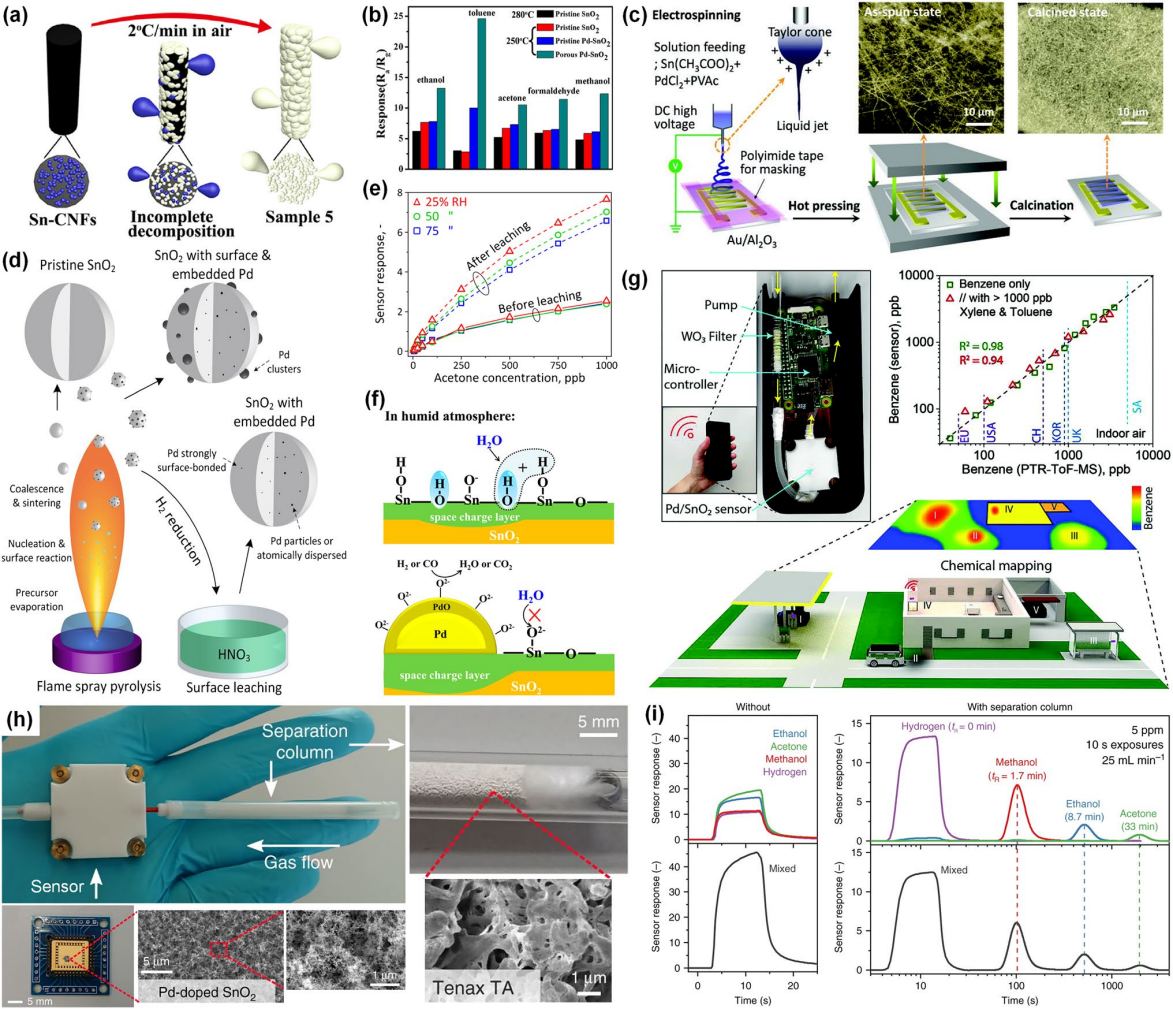
**a** Schematic illustration of the fabrication process for carbonized Pd-SnO_2_ nanofiber. **b** The response and selectivity of the sensors based on pristine SnO_2_ nanofiber, pristine Pd-SnO_2_ nanofiber, carbonized Pd-SnO_2_ nanofiber. Reproduced with permission from Ref. [152]. Copyright 2018, Elsevier. **c** Schematic illustration of the fabrication process for Pd-loaded SnO_2_ nanofiber mats. Reproduced with permission from Ref. [153]. Copyright 2010, Wiley–VCH. **d** Schematic illustration of the fabrication process for Pd-embedded SnO_2_ NPs. **e** The response to 0–1000 ppb acetone under different humidity conditions from Pd-embedded SnO_2_ NPs sensors before and after leaching. Reproduced with permission from Ref. [158]. Copyright 2020, Springer Nature. **f** Schematic diagram of gas sensing mechanism for pristine SnO_2_ and Pd-decorated SnO_2_ in humid condition. Reproduced with permission from Ref. [162]. Copyright 2015, American Chemical Society. **g** Handheld device for indoor benzene sensing and envisioned chemical mapping application. Reproduced with permission from Ref. [163]. Copyright 2021, Wiley–VCH. **h** Images of the handheld methanol sensor comprised of a flame-made Pd-SnO_2_ NPs microsensor and an upstream separation column filled with Tenax TA particles. **i** Single and mixed dynamic response curves of the Pd-SnO_2_ NPs sensor connected without (left) and with (right) the separation column. Reproduced with permission from Ref. [164]. Copyright 2019, Springer Nature

To date, a variety of synthetic methods have been reported for the preparation of Pd-decorated SnO_2_ for gas sensing. Firstly, as we all know, electrospinning is a kind of one-step synthesis technique with great properties such as simple process, versatility, low cost, large-scale preparation, and the ability to produce micron to nanometer fibers. Metal oxides prepared by electrostatic spinning not only have a 1D nanostructure induced by the preparation process, but also have a high surface-to-volume ratio and porosity owing to the interconnection between NPs. Various morphologies consisting of different pores and discontinuous segments can be obtained by adjusting the key factors of the electrospinning method, such as flow rate and volatile solvent. Teng et al. [151] prepared mesoporous PdO-decorated SnO_2_ nanotubes by one-step electrospinning technique. The fabricated sensors exhibited high response of 20.3 with a rapid response time of 1.33 s to 100 ppm of NO_2_ at RT and reached a low detection limit of 10 ppb. The fabulous sensing properties can be attributed to the SnO_2_ tubular nanostructure and well-dispersed mesopores that provide abundant channels for gas diffusion and adsorption, and oxygen defects and chemisorbed oxygen act as active sites for increasing rates of electron transport due to the accelerated electron capture and charge transfer. In addition, Xie et al. [152] fabricated SnO_2_ nanofibers by electrospinning and formed porous nanostructure by subsequent carbonization treatment by calcination in air, as presented in Fig. 11a. The carbonization treatment improved the response of Pd/SnO_2_ nanofiber sensors to all gases without sacrificing the selectivity, as shown in Fig. 11b, which could be ascribed to the formation of the hollow nanostructure, enhancement in surface area, and increased induced chemisorbed oxygen. Yang et al. [153] synthesized Pd-modified SnO_2_ nanofiber mats by electrospinning, hot pressing, and calcination, as presented in Fig. 11c. The Pd loading suppress grain growth and densification, resulting in smaller SnO_2_ nanofiber grains and higher surface area. Beyond that, compared with the pristine SnO_2_ sensors, the Pd-decorated SnO_2_ sensors have a large resistivity of 4 orders of magnitude and exhibited an increased response to H_2_ and a decreased response to NO_2_, which can effectively improve the selectivity of the sensors.

In addition to electrospinning, GLAD, solvothermal, and vapor–liquid-solid process have also been reported for the preparation of SnO_2_ 1D nanostructures with high specific surface area. Jung et al. [154] used GLAD method to prepare SnO_2_ nanorods with an average height and diameter of 200 and 30 nm, respectively. Subsequent loading with 5 nm thin layers of metal catalysts such as Au, Pt, or Pd, all of them improving the sensing performance of the SnO_2_ nanorods sensors. However, the Pd-modified SnO_2_ sensor showed the best detection performance for C_2_H_2_ with an ultralow detection limit of 0.01 ppm. Lu et al. [155] synthesized colloidal SnO_2_ nanowires with an ultrasmall diameter of ~ 2 nm through a simple solvothermal process. Furthermore, the Pd-modification of the SnO_2_ nanowires by employing the PdCl_2_ surface ligand replacement technique effectively reduce the sensor operating temperature from 250 to 150 °C and decrease the response/recovery time to 6/3 s. Cai et al. [156] prepared SnO_2_ nanowires by the vapor–liquid-solid process and modified Pd NPs on the surface of SnO_2_ by UV irradiation of PdCl_2_ solution. With the variation of the quantity of Pd NPs, the Pd-modified SnO_2_ nanowires exhibit different H_2_ sensing responses, which can be well controlled by adjusting the irradiation time. What’s more, SnO_2_ nanowires decorated with optimal amounts of Pd NPs showed a 12.7-fold higher response than bare SnO_2_ nanowires when exposed to 100 ppm H_2_. Choi et al. [157] similarly prepared SnO_2_ nanowires using a facile vapor–liquid-solid process and then achieved Pd-embedded SnO_2−x_ modification via flame CVD. The response of the Pd-embedded SnO_2−x_ gas sensor at 200 °C was 21.87, 2.23, 1.69 and 1.51 for NO_2_, ethanol, benzene, and H_2_ gases at 10 ppm, respectively, reflecting its excellent selectivity for NO_2_ gas, especially excluding the effect of H_2_. Pineau et al. [158] fabricated SnO_2_ particles with different Pd contents by flame spray pyrolysis and treated with nitric acid, leaching the Pd particles that were exposed to the SnO_2_ surface to obtain Pd-embedded SnO_2_ particles, as shown in Fig. 11d. In addition, the Pd-embedded SnO_2_ sensors exhibited a higher response to acetone than the Pd-decorated SnO_2_ particles without leaching and demonstrated excellent resistance to humidity, as presented in Fig. 11e. This points out that small amounts of precious metals embedded in metal oxides may be more effective than on the surfaces.

Furthermore, there are many methods to synthesize porous nanospheres, such as the simple hydrothermal process, template-sacrificial method, facile precipitation method, etc. Porous nanospheres of SnO_2_ have also been proved to have high gas response and fast gas responding kinetics due to the large specific surface area that provides more contact area for gas molecules and helps taking full advantage of the catalytic effect of Pd NPs. Duan et al. [159] synthesized Pd-modified SnO_2_ porous spherical composites by simple hydrothermal method. The sensors exhibited a significant response of 18.1 to 200 ppm H_2_ at 330 °C under UV radiation. Under UV radiation, a certain amount of photogenerated electron–hole pairs are excited on the surface of SMOs. The photogenerated electron–hole pairs are less bound to atoms than those generated during the redox reaction between SMOs and the target gas, which facilitates a faster response speed and a larger response value. In addition, the hollow nanospheres provide a larger gas contact area, which can also be effective in improving sensor characteristics such as sensitivity. Cai et al. [160] synthesized porous SnO_2_ hollow nanospheres with the sacrifice of carbon nanospheres and decorated Pd NPs by UV irradiation to enhance their sensing properties. The Pd-modified SnO_2_ hollow spheres sensor exhibited a ultrahigh response of 121 to 100 ppm H_2_ at 200 °C, while the response of the bare SnO_2_ hollow spheres sensor was only 3.1. Beyond that, after Pd modification, the selectivity for H_2_ was significantly improved, compared to reducing gases including ethanol, acetone, p-xylene, toluene, and benzene. Suematsu et al. [161] formed clustered SnO_2_ NPs (~ 45 nm) by aggregating monodispersed nanocrystals (~ 5 nm) prepared through hydrothermal method. The clustered NPs have particularly high porosity owing to the loose accumulation of large particles, which improves the gas diffusivity of the sensing membrane. In particular, the clusters of Pd-decorated SnO_2_ NPs not only showed fabulous response to 200 ppm H_2_ (S = 2,020), 200 ppm CO (S > 520), and 50 ppm toluene (S = 1,720), but also exhibited ppb-level detection capability. Moreover, Pd decoration have also been reported to play a significant role in the enhancement of humidity resistance properties of the sensors. Ma et al. [162] prepared Pd-decorated SnO_2_ NPs and illustrated the oxygen adsorption behavior and sensing performance for H_2_ and CO under different humidity conditions. As illustrated in Fig. 11f, they proposed that in a humid atmosphere, the adsorbed oxygen species on the pure SnO_2_ surface was mainly O^−^, and the Pd loading provided initial adsorption sites for the adsorption of O^2−^, which is not easily affected by water vapor, thus reducing the effect of water vapor on the conductivity and sensor response, and greatly enhancing the sensitivity of the sensor in the humid atmosphere. Weber et al. [163] fabricated a handheld benzene sensing device based on Pd/SnO_2_ NPs prepared by flame spray pyrolysis. Meanwhile, they designed WO_3_ nanoparticle catalytic filters to selectively removed the interference of disturbing gases such as challenging toluene and xylene, exhibiting an ultrahigh selectivity. Beyond that, ultralow concentrations (13 ppb) of benzene can be detected at relative humidity of 10 ~ 80% with superior stability. Furthermore, as presented in Fig. 11g, the device was also successfully used to quantify the amount of benzene in spiked indoor air and transmits the data wirelessly to a terminal for analysis. Actually, Broek et al. [164] have already reported a similar inexpensive and handheld methanol gas sensor comprised of a flame-made Pd-SnO_2_ NPs microsensor and an upstream separation column filled with Tenax TA particles (Fig. 11h). The difference lies in the type of the selected filter. Since methanol and ethanol are chemically similar molecules, choosing the separation column as the filter is expected to acquire much better separation results. More specifically, the separation column can effectively separate methanol from ethanol and other interfering analytes like H_2_ and acetone, contributing to the expected selective detection of methanol visually displayed in Fig. 11i. Notably, based on such an attractive handheld sensor with remarkable selectivity, further practical applications in the discrimination of methanol adulteration in alcoholic beverages [165] and the noninvasive diagnosis for methanol poisoning [166] were thoroughly demonstrated in their follow-up works. In addition, Broek et al. [167] also fabricated a portable formaldehyde sensor with a high selectivity for indoor air monitoring with the same filter-sensor structure consisting with a Tenax TA powder-based separation column and a flame-made Pd-SnO_2_ NPs microsensor.

#### ***Pd-Decorated WO***_*3*_*** Gas Sensors***

In recent years, Pd-decorated WO_3_ sensors have been reported to detect a wide range of gases, including H_2_, CO, NH_3_, H_2_S, acetone, toluene, and so on.

**Fig. 12:**
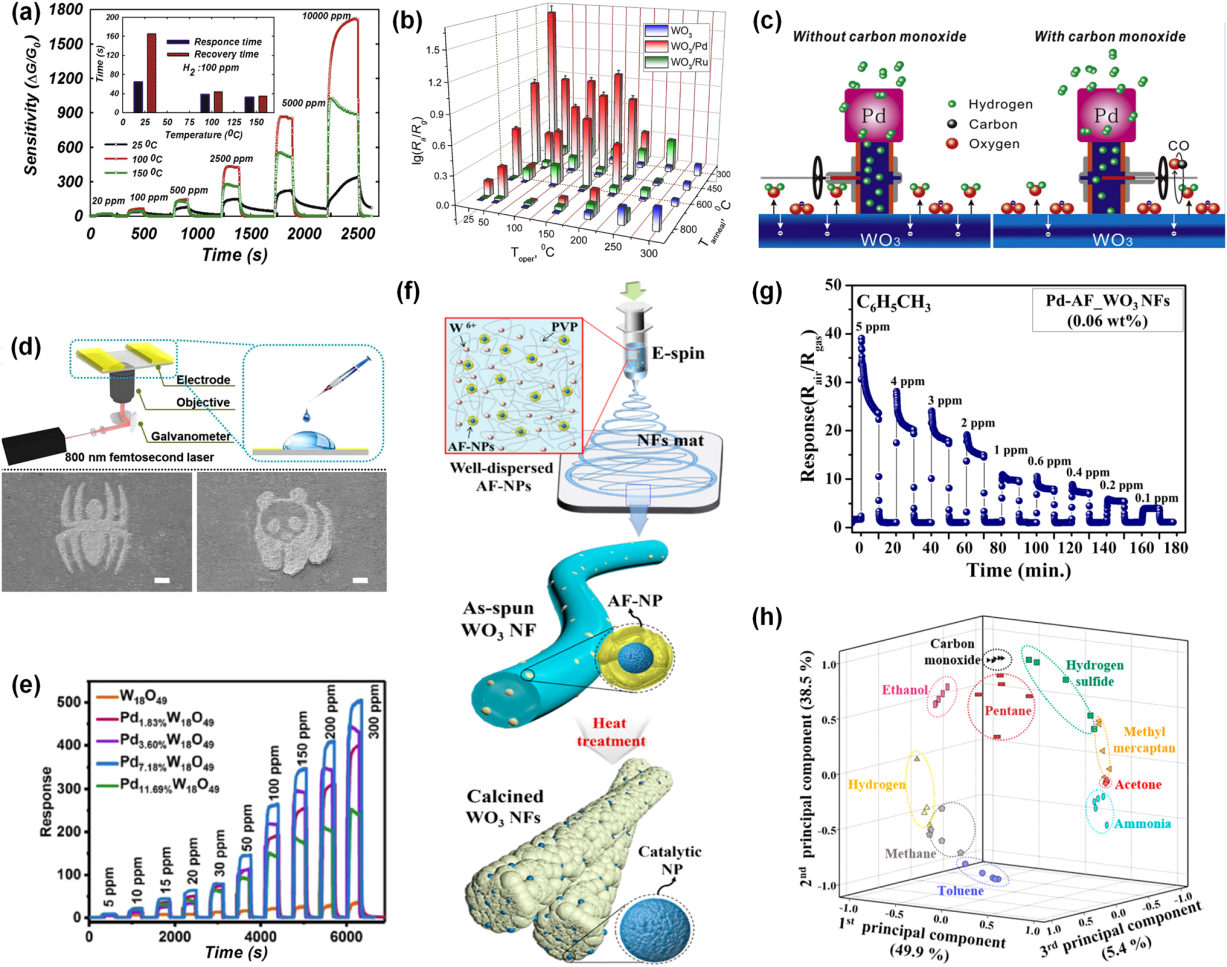
**a** Transient response of Pd-WO_3_/PRGO sensors to different concentrations of H_2_. Reproduced with permission from Ref. [169]. Copyright 2014, Elsevier. **b** The sensitivity of pure and modified WO_3_ under different annealing temperature and operating temperature. Reproduced with permission from Ref. [171]. Copyright 2018, Elsevier. **c** Schematic diagram of the effect of CO molecular valve. Reproduced with permission from Ref. [172]. Copyright 2018, Elsevier. **d** Schematic diagram of sensor fabrication through femtosecond laser direct-writing technique and the SEM images of the prepared programmable patterns. Reproduced with permission from Ref. [174]. Copyright 2020, Elsevier. **e** Transient response of Pd_x_W_18_O_49_ sensors to different concentrations of acetone. Reproduced with permission from Ref. [47]. Copyright 2021, Elsevier. **f** Schematic diagram of the electrospinning process for mesoporous WO_3_ nanofibers modified with apoferritin-encapsulated proteins catalyzed noble metal NPs. **g** Transient response of Pd-modified WO_3_ nanofibers sensors to different concentrations of toluene. **h** 3D feature space of PCA using the fabricated WO_3_ sensors with Pd, Pt and Rh modifications for different concentrations of 10 exhaled biomarker gases. Reproduced with permission from Ref. [176]. Copyright 2016, American Chemical Society

Han et al. [168] successfully fabricated multilayer porous Pd-WO_3_ nanocomposite films via layer-by-layer deposition based on sol–gel process. The prepared sensor exhibited a fast response time of 7 s and an ultrahigh sensitivity (S = 956.5) to 1000 ppm H_2_, which is 346.5 times larger than that of the pristine WO_3_ film. In addition, Esfandiar et al. [169] utilized a controlled hydrothermal process to incorporate ribbon-like Pd-WO_3_ onto partially reduced graphene oxide (PRGO) sheets to form layered Pd-WO_3_/PRGO composite with high surface area. PRGO can not only significantly improve the conductivity of the composite, but also increase the porosity of the composite for more active sites and faster adsorption/desorption. As shown in Fig. 12a, the Pd-WO_3_/PRGO sensor can detect a wide range (20 ~ 10,000 ppm) of H_2_ at low temperatures and showed large response values with fast response/recovery time. Zhou et al. [170] developed a 3D urchin-like hierarchical nanostructure of Pd-W_18_O_49_ assembled from nanorods. The novel nanostructure with large surface area, the abundance of oxygen vacancies in W_18_O_49_, and the catalytic activity of Pd NPs lead to the excellent H_2_ sensing performance of the Pd-W_18_O_49_ sensor, including high response values of 1,600 to 0.1 vol% H_2_ at 100 °C and fast response/recovery time of 60/4 s.

Pd-modified WO_3_ was also used for direct detection and model inference of CO concentrations. Marikutsaa et al. [171] prepared WO_3_ NPs with variable particle size and surface area by aqueous deposition and thermal treatment, and Pd decoration was further carried out by impregnation to improve the sensitivity to CO. As shown in Fig. 12b, without the addition of Pd, the WO_3_ did not interact with CO at RT. While after Pd decoration, the sensors exhibited better working performance at lower operating temperatures, which could be attributed to the strong binding of CO to the Pd sites and the oxidation of CO with surface aqueous species. In addition, the CO concentration can be inferred from the H_2_ sensing performance under CO interference. This provides a novel method for CO detection based on tunable interfacial effects. Xiao et al. [172] displayed Pd-modified WO_3_ sensors that were sensitive to H_2_ but not to CO. Moreover, the presence of CO inhibited the absorption and desorption of H_2_ by Pd NPs. As shown in the Fig. 12c, in the air atmosphere, hydrogen atoms can easily spill over from the Pd NPs to the surface of WO_3_ and react with the chemisorbed oxygen to release electrons. However, in the presence of CO, the hydrogen spillover is blocked, resulting in a reduction of the amount of released electrons, which acts as a molecular valve effect. By modelling the theory of the molecular valve, the CO concentration can be calculated from the H_2_ sensing response in the presence of CO interference, demonstrating the possibility of single sensor to sensing multiple gases.

Other reducing gases such as H_2_S, NH_3_, etc. have also been reported to be detected by Pd-modified WO_3_. Wang et al. [173] synthesized WO_3_ microspheres by solvothermal process, followed by Pd modification through simple impregnation. The sensitivity, response speed, and selectivity were significantly enhanced after Pd loading. The response to 25 ppm H_2_S reached 1029, which was 270 times larger than that of the pure WO_3_, along with outstanding resistance to humidity and short response time of 1 s. Dai et al. [174] innovatively employed a high-precision femtosecond laser direct-writing technique to print semiconductor metal oxide NPs into mask-free microscale programmable patterns, as shown in Fig. 12d. They first prepared Pd-WO_3_∙xH_2_O NPs by simple hydrothermal method, and then fabricated single microwires made of assembled NPs by laser direct writing, which can detect as low as 1 ppm NH_3_ at RT with high response, fast response/recovery, and great selectivity. This programmable femtosecond laser-induced deposition method can not only deposit various sensing metal oxide, such as ZnO, CuO, and MoO_3_, but also pattern into various shapes such as snake-shape and other stretchable geometries, which provided numerous possibilities for multifunctional sensors based on various sensing nanomaterials, such as configuration of stretchable functions and optoelectronic sensing.

Pd-modified WO_3_ has also been reported for the detection of various VOCs. Kim et al. [175] synthesized porous Pd-embedded WO_3_ nanofibers through one-step electrospinning and further decorated Pd NPs on the surface by polyol process. The Pd-NPs/Pd-embedded WO_3_ nanofibers with both inner and outer layers modified by Pd catalysts showed a large toluene response (S = 5.5 to 1 ppm) and selectivity for H_2_S (S = 1.36 to 1 ppm), while the pristine WO_3_ nanofibers showed a high response to H_2_S (S = 11.1 to 1 ppm) and low response to toluene (S = 1.27 to 1 ppm), completely changed the sensing properties of WO_3_ nanofibers. Moreover, the combination of highly porous WO_3_ nanofibers with novel nanostructures featuring internally and externally modified Pd NPs attributed to low detection limit of 20 ppb, which is feasible for lung cancer diagnosis. Moreover, Pd-decorated W_18_O_49_ was also used to fabricated acetone sensors. Li et al. [47] prepared Pd_x_W_18_O_49_ nanowires by a simple hydrothermal method and analyzed the effect of Pd content on oxygen vacancies. With the increase of Pd content, the oxygen vacancy concentration gradually increased and then decreased. The oxygen vacancy concentration reached maximum at the Pd content of 7.18%, and the Pd_7.18%_W_18_O_49_ nanowire also exhibited best sensing performance of excellent selectivity, fast response/recovery time of 5/10 s, and a wide detection from 100 ppb to 300 ppm with large sensitivity, as presented in Fig. 12e.

In addition to preparing a single gas sensor for the detection of target gas, sensor arrays are recently being combined with machine learning techniques to detect multiple gases. Kim et al. [176] proposed an efficient catalyst loading route for detecting ppb-level target gases in exhaled breath. Noble metal NPs are embedded in polar protein nanocages composed of hollow apoferritin and the apoferritin-encapsulated proteins catalyzed NPs are further dispersed in electrospun mesoporous WO_3_ nanofibers. After calcination, the noble metal NPs were uniformly distributed in the nanofibers, and the shell layer of the apoferritin proteins effectively prevented massive aggregation of the noble metal NPs, as shown in Fig. 12f. The Pd-modified WO_3_ sensor showed superior sensing characteristics even in fully humid air and was able to detect toluene down to 0.1 ppm, as presented in Fig. 12g. Furthermore, they applied the fabricated WO_3_ sensors with Pd, Pt, and Rh modifications for PCA and successfully identified 10 exhaled biomarker gases accurately, as shown in Fig. 12h. With the combination of machine learning algorithms, multiple gases can be detected with only three sensors in this work, revealing that the innovation of sensing data processing method is of significant role in the development of electronic nose.

#### Pd-Decorated Other SMOs-Based Gas Sensors

Besides, various other semiconductor metal oxides, including In_2_O_3_, TiO_2_, Fe_2_O_3_, CuO, Co_3_O_4_, CeO_2_, MoO_3_, NiO, and V_2_O_5_ have also been reported to improve the properties of gas sensors by Pd decoration and exhibit promising gas sensing characteristics.

**Fig. 13:**
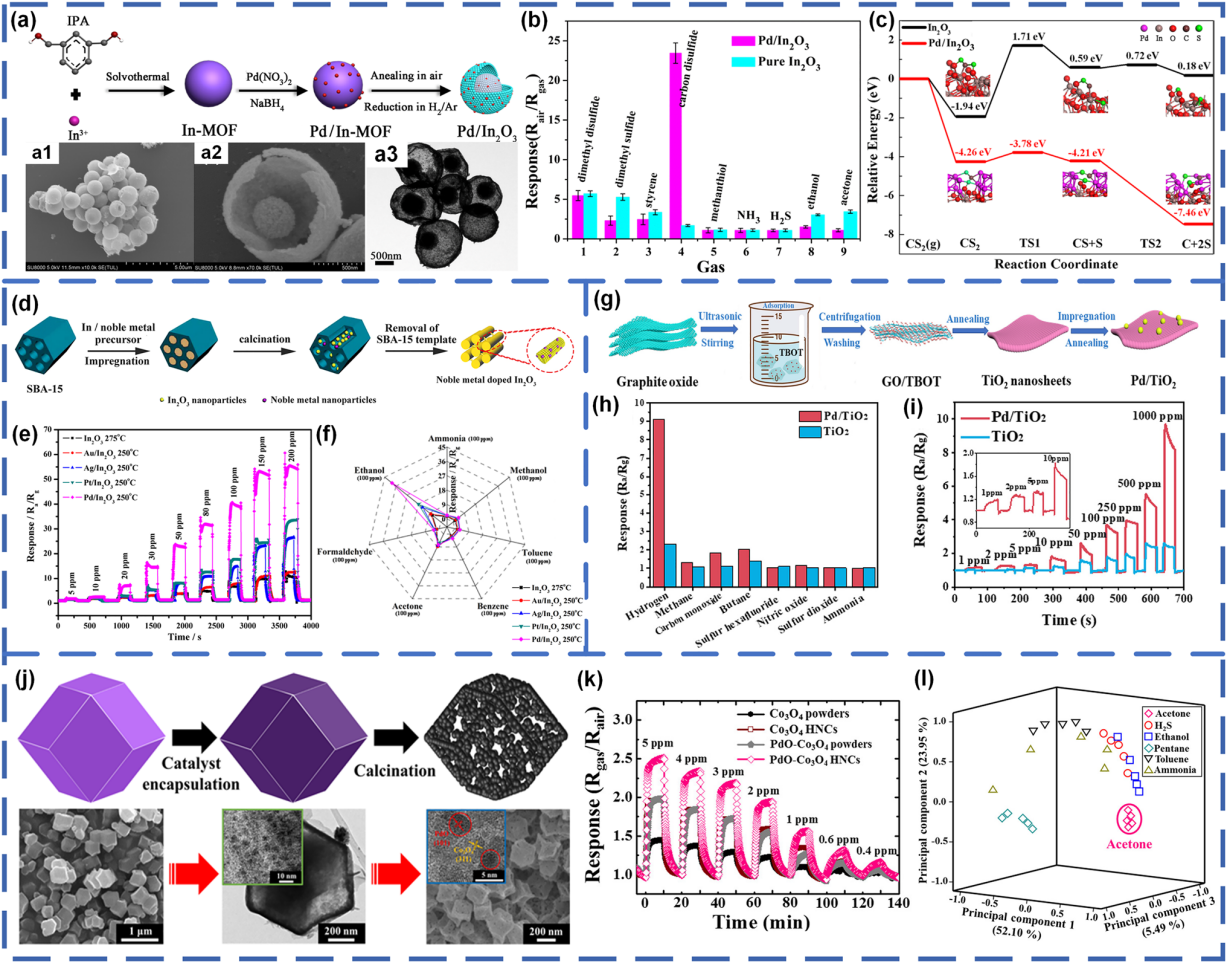
**a** Schematic illustration of the fabrication process for carbonized Pd-decorated In_2_O_3_ nanocomposites with yolk-shell nanostructure and the corresponding SEM and TEM images. **b** Sensitivity of pure and Pd-modified In_2_O_3_ for different gases at 10 ppm. **c** Calculation of the energy distribution of the reaction between the Pd/In_2_O_3_ composite and the pristine In_2_O_3_ surface in the process of CS_2_ sensing. Reproduced with permission from Ref. [179]. Copyright 2019, American Chemical Society. **d** Schematic illustration of the fabrication process for long-range ordered noble metal-doped mesoporous In_2_O_3_ with high integrity. **e** Transient response of various noble metal (Au, Ag, Pt, and Pd) doped In_2_O_3_ sensors to different concentrations of ethanol. **f** Radar diagram of various noble metal (Au, Ag, Pt, and Pd) doped In_2_O_3_ sensors to different 100 ppm gases. Reproduced with permission from Ref. [178]. Copyright 2021, IOP Publishing. **g** Schematic illustration of the fabrication process for 2D porous Pd/TiO_2_ nanosheet. **h** The sensitivity of pure and Pd-modified TiO_2_ for different gases at 1000 ppm. **i** Transient response of Pd/TiO_2_ to different concentrations of H_2_. Reproduced with permission from Ref. [185]. Copyright 2021, Elsevier. **j** Schematic illustration of the fabrication process for PdO-Co_3_O_4_ HNCs and the corresponding SEM and TEM images. **k** Dynamic response curves of Co_3_O_4_ powders, Co_3_O_4_ HNCs, PdO-Co_3_O_4_ powders, and PdO-Co_3_O_4_ HNCs towards 0.4 ~ 5.0 ppm acetone. **l** 3D feature space of PCA based on Co_3_O_4_ powders Co_3_O_4_ HNCs and PdO-Co_3_O_4_ HNCs for different concentrations of gases reproduced with permission from Ref. [194]. Copyright 2017, American Chemical Society

In_2_O_3_ has been extensively explored as a wide bandgap transparent conductive oxide in gas sensors. Pd-modified In_2_O_3_ has been reported to be extremely valuable for the wide detection of various gases, including acetone [177], ethanol [178], carbon disulfide (CS_2_) [179], NO_2_ [180], H_2_ [181], and TEA [182]. Liu et al. [179] synthesized Pd-decorated In_2_O_3_ nanocomposites with yolk-shell nanostructure by introducing MOF-templated route and annealing treatment, and the novel process preparation diagram and the corresponding SEM and TEM images are shown in Fig. 13a. The synthesized Pd/In_2_O_3_ is capable of detecting trace-level CS_2_ gas molecules. Figure 13b displayed a significantly enhanced Pd/In_2_O_3_ response and superior selectivity towards CS_2_ compared to the pristine In_2_O_3_ sensor. More significantly, to better investigate the role of the loaded Pd in the CS_2_ sensing process, quasi-*in-situ* X-ray photoelectric spectroscopy (XPS) analysis and DFT calculations were performed. Figure 13c exhibits the energy distribution of Pd/In_2_O_3_ and pure In_2_O_3_ in the process of CS_2_ sensing and concludes that Pd NPs can catalyze the desulfurization reaction and the generated intermediate S is essential to obtain high sensitivity and fabulous selectivity. Mesoporous In_2_O_3_ has been applied to prepare different morphologies of mesoporous Pd/In_2_O_3_ such as nanospheres [177], nanofibers [178], and 3D ordered macroporous structures [180]. The special mesoporous structure, with its abundant active sites, large surface area, and light weight, facilitates the interaction of gases with oxides. Cheng et al. [178] prepared long-range ordered noble metal-decorated mesoporous In_2_O_3_ with high integrity by impregnation and template-sacrificial methods to accelerate gas diffusion and expose active sites, as presented in Fig. 13d. Among various noble metal (Au, Ag, Pt, and Pd) decorated In_2_O_3_ sensors, Pd/In_2_O_3_ sensors exhibited the best sensing properties enhancement for detecting ethanol with the largest response (39.0 to 100 ppm ethanol), the shortest response/recovery time (25/9 s), and the lowest operating temperature (250 °C), as performed in Fig. 13e. Beyond that, the Pd/In_2_O_3_ sensors exhibited excellent selectivity towards different gases, as presented in Fig. 13f, which could be contributed to the facilitation of Pd^2+^ converting ethanol molecules into smaller and more active species. What’s more, Pd doping effectively limits crystal growth and has the highest content of O_ads_, resulting in a wide depletion region, which increases the initial resistance. Wang et al. [180] constructed a 3D ordered microporous Pd-decorated In_2_O_3_ using a colloidal crystal templating process and reduction precipitation method. The response of the sensor to 500 ppb NO_2_ at RT was 980, which was more than 5 times larger than pure In_2_O_3_. The combination of the enhanced number of surface defects and the concentration of electron, the excellent catalytic effect of Pd, and the great synergetic effect between Pd NPs and the In_2_O_3_ carrier led to the superior performance.

Pd-decorated TiO_2_ hybrids are also one of the most attractive chemiresistive gas sensing materials owing to their non-toxicity, low cost, and great stability in harsh environments [183, 184]. Wang et al. [185] proposed a 2D porous TiO_2_ nanosheet with Pd nanocrystal sensitization by graphene oxide template method and impregnation process, and found that TiO_2_ nanosheets could almost exactly replicate the dimensions of the graphene oxide template, as shown in Fig. 13g. The Pd/TiO_2_ sensor exhibited a unique selectivity for H_2_, which could be ascribed to the Pd modification, as shown in Fig. 13h. In addition, as performed in Fig. 13i, the sensor also has a transient and efficient detection performance, with rapid response/recovery time of 1.6/1.4 s, low limit of detection (1 ppm) and excellent long-time stability (> 100 days). The unique sensing properties is ascribed to the synergy of 2D ultra-thin porous nanosheets structural properties, Pd sensitization, and the superior adsorption properties. Mao et al. [186] presented nanoporous Pd/TiO_2_ composite membranes for H_2_ sensors. It was observed that the Pd decoration resulted in significant needle-like jumps in the resistance sensing curve, while this phenomenon did not occur with pure TiO_2_. They suggest that the phenomenon is the consequence of competition between increased resistance of Pd and decreased resistance of TiO_2_. Pd has the ability to reversibly adsorb and desorb large amounts of H_2_ (about 900 times its own volume). When first exposed to H_2_, the resistance of the entire material increases rapidly. And the subsequent reaction of adsorbed oxygen injects electrons into the TiO_2_ layer, resulting in a decrease in resistance and a needle-like jump in resistance sensing curve.

Iron oxide is also a traditional cost-effective gas sensing material and Pd-doped iron oxide can also further enhance the sensing performance of pristine iron oxide chemiresistive gas sensor. Yang et al. [187] prepared Pd nanoparticle-encapsulated α-Fe_2_O_3_ nanofibers by a simple electrospinning and calcination treatment. The optimal content of Pd NPs can not only absorb abundant oxygen molecules to dissociate into adsorbed oxygen ions by spill-over effect, but also activated the reaction between acetone and adsorbed oxygen ions by reducing the activation energy. Chemical sensitization of Pd NPs and the formation of p-n heterojunctions of Pd@α-Fe_2_O_3_ nanofibers resulted in enhanced sensors properties with higher response (16.6 to 100 ppm acetone), faster response/recovery speed (4/4 s), low detection of limit (50 ppb), better selectivity, and long-term stability (42 days). Sharma et al. [188] successfully synthesized Pd-loaded Fe_3_O_4_ halloysite nanotubes nanohybrids. The response of the hybrids to H_2_ exhibited a peculiar concentration-dependent n-p-n variation. An n-type response was observed at H_2_ concentrations of 250 ppb, an immediate p-type response at concentrations of 500 ppb, and a return to an n-type response at 1–5 ppm. This unusual sensing property can be ascribed to the formation of n-Fe_3_O_4_/p-Fe–O-Pd/n-Fe_3_O_4_ heterojunction structures. However, this phenomenon was not noticed in other Fe_3_O_4_ gas sensors and was probably attributed to the unique nanostructure. Although this phenomenon is rare, it contributes to a better understanding of the sensing mechanism of Pd-decorated metal oxide semiconductors.

CeO_2_ is an n-type semiconductor material with excellent oxygen storage/release capacity and a low redox potential between Ce^3+^ and Ce^4+^, leading to it being considered as a promising material for gas sensing in recent years. In contrast, pristine CeO_2_ gas sensors have an unimpressive performance due to their high band gap, low intrinsic carrier concentration, and high carrier recombination rate, which can be modified by decorating. Hu et al. [189] proposed that Pd decoration can be used to modulate the ratio of Ce^3+^ and Ce^4+^ to enhance the methanol response. The content of Ce^3+^ affects the number of oxygen vacancies, which can contribute to the absorption of surface oxygen, improving the sensing performance. With proper Pd modulation to maximize the Ce^3+^ content, the response Pd-CeO_2_ sensor is nearly four times higher than pristine CeO_2_ sensor. What’s more, Dao et al. [68] prepared Pd@n-CeO_2_ core–shell nanoplatforms for H_2_ detection. Nitrogen doping helps to increase the number of Ce^3+^ active species and oxygen vacancies, narrowing the energy bandgap and introducing more free electrons to the conduction band, thus reducing its initial resistance. In addition, Pd noble metal is an effective electron trap that extends the lifetime of thermally generated charges, and the transformed PdH_x_ injects electrons into the n-CeO_2_ shell during H_2_ detection, speeding up the response and recovery. Furthermore, the unique core–shell nanostructure results in a high BET specific surface area and greater the Barrett-Joyner-Halenda (BJH) adsorption and desorption average pore sizes, accelerating the adsorption, diffusion, and desorption processes. As a result, the sensor showed excellent sensing characteristics with great selectivity, a large response of 19 to 100 ppm H_2_, and a linear response to H_2_ concentrations from 0.5 to 100 ppm.

CuO is the most commonly used p-type metal oxide semiconductor material for chemiresistive gas sensors. However, Pd-modified CuO is rarely reported and does not perform as well compared to ZnO, SnO_2_, and WO_3_, which are typical n-type metal oxide semiconductors. Some great works with excellent gas sensing performance have been reported though. Mikami et al. [190] reported Pd-decorated Cu_2_O/CuO H_2_S sensors and proposed that Pd modification could facilitate the reaction between adsorbed oxygen and H_2_S, thus blocking the formation of Cu_2_S and improving the stability of the sensor response. However, the excessively long response time (20 min) and recovery time still need to be further improved. Nha et al. [191] prepared CuO nanoplates using a hydrothermal method and modified them with Pd NPs by directed reducing pathway at RT, and the sensors worked well even at 90% relative humidity. The Pd-decorated sensors have higher sensitivity, faster response/recovery speed compared to pure CuO sensors, although the performance is still not comparable to other reported Pd-modified n-type metal oxide semiconductors.

Co_3_O_4_ has received attention as a novel p-type gas sensing material, showing good stability and excellent performance in the evaluation of analytes such as H_2_, CO, NO, NH_3_, and VOCs [192]. Koga [193] successfully prepared aggregates of Co_3_O_4_ NPs (~ 3 nm) with uniformly dispersed Pd additives through a novel preparation route of pulsed laser ablation. And the morphology of Pd can be modified from single atoms to oxide clusters (1–2 nm). The best H_2_ sensing performance is achieved with the highest density of single Pd atoms at 5% Pd loading. Contributions of electrons to the Co_3_O_4_ valence band are provided by the decoration of a single Pd atom in the Pd^4+^ state at the Co^3+^ sites on the surface of Co_3_O_4_ NPs and leads to the higher concentration of free electrons, increasing the chance of oxygen to adsorb free electrons and form high concentration of ion-adsorbed oxygen. What’s more, the catalytic redox cycle between Pd^4+^ and Pd^2+^ facilitates the reaction between H_2_ and adsorbed oxygen during H_2_ sensing. Consequently, this Pd single-atom catalysis effectively improve sensitivity and response speed. Koo et al. [194] fabricated Pd NPs-loaded Co_3_O_4_ hollow nanocages (HNCs) with MOF templates. The porous structure of zeolitic imidazolate framework-67 (ZIF-67) acted as a sieve to limit the size of Pd NPs (2–3 nm) and enable homogeneous dispersion of Pd NPs. Subsequently, as shown in Fig. 13j, after calcination, the PdO NPs (3–4 nm) are dispersed uniformly on the walls of the Co_3_O_4_ HNCs, whose unique nanostructure can provide high specific surface area and high catalytic activity. Figure 13k displayed dynamic response curves of Co_3_O_4_ powders, Co_3_O_4_ HNCs, PdO-Co_3_O_4_ powders, and PdO-Co_3_O_4_ HNCs towards 0.4 ~ 5.0 ppm acetone. Obviously, the acetone sensing performance was markedly improved after Pd decoration, which could be attributed to the effect of nanoscale catalyst. Furthermore, a sensor array consisting of Co_3_O_4_ powders, Co_3_O_4_ HNCs, and PdO-Co_3_O_4_ HNCs was carried out. As exhibited in Fig. 13l, with the help of PCA analysis, acetone with different concentrations (1–5 ppm) were apparently discriminated from other interfering gases (H_2_S, NH_3_, ethanol, pentane, and toluene), demonstrating its potential prospects for exhaled acetone analyzers.

#### Pd-decorated Heterostructured SMOs-Based Gas Sensors

The formation of heterostructured nanomaterials is an effective strategy to improve gas sensing performance more than that of single-component SMOs. The electron transport is highly modulated by the heterojunction potential, resulting in a strong modulation of resistance when detecting target gases. In addition, the decoration of Pd metal NPs can further enhance the properties of multiple SMO heterojunction-based sensors through chemical sensitization and electronic sensitization. Dong et al. [195] reported Pd-decorated SnO_2_/In_2_O_3_ nanocomposites for butane sensing. When the nanocomposites are exposed to air, electrons in the conductive bands of SnO_2_ and In_2_O_3_ are trapped by oxygen, forming adsorbed oxygen ions, such as O^−^, and O^2−^, which leads to an increase in the electron depletion layer and in the height of the potential barrier, thus increasing the initial resistance. In this case, they found no PdO phase in the nanocomposites. With the assistance of Pd NPs, the amount of reactive adsorbed oxygen ions increases significantly due to the easy adsorption of oxygen molecules and the rapid conversion of oxygen molecules. Moreover, the activation of Pd NPs catalyzes and strengthens the oxidation of butane molecules on the SnO_2_ and In_2_O_3_ surfaces through the spillover effect. Consequently, the as-prepared nanocomposites exhibited broad detection range (1–3000 ppm), rapid response/recovery time (3.5/7.9 s), high response value (71.3 to 3000 ppm butane), and great selectivity towards common flammable gases. Kundu et al. [196] prepared Pd-decorated indium tin oxide powders for selective NH_3_ detection. They found that after calcined at 650 °C, Pd was converted to PdO, which exhibited faster oxygen chemisorption than Pd^0^, favoring increased sensitivity. In addition, the Pd modification contributed to an enhancement in sensing performance, with a significant reduction in the lower detection limit from 30 to 3 ppm, an increased sensitivity, excellent long-term stability (90 days), and negligible cross response.

**Fig. 14:**
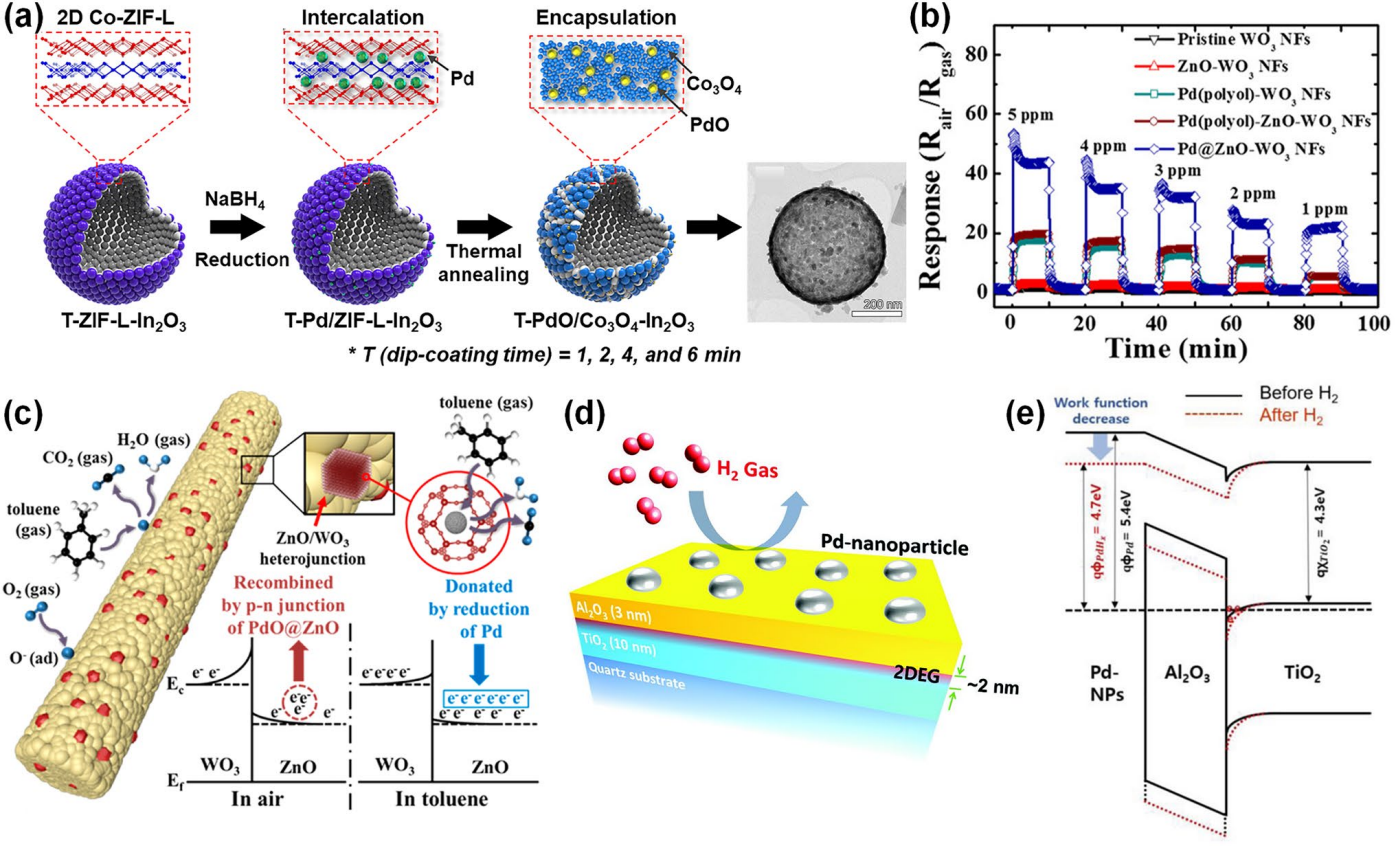
**a** Schematic illustration of the fabrication process for 2D MOF derived PdO/Co_3_O_4_-In_2_O_3_ materials and its TEM image. Reproduced with permission from Ref. [197]. Copyright 2020, Elsevier. **b** Transient response of Pd@ZnO-WO_3_ sensors to different concentrations of toluene. **c** Schematic illustration of the H_2_ sensing mechanism of Pd@ZnO-WO_3_ sensors. Reproduced with permission from Ref. [48]. Copyright 2016, American Chemical Society. **d** Schematic illustration of Pd/Al_2_O_3_/TiO_2_ sensors. **e** Energy band schematic diagram of Pd/Al_2_O_3_/TiO_2_ sensors with and without H_2_ exposure. Reproduced with permission from Ref. [198]. Copyright 2018, Wiley–VCH

Recently, SMOs derived from MOFs have exhibited promising prospects in gas sensing area due to their unique characteristics such as extraordinarily high specific surface area, ultrahigh porosity, and various nanostructures. In particular, MOFs are capable to encapsulate noble metal NPs within their interlayers or cavities, which can be easily converted into the corresponding noble metal decorated-SMOs by facile calcination. This preparation process is also reported for the preparation of Pd-decorated heterostructured SMOs-based gas sensors. Jo et al. [197] presented 2D MOFs-derived PdO/Co_3_O_4_-In_2_O_3_ sensing film for high-performance acetone sensor. Figure 14a illustrates the schematic diagram of its fabrication. In_2_O_3_ hollow spheres were first prepared by ultrasonic spray pyrolysis. Subsequently, an ultrathin cobalt zeolitic-imidazolate framework (Co-ZIF-L) nanolayer was coated onto the surface of the In_2_O_3_ hollow spheres by dip coating, followed by calcination to form Co_3_O_4_-In_2_O_3_ shell. Finally, Co_3_O_4_ nanoclusters encapsulating PdO NPs were loaded in In_2_O_3_ hollow spheres by reducing the Pd^2+^ at the intercalation layer of the ZIF-L interlayer. The incorporation of Pd in Co-ZIF-L allows the mixture of two different catalytic components at the atomic scale, thus enhancing the synergistic catalytic promotion. Furthermore, aggregation and coarsening of Co_3_O_4_ and PdO NPs during calcination is also avoided as the Co and Pd sources were restricted by the unique MOF nanostructure. As a result, the mixed-phase nanocatalysts can be uniformly dispersed on the surface of sensing film and effectively catalyze the sensing reaction. The formation of Co_3_O_4_/PdO p–n junctions and Co_3_O_4_/In_2_O_3_ n–n junctions lead to an ultrahigh response (145.9 to 5 ppm acetone) and selectivity at relatively low operating temperatures (225 °C) and high humidity (80%RH). Koo et al. [48] synthesized ZIF-8-derived Pd-loaded ZnO nanocubes nanocatalysts, loaded on as-electrospun WO_3_ nanofibers by calcination and formed Pd/ZnO and ZnO/WO_3_ dual heterogeneous multijunction. The Pd@ZnO-WO_3_ nanofibers with multiple heterojunctions exhibited large needle-like jumps toluene response (Fig. 14b), which could be contributed to the enhanced work function of Pd@ZnO, resulting in a larger initial resistance and rapid changes of resistance when exposed to toluene. In addition, as present in Fig. 14c, they found that after exposure to toluene, the oxidation state of Pd^2+^ was partially reduced to Pd^0^ state and donates electrons to ZnO, resulting in an effective modulation of the surface depletion layer. This unique MOF-derived noble metal-embedded semiconductor metal oxide multi-phase catalysts enables significant heterogeneous sensitization, facilitating optimal performance and multifunctional integration for gas sensors.

ALD is a widely employed deposition method in microelectronics, capable of controlled atomic level deposition with superior conformality, allowing the precious decoration of noble metals on SMO-based sensing materials with complex nanostructures to further improve the performance of pristine SMOs. Kim et al. [198] grew transparent, ultrathin Al_2_O_3_/TiO_2_ thin film heterostructures by ALD and formed two-dimensional electron gas (2DEG) at their interface. Subsequently, island-type Pd NPs were deposited by e-beam evaporation to form Pd/Al_2_O_3_/TiO_2_ sensors, as present in Fig. 14d, which just had a total thickness of less than 15 nm. Furthermore, adjusting the ALD process temperature modulates the 2DEG electron density on the Al_2_O_3_/TiO_2_ heterostructure, thus optimizing the sensitivity and response speed. Under the optimal process, the sensor exhibited high H_2_ sensing performance at RT, with a wide detecting range from 5 ppm to 1% and a fast response time (28 s). The sensing mechanism was also proposed, as shown in Fig. 14e. When exposed to H_2_ atmosphere, H_2_ dissociates on the surface of the Pd NPs to form PdH_x_, which reduces the work function of Pd (5.4 eV) to PdH_x_ (4.7 eV). Therefore, the energy band bending on the surface of the TiO_2_ layer increases, implicating that electrons are transferred from Pd through the very thin Al_2_O_3_ layer (3 nm) to the surface of the TiO_2_ layer and the resistance of 2DEG decreases. Unlike conventional semiconductors which use the 3D distribution of electrons to detect the target gas, the 2D system resulted in a high sensitivity. Beyond that, the authors further prepared Pd/Al_2_O_3_/TiO_2_ on flexible PI substrate. After bending 500 times, the flexible sensor still maintains reliable H_2_ sensing performance. Therefore, ultrathin nanostructures endow the ALD-grown Pd/Al_2_O_3_/TiO_2_ with fabulous flexibility properties, which are hopeful for the development of wearable gas sensing applications (Table 2).

**Table 2:** Summary of the reported Pd-decorated SMOs-based gas sensors

Materials	Structure	Synthesis method	O. T. (°C)	Target gas	Conc. (ppm)	Response	t_res_/t_rec_ (s)	LOD	Refs.
ZnO	Nanoparticles	Hydrothermal	350	H_2_	100	22^a^	1.4/7.8 min	5 ppm	[150]
ZnO	Nanorods	Self-templating	80	CH_4_	1000	16.9%^b^	3.5/4.8 min	100 ppm	[139]
ZnO	Nanosheets	Hydrothermal	250	H_2_	50	2.514^a^	336/294 s	0.1 ppm	[146]
ZnO	Coral-like nanoplates	Hydrothermal	350	Acetone	125	66.7^a^	< 15/ ~ 100 s	17 ppt	[149]
ZnO	Nanoparticles	Hydrothermal	360	H_2_	100	11.3^a^	2/5 s	5 ppm	[135]
ZnO	Nanosheets	Hydrothermal	280	Aniline	100	182^a^	29/23 s	0.5 ppm	[199]
ZnO	Nanorods	Precipitation	80	CH_4_	1000	36.8%^b^	4/4.5 min	100 ppm	[138]
ZnO	Nanorods	Wet-chemical	300	TMA	5	5.5^a^	~ 7/7 s	1 ppm	[137]
ZnO	Nanowires	Hydrothermal	100	NO_2_	1	13.5^a^	141/177 s	1 ppm	[140]
ZnO	Nanorods	CVD	260	Ethanol	500	81%^b^	6/95 s	100 ppm	[136]
ZnO	Nanowires	Electrochemical deposition	RT	H_2_	100	13100^a^	6.4/7.4 s	100 ppb	[141]
ZnO	Nanorods	Hydrothermal	40	H_2_	100	90%^b^	–	5 ppm	[144]
ZnO	Nanorods	Hydrothermal	RT	H_2_	1000	91%^b^	~ 18/184 s	0.2 ppm	[145]
ZnO	Nanosheets	Solvothermal	340	Acetone	100	70%^b^	9/6 s	10 ppm	[147]
SnO_2_	Nanoparticles	Flame Spray Pyrolysis	RT	Benzene	1	2.1^a^	36/47 s	13 ppb	[163]
SnO_2_	Nanowires	Solvothermal	150	H_2_	40	8.5^a^	6/3 s	2 ppm	[155]
SnO_2_	Hollow spheres	Hydrothermal	330	H_2_	200	14.5^a^	2.2/22.4 s	1 ppm	[159]
SnO_2_	Nanowires	Vapor–Liquid–Solid	200	NO_2_	10	21.87^a^	320/ ~ 750 s	2 ppm	[157]
SnO_2_	Nanospheres	Hydrothermal	200	H_2_	100	121^a^	34/162 s	1 ppm	[160]
SnO_2_	Nanoparticles	Flame Spray Pyrolysis	350	Acetone	1	7^a^	< 1/ < 2.3 min	5 ppb	[158]
SnO_2_	Nanorods	GLAD	200	C_2_H_2_	10	~ 0.99^a^	120 s/–	10 ppb	[154]
SnO_2_	Nanowires	Vapor–Liquid–Solid	300	H_2_	100	56^a^	22/164 s	1 ppm	[156]
SnO_2_	Nanotubes	Electrospinning	RT	NO_2_	100	~ 20.30^a^	~ 1.33/22.66 s	10 ppb	[151]
SnO_2_	Nanofibers	Electrospinning	250	Toluene	100	24.6^a^	~ 3/29 s	1.6 ppb	[152]
SnO_2_	Nanoparticles	Hydrothermal	300	Toluene	50	1720^a^	–	2.5 ppb	[161]
SnO_2_	Nanofibers	Nanofibers	350	H_2_	1	~ 3^a^	–	50 ppb	[153]
WO_3_	Nanowires	Hydrothermal	175	Acetone	300	500^a^	5/10 s	100 ppb	[200]
WO_3_	Microspheres	Hydrothermal	190	H_2_S	25	1029^a^	1 s/–	2.5 ppm	[173]
WO_3_	Nanoparticles	Sol–gel	250	H_2_	2000	~ 2000^a^	7/ ~ 299 s	50 ppm	[168]
WO_3_	Microwires	Femtosecond laser	RT	NH_3_	50	~ 1.035^a^	1.4/3.3 s	1 ppm	[174]
WO_3_	Urchin-like nanostructures	Hydrothermal	100	H_2_	1000	1600^a^	60/4 s	250 ppm	[170]
WO_3_	Nanoparticles	Hydrothermal	RT	H_2_	5000	31^a^	60 s/–	1 ppm	[172]
WO_3_	Nanocrystalline	Aqueous deposition	RT	CO	20	> 1.5^a^	4/30 min	~ 1 ppm	[171]
WO_3_	Nanofibers	Electrospinning	350	Toluene	1	11^a^	< 8.56/ < 9.2 s	< 1 ppb	[176]
WO_3_	Nanofibers	Electrospinning	350	Toluene	1	5.5^a^	10.9/16.1 s	20 ppb	[175]
WO_3_	Nanoribbon	Hydrothermal	RT	H_2_	100	38^a^	52/35 s	20 ppm	[169]
In_2_O_3_	Yolk-shell nanostructure	Calcination	135	CS_2_	50	135.3^a^	132.3/112.1 s	1 ppm	[179]
In_2_O_3_	Ordered macroporous	Precipitation	RT	NO_2_	0.5	980^a^	90/114 s	100 ppb	[180]
In_2_O_3_	Ordered mesoporous	Impregnation	250	Ethanol	100	39^a^	25/9 s	5 ppm	[178]
In_2_O_3_	Microspheres	Hydrothermal	220	TEA	50	47.56^a^	4/17 s	1 ppm	[182]
In_2_O_3_	Nanospheres	Precipitation	220	Acetone	50	~ 82^a^	6/17 s	5 ppm	[177]
In_2_O_3_	Nanoparticles	Flame spray pyrolysis	250	H_2_	150	94^a^	2/180 s	0.1 ppm	[181]
CeO_2_	Nanofibers	Electrospinning	200	Methanol	100	6.95^a^	1/5 s	5 ppm	[189]
CeO_2_	Nanoflatforms	Precipitation	350	H_2_	100	19^a^	1/6 min	0.5 ppm	[68]
CeO_2_	Nanoparticles	Hydrothermal	250	SO_2_F_2_	50	153%^b^	67/773 s	1 ppb	[201]
Co_3_O_4_	Hollow nanocages	Calcination	350	Acetone	5	2.51^a^	–	100 ppb	[194]
Co_3_O_4_	Nanoparticles	Pulsed laser ablation	125	H_2_	1000	~ 85^a^	~ 25/ ~ 80 s	1 ppm	[193]
Co_3_O_4_	Hollow polyhedral	Pyrolysis	150	Ethanol	100	~ 20.8^a^	12/25 s	10 ppm	[192]
CuO	Nanoplates	Hydrothermal	200	SO_2_	1	3.58^a^	< 53 s/–	0.5 ppm	[191]
CuO	Nanocrystals	Hot-soap	250	H_2_S	8	7.9^a^	20 min/–	1 ppm	[190]
FeO	Nanofibers	Electrospinning	220	Acetone	100	16.6^a^	4/4 s	50 ppb	[187]
FeO	Nanotubes	Reduction-precipitation	400	H_2_	100	19.8^a^	~ 6/11 s	250 ppb	[188]
TiO_2_	Nanosheet	Calcination	230	H_2_	1000	9^a^	1.6/1.4 s	1 ppm	[185]
TiO_2_	Nanoparticles	Flame stabilizing on a rotating surface	450	CO	800	5.25^a^	< 15/ < 60 s	80 ppm	[183]
TiO_2_	Thin films	Spray pyrolysis	RT	H_2_	8000	~ 1.5%^b^	4/13 s	4000 ppm	[186]
TiO_2_	Nanofiber mats	Electrospinning	180	NO_2_	2.1	38^a^	–	0.16 ppm	[184]
In_2_O_3_/ZnO	Core–shell nanoparticles	Hydrothermal	300	H_2_	100	42^a^	0.4/4.0 min	5 ppm	[202]
In_2_O_3_/Co_3_O_4_	Hollow spheres	Annealing	225	Acetone	5	145.9^a^	14 s/–	1 ppm	[197]
ITO	Nanocrystalline	Sol–gel	300	NH_3_	30	~ 82%^b^	17/36 s	3 ppm	[196]
Al_2_O_3_/TiO_2_	Thin films	ALD	100	H_2_	5	59%^b^	15/245 s	5 ppm	[198]
SnO_2_/In_2_O_3_	Nanoparticles	Solvent-thermal	320	Butane	3000	71.28^a^	3.51/7.86 s	1 ppm	[195]
ZnO/WO_3_	Nanofibers	Electrospinning	350	Toluene	1	22.22^a^	< 20 s/–	100 ppb	[48]

*O. T.* operating temperature; *Conc*. concentration; *t*_*res*_*/t*_*rec*_ response time/recovery time; *LOD* limit of detection

^a^Response is defined as R_a_/R_g_ or R_g_/R_a_, R_a_: resistance of the sensor in air, R_g_: resistance of the sensor exposed to target gas

^b^Response is defined as ∆R/R_a_ $\times$ 100% or ∆R/R_g_ $\times$ 100%, ∆R: the change in resistance, which equals to |R_a_–R_g_|

### Au-Decorated SMOs-Based Gas Sensors

Various SMO materials decorated by Au NPs, such as ZnO [203], SnO_2_ [204], WO_3_ [205], and corresponding heterogeneous structures, for gas sensors has been widely studied. Meanwhile, Au-decorated gas sensors based on different nanostructures, *e.g.,* nanoparticles [206], nanorods [207], nanowires [208], nanosheets [209], and nanoflowers [210], have been utilized to deliver superior sensing performance with high sensitivity, great selectivity, and fast response time.

#### Au-Decorated ZnO Gas Sensors

**Fig. 15:**
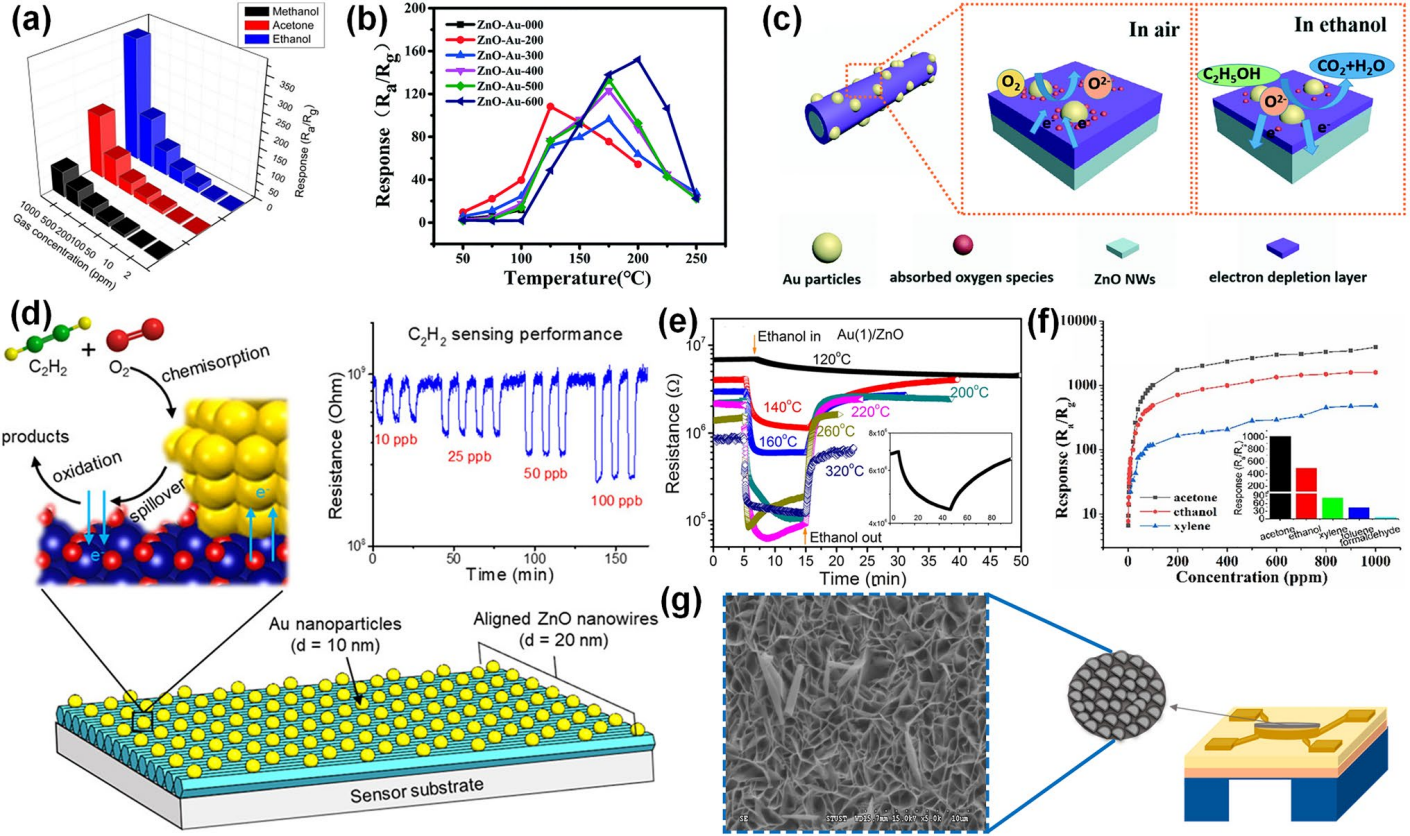
**a** Response histogram of Au/ZnO gas sensor to ethanol, methanol, and acetone with different concentrations. Reproduced with permission from Ref. [214]. Copyright 2014, Elsevier. **b** Response of Au-ZnO nanowires to 50 ppm ethanol at different operating temperatures. **c** Schematic diagram of Au NP sizes on sensing performance. Reproduced with permission from Ref. [215]. Copyright 2021, Royal Society of Chemistry. **d** Schematic diagram and transient response curve of Au@ZnO_LB towards 10–100 ppb of C_2_H_2_. Reproduced with permission from Ref. [216]. Copyright 2020, American Chemical Society. **e** Transient responses of Au/ZnO sensors towards 4769 ppm ethanol under different operating temperatures. Reproduced with permission from Ref. [217]. Copyright 2021, IOP Publishing. **f** Responses of Au doped ZnO nanosheets towards xylene, ethanol, and acetone under different concentrations, and the insert displays the selectivity to 100 ppm gases. Reproduced with permission from Ref. [209]. Copyright 2021, Elsevier. **g** SEM image of the Au–ZnO sample and the MEMS microheater. Reproduced with permission from Ref. [219]. Copyright 2020, MDPI

1D nanostructures such as nanorods [211] and nanowires [212] are believed to be able to facilitate reaction between materials and target gas molecules and thus improve the gas sensing performance of Au-decorated ZnO gas sensors [213]. Guo et al. [214] successfully fabricated Au-functionalized ZnO nanorods gas sensor for ethanol detecting. Au NPs as the sensitizer were fixed on the surface of ZnO nanorods, which enhanced the performance of the sensor with short response-recovery time and great selectivity, as shown in Fig. 15a. According to Li et al. [215], ZnO nanowires loaded with different sizes of Au NPs were successfully synthesized for ethanol detection. As is shown in Fig. 15b, the experimental results indicated that with the increase of the size of Au NPs, the gas sensitivity became worse at low temperature (≤ 125 °C). Most importantly, the Au-loaded ZnO nanowires sensor showed long-term stability and repeatability at a low concentration of ethanol. The difference of gas responses is interpreted to the Au NPs loadings with different sizes, the spillover effect of Au loads, and the formation of Schottky barrier in Fig. 15c. Furthermore, Miao et al. [216] used a Langmuir–Blodgett (LB) assembly technique to prepare a nanostructured thin film of ZnO nanowire arrays, which was then sensitized with Au NPs synthesized by sputtering and post-annealing on the substrate in Fig. 15d. The resulting Au@ZnO_LB sensor demonstrated excellent sensitivity and ppb-level sensing (3 ppb) to C_2_H_2_. It can be applied to environmental and health monitoring, biosensors, and other metal nanodot-based applications. Besides, the Au NPs were loaded on the ZnO nanorods surface through UV-assisted deposition method by Vuong et al. [217]. As presented in Fig. 15e, the ethanol sensing characteristics were tested under different working temperatures and the optimal operating temperature was 220 °C. The Au/ZnO sensor exhibited apparent enhancement towards ethanol vapor, whose response is 167 times higher than that of the pristine ZnO.

2D nanostructures have demonstrated significant potential for delivering superior gas sensing performance of Au-decorated ZnO gas sensors due to high surface-to-volume ratio [218]. Guo et al. [209] synthesized an acetone sensor based on Au NPs doped ZnO nanosheets through a hydrothermal method combined with a calcination process, which exhibited ultra-response to acetone of 1012.6 at 100 ppm and low limit of detection of 0.2 ppm, as shown in Fig. 15f. The prepared Au NPs doped ZnO is a potential material for fabricating trace determination acetone sensor used in practical medical diagnosis. Nagarjuna et al. [219] prepared the Au doped ZnO nanosheets using hydrothermal synthesis and deposited them on the MEMS device, as shown in Fig. 15g. The MEMS device microheater was prepared, which had low power consumption, small designs, and low fabrication cost. In order to better test the gas at different temperatures, the thermal performance of MEMS microheater was studied. Compared with pure ZnO nanosheets, the gas sensing response of Au doped ZnO nanosheets is increased by 15% towards 60 ppm ethanol at 300 °C. In addition, Bae et al. [203] synthesized strategic nanometric lamination of ZnO nanofilms decorated with Au NPs using ALD and thermal evaporation techniques. The Au NPs-ZnO nanofilms were optimized by the density and size control, whose response to methyl mercaptan (CH_3_SH) was 4.99% at the concentration down to 50 ppb. The prepared low detection limit sensor can selectively and accurately identify ppb-level CH_3_SH among volatile sulfur compounds, which could be applied in the early diagnosis of periodontitis.

In addition to the 1D and 2D nanostructures mentioned above, other Au-decorated ZnO nanostructures have also aroused great interest of researchers. The ethanol sensing performance of ZnO NPs decorated with different contents of Au NPs (0.5, 2, 4, and 7 wt%) were investigated by Eyvaraghi et al. [220]. What’s more, theoretical calculations were carried out using the DFT method, and the experimental results were verified. The 4 wt% Au@ZnO sensor showed about 5 times higher response than that of pure ZnO sensor as well as a reduced response time from 36 to 3 s. The theoretical calculations by DFT method revealed that the adsorption of ethanol molecules on the Au-modified ZnO surface had the best mental concentration, which was consistent with the experimental results. In addition, Wang et al. developed ZnO nanoflowers decorated by Au NPs with different Au concentrations. Experimentally, ZnO nanoflowers with 10 wt% Au NPs remarkably enhanced the acetone sensing performance of high sensitivity, short response/recovery time, and favorable selectivity. Such behaviors were benefited from the increased surface area by surface coarsening and the hybrid formation of Au/ZnO. In addition, the research confirmed that the loading concentration of the Au catalysts is significant to gas sensing characteristics. At this optimum operating temperature, when the Au content was increased from 0 to 14 wt%, the sensor displayed responses of 6.92, 28.78, 41.89, 74.41, and 35.57, respectively. The response of the ZnO decorated with 10 wt% Au was 10.75 times higher than that of the pure ZnO, which directly verified the promoting effect of Au NPs. The modulation of surface defects by electrical and chemical effects of Au NPs is regard as an important reason to improve the gas sensing performance. Meanwhile, the Schottky contacts between the Au NPs and the ZnO nanoflowers form an additional depletion layer at the interface by extracting electrons from ZnO surface defects, resulting in a decrease in electron mobility and carrier concentration. And the subsequent reaction of adsorbed oxygen with the target gases would produce stronger resistance changes and greater sensor responses. At the same time, the catalytic activity of Au NPs also made for the faster response [221].

#### ***Au-Decorated SnO***_*2*_*** Gas Sensors***

Au-decorated SnO_2_ has excellent electrical conductivity, well chemical stability, and low cost, which makes it a suitable material for gas sensors [222, 223]. The Au-decorated SnO_2_ gas sensors with numerous morphologies have attracted great interest of researchers [41, 224–226].

**Fig. 16:**
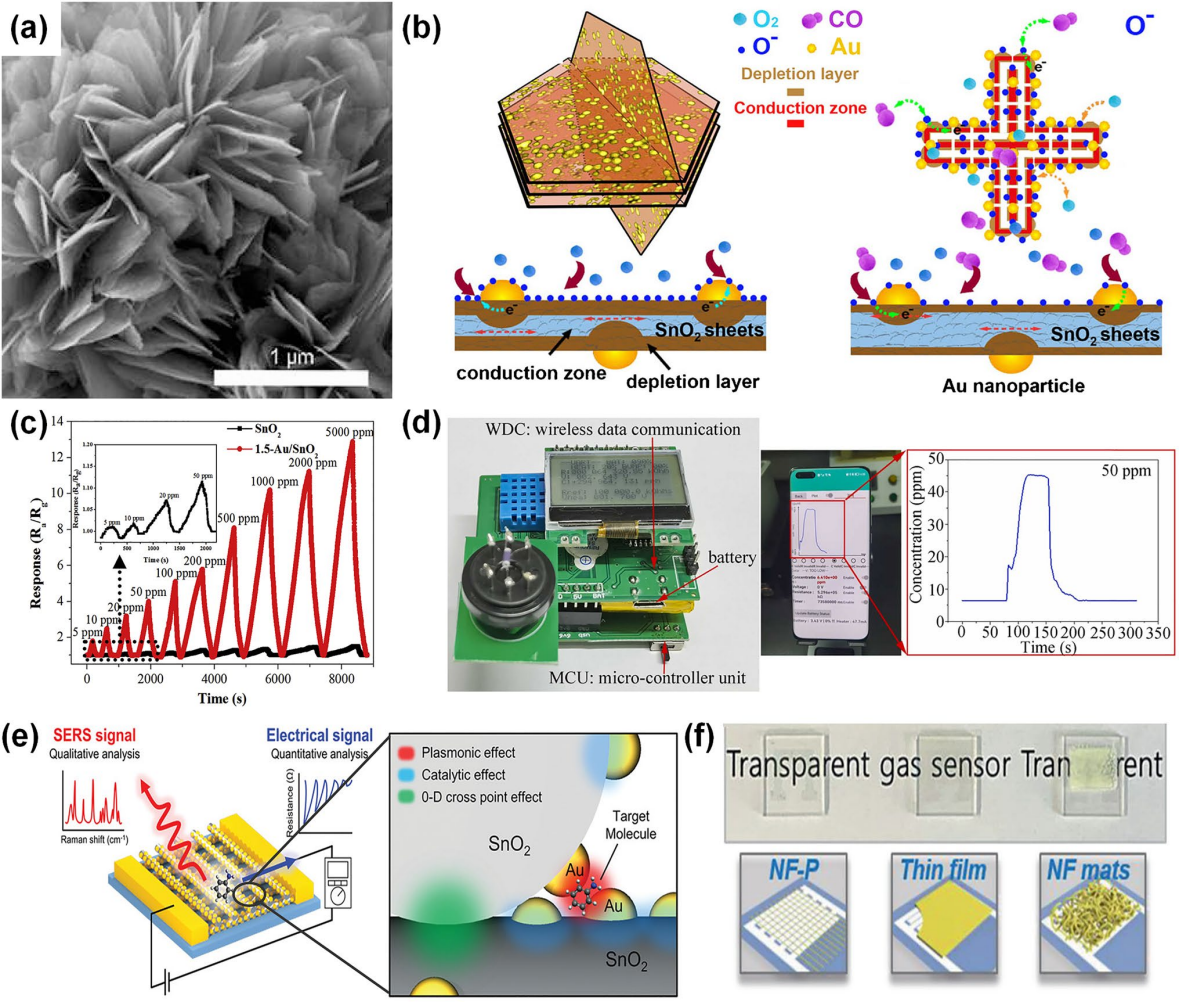
**a** SEM image of Au/SnO_2_ microstructures. Reproduced with permission from Ref. [225]. Copyright 2016, Elsevier. **b** Schematic view of sensing mechanism to Au-loaded SnO_2_ samples. Reproduced with permission from Ref. [224]. Copyright 2016, Elsevier. **c** Responses of the SnO_2_ and 1.5-Au/SnO_2_ sensors towards different CH_4_ concentrations at 120 °C. Reproduced with permission from Ref. [226]. Copyright 2019, Elsevier. **d** Optical photograph of wireless sensing module and dynamic concentration curve of the sensor when exposed to TEA (50 ppm). Reproduced with permission from Ref. [41]. Copyright 2022, Elsevier. **e** Chemo-resistive and SERS multimodal sensing platform with 3D-CMA. Reproduced with permission from Ref. [227]. Copyright 2021, Wiley–VCH. **f** Photograph of transparent Au-SnO_2_ sensor. Reproduced with permission from Ref. [231]. Copyright 2021, Wiley–VCH

3D hierarchical nanostructures are usually high-dimensional nanomaterials periodically assembled from low-dimensional nanomaterials with high surface accessibility and specific surface area, which are attracting attention as highly sensitive gas sensors [224]. As is shown in Fig. 16a, Guo et al. [225] immobilized Au NPs on the surface of 3D SnO_2_ microstructures assembled by 2D porous nanosheets via a solution reduction process. The Au/SnO_2_ sensor exhibited the better performance over pristine SnO_2_ to ethanol due to unique sensitizer properties of Au NPs. The response of Au/SnO_2_ towards 150 ppm ethanol is 29.3, which is 2 times higher than that of pure SnO_2_ (13.7) at 340 °C. Moreover, Bing et al. [224] successfully grew flowerlike SnO_2_ nanosheets assembled from multilayered walls and hexagonal mesoporous. The Au loading SnO_2_ hollow nanosheets exhibited more favorable performance to CO compared with pure SnO_2_. The sensing mechanism of Au-loaded SnO_2_ samples is shown as Fig. 16b. The improved CO sensing response can be ascribed to the unique hollow configuration and the introduction of Au. Besides, the Au-decorated SnO_2_ nanoflowers were prepared through a facile impregnation process by Xue et al. [226]. The 1.5 wt% Au-decorated SnO_2_ sensor to 500 ppm CH_4_ had approximately 6 times higher response than that of pristine SnO_2_ at 120 °C on account of Au NPs served as catalysts shown by Fig. 16c. The mechanism of Au-decorated SnO_2_ was also investigated in detail. Loading small size Au NPs on the surface of SnO_2_ not only ensured the nanostructure of SnO_2_ nanofibers, but also increased the specific surface area of SnO_2_ nanofibers. Secondly, the chemical catalysis provided more active sites for the adsorption of oxygen molecules, and promoted the dissociation of the adsorbed oxygen to accelerate capture and release of electrons. Thirdly, because of the different work functions of SnO_2_ and Au, a Schottky junction was formed between Au NPs and SnO_2_ nanofibers, resulting in electron transfer from SnO_2_ to Au. These above three factors led to the remarkably enhanced sensing performance of the Au-decorated SnO_2_.

Cui et al. [210] used a new self-reductive hydrothermal method to synthesize hierarchical Au-loaded SnO_2_ nanoflowers for ethanol detection. The Au-loaded SnO_2_ had BET-specific surface area of 84 m^2^ g^−1^, which was at least 2 times higher than pure SnO_2_ hierarchical structures. The improved response showed a wider detection range of 1–500 ppm for ethanol, more excellent selectivity, and greater long-term stability. In the detection of organic matter, reported by Feng et al., a novel mesoporous Au-SnO_2_ nanosphere structure manifested high response (5.16) at a low temperature (50 °C), quick response (~ 30 s), and low detection limit (0.11 ppm) towards TEA [41]. The gas sensor integrated a wireless module that can obtain gas information from a smartphone for TEA environmental monitoring, as shown in Fig. 16d.

Apart from the 3D structure, other dimensional Au-decorated SnO_2_ nanostructures have also been developed for gas sensors, which have excellent performance in VOC detection. As shown in Fig. 16e, Han et al. [227] successfully prepared a systematically assembled 3D cross-point multifunctional architecture (3D-CMA) based chemo-resistive/surface enhanced Raman spectroscopy (SERS) multimodal sensor, whose sensing elements were integrated with SnO_2_ nanowires and Au metallic NPs. The 0D contacting points can enhance chemo-resistive sensing response and the high density cross-points are used to massive SERS (optical) signal improvement. Not only detecting ppm-level VOCs, but also identification of gas molecular structure was realized by 3D-CMA-based multimodal sensor. The sensor with excellent performance, low-cost, and good reusability will be applied in various fields, including biomedical diagnosis, food safety and quality monitoring, environmental detection, and explosive detection. What’s more, Feng et al. [228] investigated the xylene gas sensing properties of the Au-SnO_2_ nanorods. Compared with pure SnO_2_, the enhanced performance of 6 and 12 mol% Au-SnO_2_ nanorods was displayed, such as a higher response, lower working temperature, shorter response/recovery time, and greater selectivity. The introduced Au NPs obviously increased the surface-adsorbed oxygen, which is consistent with the XPS results. From the point of view on detecting organophosphorus compounds, preparing gas sensors that combine fast response/recovery time with low detection limits remains challenging. Recently, Yang et al. [229] fabricated a dimethyl methyl phosphonate (DMMP) sensor from the Au NPs deposited onto SnO_2_ based on ceramic tube. Further decoration of Au NPs as the sensitizer can achieve apparent sensing enhancement in terms of low detection limit of 4.8 ppb, fast response/recovery time (26/32 s), and good selectivity. The research can extend a feasible way for quick detecting trace organic compounds. In addition, Lian et al. [230] developed a promising material of Au-SnO_2_ NPs synthesized by a simple hydrothermal method for n-butanol gas monitoring. Experimentally, the response of Au-SnO_2_ NPs (251.23) is over 21 times higher than that of pristine SnO_2_ (11.5) at 200 ppm n-butanol. The sensor based on Au-decorated SnO_2_ NPs showed low detection limit (1 ppm), good stability, and outstanding selectivity towards n-butanol.

Except the development of VOC gas sensors, Lim et al. [231] reported a transparent nanopatterned sensor composed of 1D Au-SnO_2_ nanofibers on the indium tin oxide (ITO) transparent electrodes, which realized the unique room-temperature NO_2_ detection under visible light irradiation, as is shown in Fig. 16f. The nanopattern Au-SnO_2_ nanofibers were prepared on a glass substrate with transparent electrodes by direct-write and near-field electrospinning methods, which contribute to low coverage (0.3%) and high transparency (93%). The as-prepared sensor exhibited a superior sensitivity, selectivity, and reproducibility to sub-ppm levels of NO_2_, whose detection limit isas low as 6 ppb. The unique RT NO_2_ sensor assisted by the visible light is derived from the localized surface plasmonic resonance effect of Au NPs, enabling the sensor to require no external heater or light source. This work provides an application strategy in air quality assessment under indoor natural sunlight or light-emitting diode (LED) illumination, outdoor environmental monitoring, and respiratory analysis for asthma diagnosis.

#### ***Au-Decorated WO***_*3*_*** Gas Sensors***

WO_3_ has served as a promising gas sensing material to detect H_2_S [232], NO_2_ [233], and VOCs [234], and other gases [235] due to its wide energy bandgap, great chemical and thermal stability, as well as higher diffusion coefficient of the oxygen vacancy [205]. Au decoration can be considered as an effective way to enhance the sensing performance of WO_3_ [76].

**Fig. 17:**
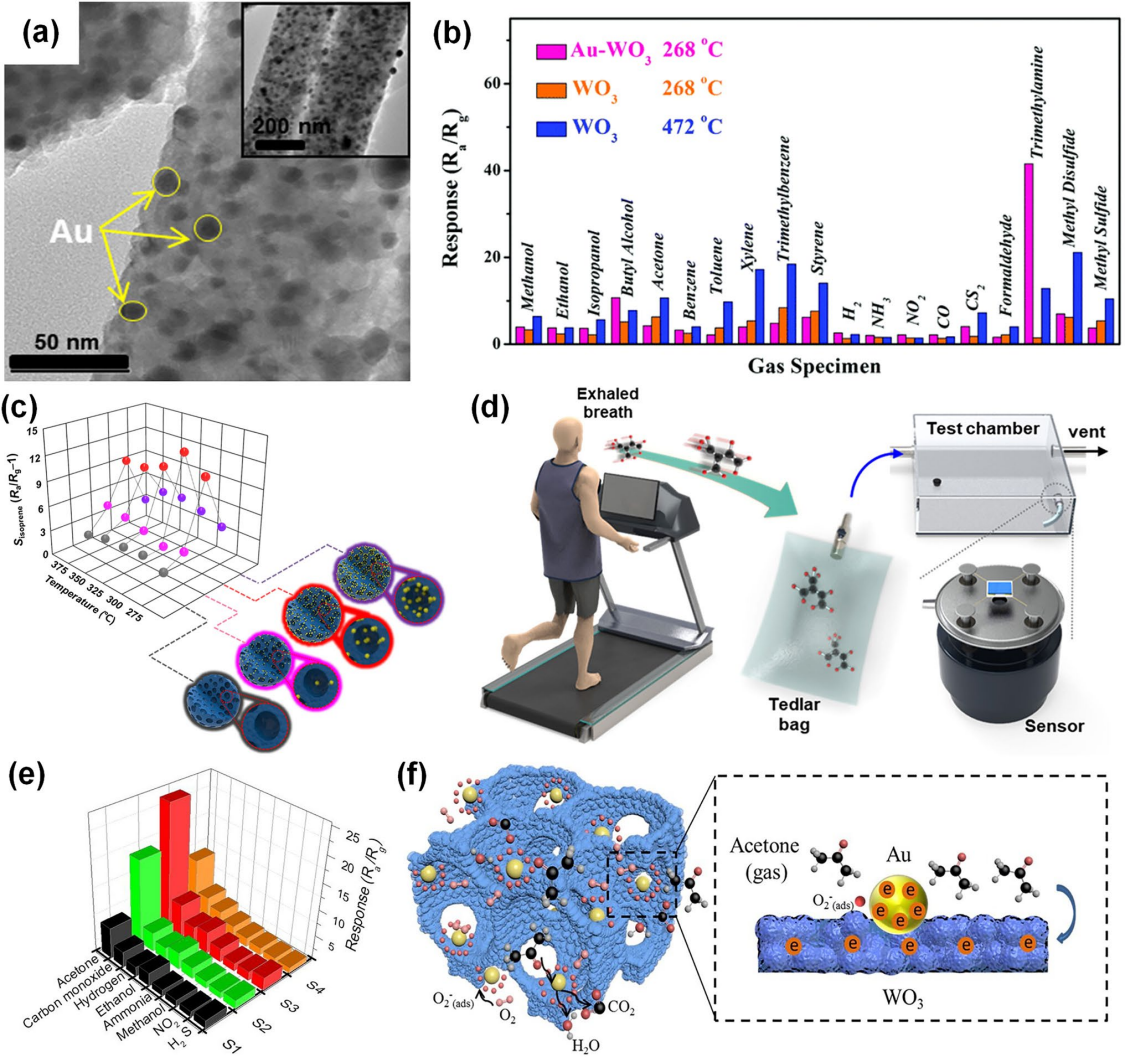
**a** TEM image of Au-WO_3_ composite nanofibers. Reproduced with permission from Ref. [237]. Copyright 2015, Elsevier. **b** Responses of pristine WO_3_ and Au-WO_3_ sensors to various gases at 100 ppm. Reproduced with permission from Ref. [240]. Copyright 2021, Royal Society of Chemistry. **c** Responses of Au@WO_3_ sensors with different Au loading concentrations to 0.1 ppm isoprene at 275 ~ 375 °C under ambient humidity. **d** Schematic of the exhaled breath detection of a normal man during treadmill running based on the Au@WO_3_ sensor. Reproduced with permission from Ref. [241]. Copyright 2022, American Chemical Society. **e** The selectivity behaviors of Au/WO_3_ sensors. **f** Gas sensing schematic diagram of the Au/WO_3_ sensor. Reproduced with permission from Ref. [243]. Copyright 2020, Elsevier

VOCs are ubiquitous, numerous and varied, and some of them can be harmful to human health or damage the natural environment [236]. Au-modified WO_3_ materials have been intensively studied for VOC detection. For example, Laser ablation in liquids (LAL) and annealing methods were used by Dai et al. to prepare Au NPs decorated WO_3_ nanoplatelets [205]. LAL-induced WOx clusters could *in-situ* reduce AuCl_4_^−^ to Au NPs. With following aging treatment, Au-loaded H_2_WO_4_·H_2_O nanoplatelets were formed by self-assembly of colloids. After annealing, Au-decorated WO_3_ nanoplatelets were finally prepared. Compared with pure WO_3_, the Au-WO_3_ nanoplatelets delivered superior response, excellent reproductivity, and lower operating temperature to ethanol, which had potential application in heavy metal ions detection and methanol electro-oxidation. Besides, Yang et al. [237] synthesized Au-decorated WO_3_ composite nanofibers for selective n-butanol sensing. TEM image of Au-WO_3_ composite nanofibers is shown in Fig. 17a, which can be observed that the obtained WO_3_ nanofiber is well- modified with Au NPs with an average particle size of ∼7 nm. The sensing response of the Au-WO_3_ nanofibers was ∼60 times higher than that of pure WO_3_ towards n-butanol, which also showed significantly improved selectivity. The catalytic of Au NPs and the existence of depletion layers at the surface of the Au-WO_3_ nanofibers were considered as the reasons for improving sensing performance. In addition, a HCHO sensor fabricated from Au NPs loaded WO_3_ thin film was reported by Niu et al. [238]. The as-synthesized sensor exhibited a low detection of 40 ppb and extremely high response of 1303.5 under 20 ppm HCHO. For maintaining the desired accuracy, gray algorithm GM (1, 1) and first-order differential approach was used in this study, which is beneficial to implement the HCHO sensor in IoT areas and wireless transmission. Zhang et al. [239] presented the on-chip fabrication of bilayer Au NPs-loaded WO_3_ nanoporous thin films (B-Au/WO_3_) using layer-by-layer stacking of the template-assisted fabrication process with periodic physical Au sputtering deposition. Under the low temperature condition of 150 °C, the sensitivity of B-Au/WO_3_ film to 1 ppm NO_2_ is 96, and the response/recovery time is 9/16 s, which are obviously better than bare WO_3_.

In addition, many porous WO_3_ materials functioned by Au NPs have also received a lot of attention for VOCs detection. For example, Wang et al. prepared mesoporous WO_3_ modified by Au NPs with high specific surface area (17–20 m^2^ g^−1^) to detect trimethylamine (TMA), which is usually presented in seafood spoilage. Figure 17b shows the excellent selectivity for TMA of Au-loaded WO_3_ sensors among various gases including toluene, acetone, ethanol, xylene, and methanol. Furthermore, the prepared sensor displayed significant repeatability, stability, and quick response time (1 s). Besides, accurate detection of exhaled isoprene may provide valuable application for monitoring human physical and physiological state or for early diagnosis of cardiovascular disease. However, since the concentration of isoprene in exhaled gas is extremely low, the development of a highly selective and sensitive isoprene sensor still faces various challenges [240]. In a recent study, Park et al. [241] synthesized Au NPs modified macroporous WO_3_ microspheres, which displayed prominent response of 11.3 to 0.1 ppm isoprene and a relatively low detection limit (0.2 ppb). Meanwhile, as shown in Fig. 17c the authors systematically investigated the effect of the Au loading concentration on the gas sensing performance of Au@WO_3_ sensors and found out that in the entire operating temperature range, the Au@WO_3_ sensor with a moderate Au loading concentration exhibited the best isoprene sensing performance. Furthermore, by monitoring the concentration of isoprene exhaled by a normal man during running (Fig. 17d), the response of the Au@WO_3_ sensor is linearly correlated with the concentration measured by proton transfer reaction quadrupole mass spectrometry (PTR-QMS). The present isoprene sensor has high potential applications for personal healthcare monitoring. Similarly, Lv et al. [242] prepared WO_3_ hollow microspheres with different Au loading concentrations by a wet impregnation method and demonstrated the highest responses of the 1.5 wt% Au-WO_3_ to aromatic compounds, especially to toluene and xylene.In addition, many researches have also been focused on high-performance acetone sensors. For instance, Zhang et al. [243] synthesized 3DIO WO_3_ materials with Au-modification for highly sensitive and selective acetone sensors. Figure 17e displayed the acceptable acetone selectivity of 3DIO Au/WO_3_ sensors among common interferers including CO, H_2_, ethanol, NH_3_, methanol, NO_2_, and H_2_S, which is one of important factors for practical applications. Meanwhile, the reaction mechanism between acetone and Au/WO_3_ is depicted in Fig. 17f. By modifying Au NPs, the catalytic action and spillover effect were generated to promote the response of the 3DIO Au/WO_3_ gas sensor.

Furthermore, Punginsang et al. [244] applied different RF magnetron sputtering time with subsequent annealing to decorate WO_3_ nanowires with different size of Au NPs. The results revealed that when the optimum Au sputtering time is set as 10 s, the H_2_S sensing performance of Au NPs functionalized WO_3_ nanowires is significantly improved in terms of the low detection limit (0.17 ppb) and great sensitivity of 219 to 5 ppm H_2_S. The excellent sensing performance was explained by spillover effects and surface catalytic reactions of Au on heterogeneous interfaces of Au-decorated WO_3_ nanowires.

#### Au-Decorated Other SMOs-Based Gas Sensors

Apart from common SMOs of ZnO, SnO_2_, and WO_3_, many other SMO materials functionalized by Au NPs have also been widely explored for gas sensors, such as CuO [245], Fe_2_O_3_ [246], and MoO_3_ [247].

**Fig. 18:**
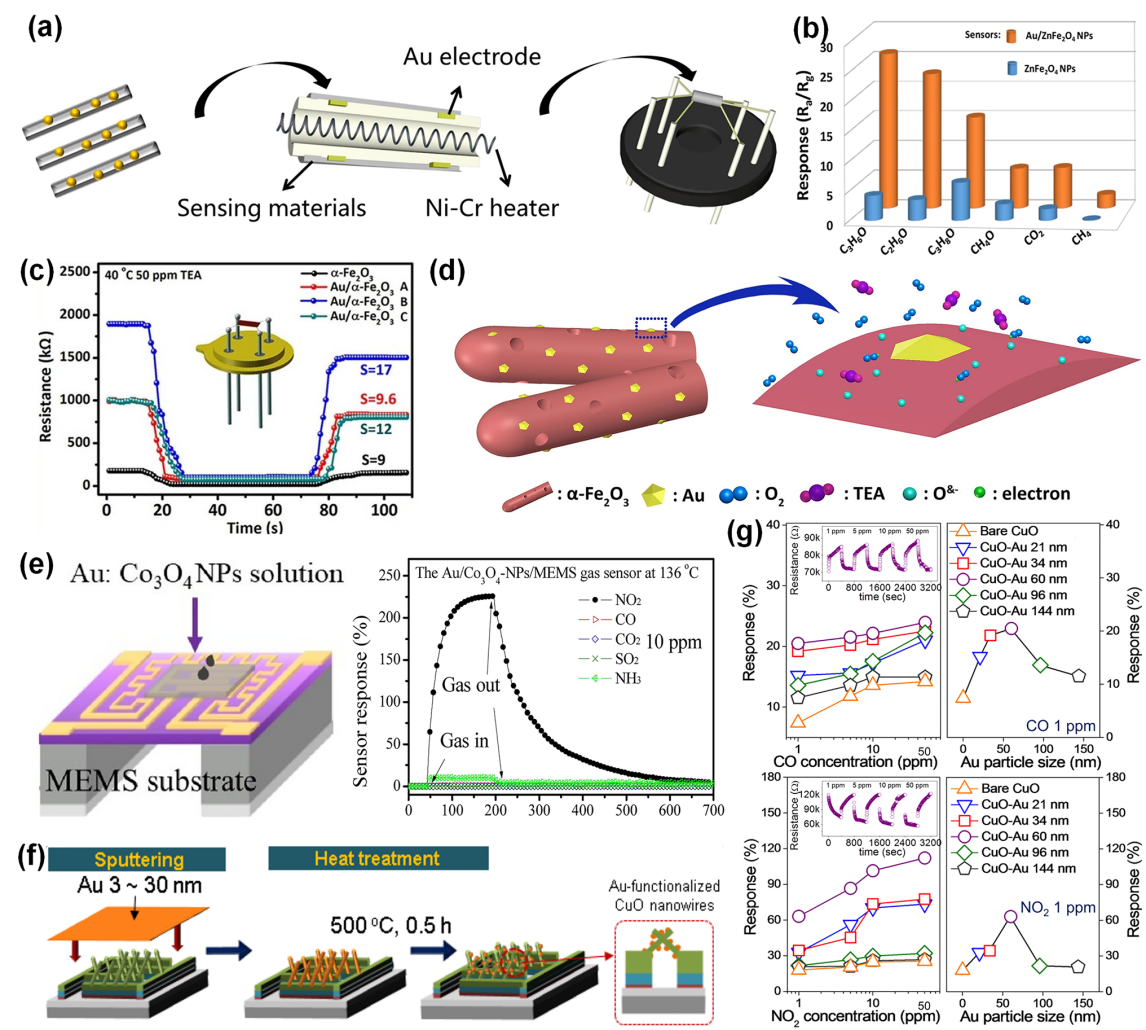
**a** Schematic of preparation of the Au/MoO_3_ sensor. Reproduced with permission from Ref. [248]. Copyright 2021, Elsevier. **b** Responses of Au/ZnFe_2_O_4_ sensor towards 40 ppm of different gases. Reproduced with permission from Ref. [250]. Copyright 2021, Elsevier. **c** Resistance curves of pure-Fe_2_O_3_ nanorods sensor and Au-Fe_2_O_3_ nanorods sensors to 50 ppm TEA at 40 °C. **d** Schematic gas mechanisms of Au-Fe_2_O_3_ nanorods. Reproduced with permission from Ref. [251]. Copyright 2018, Elsevier. **e** Au/Co_3_O_4_-NPs on the MEMS substrate and selectivity towards 10 ppm different gases. Reproduced with permission from Ref. [261]. Copyright 2021, Elsevier. **f** Schematic diagrams of the fabrication process for Au NPs functionalized CuO nanowire. **g** Gas responses of various sizes of Au functionalized CuO nanowire sensors to CO at 350 °C and NO_2_ at 300 °C. Reproduced with permission from Ref. [262]. Copyright 2016, Elsevier

As a typical n-type SMO, 1D MoO_3_ is considered as a high-performance sensing material, which has low conductivity, high crystallinity, and effective gas diffusion and charge transportation. Most importantly, different from traditional surface chemical adsorption mechanism of other MOSs, the sensing mechanism of MoO_3_ is lattice oxygen reaction. The hydrothermal method and *in-situ* reduction were used by Fu et al. to fabricate the 4 wt% Au NPs decorated MoO_3_ nanobelts sensor (Fig. 18a), which showed excellent selectivity to 1-butylamine [248]. The response of the Au/MoO_3_ sensor is more than 3 times higher than that of pristine MoO_3_ nanobelts towards 100 ppm 1-butylamine at 240 °C. Furthermore, since 1-butylamine is a significant marker compound in medical and food industries, another type of 1-butylamine sensor is also investigated.

As an n-type SMO, V_2_O_5_ has also become a hot topic in the research of sensor materials in recent years due to its advantages of high stability, low cost, and high corrosion resistance. Yang et al. [249] explored a high efficiency, low cost, and easy operation method for preparing Au modified nanosheet-assembled V_2_O_5_ microflowers (Au/V_2_O_5_). The gas sensing response of the Au/V_2_O_5_ sensor to toxic 1-butylamine were studied. In comparison with pristine V_2_O_5_, the Au/V_2_O_5_ sensor demonstrated lower operating temperature (~ 240 °C), high sensitivity of 7.5 towards 100 ppm 1-butylamine, and excellent selectivity. This study provides a new insight for preparing noble metal functionalized V_2_O_5_ composites with high sensing properties.

In addition, the spinel type zinc ferrite (ZnFe_2_O_4_) nanostructure is demonstrated to be beneficial to the detection of VOCs. However, its poor sensing capabilities impede its practical application. According to Nemufulwi et al. [250], the sensing response towards acetone of ZnFe_2_O_4_ was improved by modifying it with Au, in terms of high response and selectivity in Fig. 18b. The XPS and photoluminescence (PL) results demonstrated that Au decorated ZnFe_2_O_4_ nanostructures have a high defect concentration, which promote oxygen chemical absorption. The as-prepared Au-ZnFe_2_O_4_ sensor have a good application prospect in the food industry. What’s more, TEA is an explosive gas that can irritate skin, eyes, and respiratory system in concentrations higher than 10 ppm in air. Hence, Song et al. [251] prepared Au decahedrons (DHs)-modified porous Fe_2_O_3_ nanorods for TEA gas sensor, which exhibited low detection limit (~ 1 ppm) and high response at 40 °C towards 50 ppm TEA, as is shown in Fig. 18c. The related sensing mechanism of Au-Fe_2_O_3_ nanorods was also discussed (Fig. 18d). The catalytic activity of Au DHs and the Schottky barrier in the surface of Au/Fe_2_O_3_ were regarded as the reason for the improved sensing performance. The as-synthesized TEA sensor will have widely application potential in chemical industry.

Besides, TiO_2_ has the characteristics of high electrochemical and catalytic activity, low cost, and high chemical stability, which is widely studied as a gas sensing material [252]. However, RT TiO_2_-based sensors with excellent sensitivity and selectivity are still scarce and therefore are highly needed. Mintcheva et al. [253] prepared Au NPs-decorated TiO_2_ NPs using the LAL method. LAL ablates a solid (usually metal) target immersed in a liquid medium to synthesize NPs with different sizes via a laser beam, which is simple, environment friendly, and cost-efficient. The TiO_2_ with 1 and 5 wt% Au loading displayed a maximum response and selectivity towards acetaldehyde and benzene, respectively. Experimentally, oxygen vacancies and Ti_3_^+^ ions were formed on the surface of TiO_2_ after laser irradiation, while the defects of the latter disappeared after modification with Au NPs, which enhanced the sensing response as well as selectivity.β-Ga_2_O_3_ has a wide bandgap of ~ 4.7 eV at RT and characteristics of high thermal, mechanical, and chemical stability [254, 255]. Weng et al. [256] fabricated the sensor based on Au modified β-Ga_2_O_3_ nanowires via vapor–liquid-solid method for RT CO detection with great response/recovery time (5.85/10.13 s) and high sensitivity of 7.8% at RT condition towards 100 ppm CO. Meanwhile, the point defect was oriented to form conductive channel because of applying electric field. Thus, the Au modified β-Ga_2_O_3_ device exhibited bipolar resistive switching behavior.

Compared with n-type SMOs, p-type SMOs demonstrate several advantages such as lower humidity dependence and good selective oxidation ability towards various VOCs [257, 258]. Since the formation of the space charge layer of the hole-accumulation space in p-type SMOs is not limited by the free carrier concentration, p-type SMOs also have the excellent ability to chemisorb high concentration of oxygen [259]. Co_3_O_4_ is a typical p-type SMO material with cubic spinel structure, whose surface could adsorb more oxygen than other p-type SMOs [260]. Hsueh et al. [261] produced the MEMS-based Au NPs adsorbed Co_3_O_4_ NPs sensor through an ultrasonic wave grinding technology and a solution method for NO_2_ detection. The corresponding MEMS device is shown in Fig. 18e. The prepared MEMS-based Au/Co_3_O_4_-NPs sensor has the highest response of 33% to 100 ppb NO_2_ and more excellent sensitivity towards NO_2_ than other gases (CO, NH_3_, SO_2_, CO_2_) at 136 °C (in Fig. 18e). This study integrating Au/Co_3_O_4_-NPs materials and MEMS technology into devices are suitable to wearable applications and have promisingly used for sensing networks in the IoT.

In addition, Lee et al. [262] investigated the influence of the Au NP size on gas sensing properties of p-CuO nanowires. The Au NPs functionalized CuO nanowires were realized by heat treatment of the Au layer grown by sputtering, as shown in Fig. 18f. The responses to CO and NO_2_ of Au modified CuO nanowires with various Au-NP diameters is shown in Fig. 18g. The Au-CuO nanowires with an average diameter of 60 nm had the highest response to 1 ppm CO and NO_2_, which reveal more clearly that optimizing the size of Au NPs can obtain excellent sensing performance.

#### Au-Decorated Heterostructured SMOs-Based Gas Sensors

Using different SMOs to construct heterogeneous structures has become a favorable candidate for the preparation of high-performance gas sensors [263, 264]. Besides, through the decoration of Au NPs on heterostructures, the obtained sensing materials can achieve superior sensing performance.

**Fig. 19:**
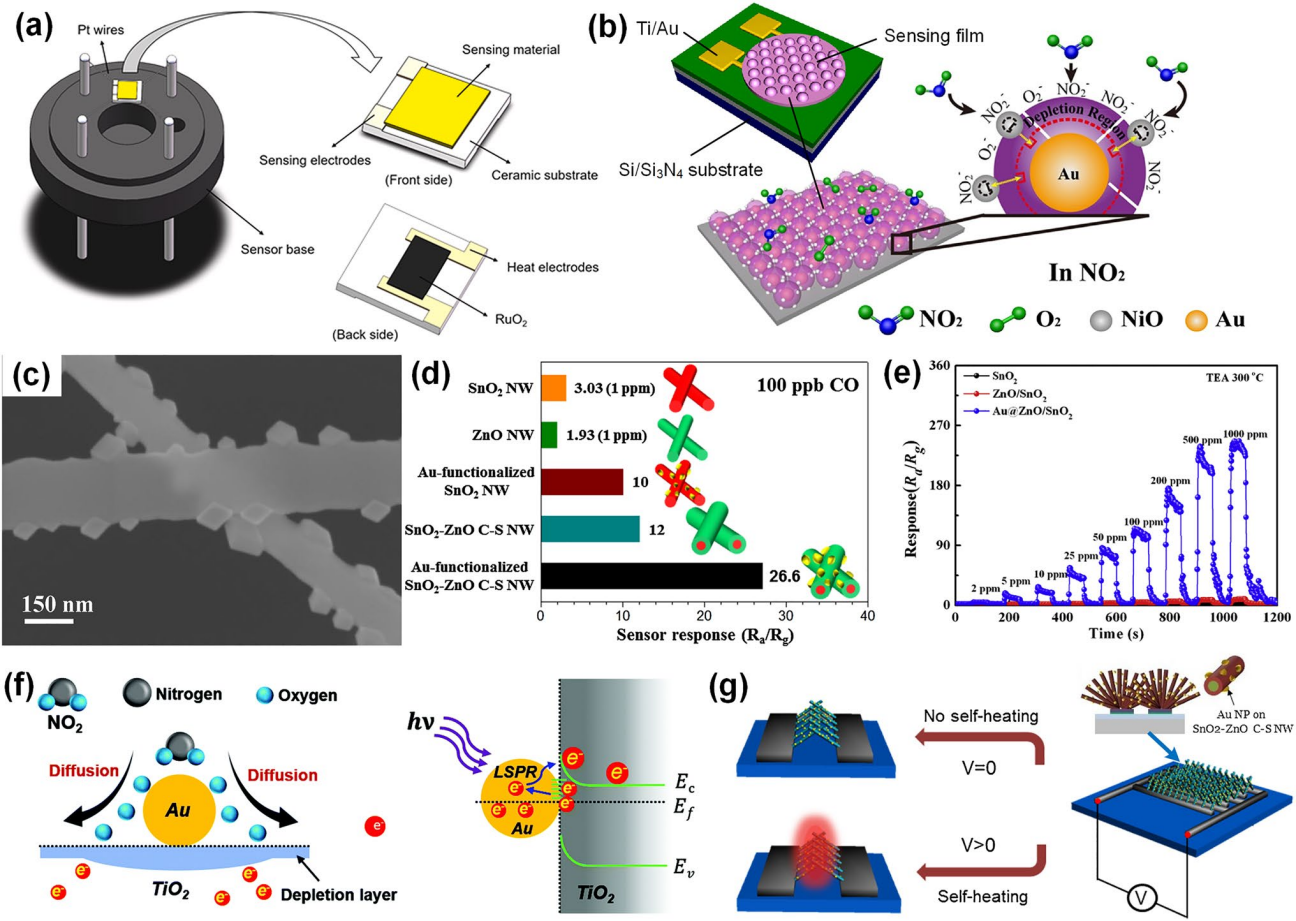
**a** Sensor device diagram of MOFs derived Au@Cr_2_O_3_-In_2_O_3_. Reproduced with permission from Ref. [265]. Copyright 2022, Elsevier. **b** Schematic diagram of MEMS device and sensing mechanism. Reproduced with permission from Ref. [266]. Copyright 2019, Elsevier. **c** SEM image of the Au-ZnO/In_2_O_3_ heterostructures. Reproduced with permission from Ref. [267]. Copyright 2017, Elsevier. **d** Comparative histograms of five types of nanowires responses to CO at 300 °C. Reproduced with permission from Ref. [268]. Copyright 2017, Elsevier. **e** Response curves of SnO_2_, ZnO/SnO_2_, and Au@ZnO/SnO_2_ sensors to different concentrations of TEA at 300 °C. Reproduced with permission from Ref. [269]. Copyright 2018, Elsevier. **f** Spillover effect of Au NPs and the charge transfer process between Au and TiO_2_. Reproduced with permission from Ref. [271]. Copyright 2021, Royal Society of Chemistry. **g** Schematic diagram of Au-decorated SnO_2_-ZnO nanowires sensor with/without self-heating. Reproduced with permission from Ref. [272]. Copyright 2018, Elsevier

In terms of research on Au-modified anisotype heterojunction, in order to realize the trace detection of VOCs, Wu et al. fabricated a sensor based on MOFs derived Au@Cr_2_O_3_-In_2_O_3_ nanorods, as is depicted in Fig. 19a. The Au@Cr_2_O_3_-In_2_O_3_ sensor enabled ppb-level detection of isoprene, ethanol, and HCHO with concentrations of 50, 200, and 200 ppb, respectively. Meanwhile, the sensor displayed a superior response to other nonvolatile respiratory biomarkers of, CO, H_2_, NO_2_, and NH_3_. Remarkably, the sensor had good long-term stability even at high humidity. The results reveal that the heterojunction of Cr_2_O_3_-In_2_O_3_ and electron sensitization of Au synthetically improve the gas sensing properties. The as-prepared Au@Cr_2_O_3_-In_2_O_3_ nanorods can be a significant candidate for the breath diseases monitoring [265]. Besides, Wang et al. explored the potential of MEMS compatible heterostructure manufacturing methods to produce wafer-level gas sensors. Specifically, they prepared Au NPs functionalized SnO_2_-NiO thin films using magnetron sputtering and self-assembly technique for NO_2_ gas detection. The MEMS device is shown in Fig. 19b, with interdigitated Au electrodes fabricated on Si/Si_3_N_4_ substrates by lithographic techniques. The sensor exhibited a low limit of detection (50 ppb), high sensing response of 185 towards 5 ppm NO_2_, low sensor-to-sensor variation of < 15%, as well as good stability and selectivity. The *in-situ* NO_2_ detection achieved by the Au NPs functionalized SnO_2_-NiO thin films sensor can be used to assess air quality [266].

Among numerous Au-decorated isotype heterojunctions, Wang et al. [267] synthesized belt-tooth shape Au NPs functionalized ZnO/In_2_O_3_ nano-heterostructures via a CVD process and sputtering method (Fig. 19c), which displayed superior gas sensing performance for C_2_H_2_ at a low working temperature of 90 °C. Besides, the C_2_H_2_ sensor manifested faster response, greater selectivity, and good long-term stability in 30 days. The small size Au NPs as catalysts can be beneficial to promote the C_2_H_2_ gas sensing response. In addition, ultra-sensitive CO sensors based on Au NPs functionalized SnO_2_-ZnO core–shell nanowires have been achieved by Kim et al. [268]. Compared with other nanowires sensors towards CO, the response of obtained sensor is extremely high at a low CO concentration of 100 ppb, which is shown in Fig. 19d. Another study on Au modification of ZnO and SnO_2_ heterostructures by Zhai et al., a low-cost hydrothermal synthesis and pulsed laser deposition (PLD) method were employed to synthesize uniform ZnO/SnO_2_ nanosheets *in-situ* grew on the Al_2_O_3_ tubes [269]. The Au NPs modification on ZnO/SnO_2_ nanosheets was realized through a direct current (DC)-sputtering process. As shown in Fig. 19e, the synthesized Au@ZnO/SnO_2_ Nanosheets showed prominent sensing response of 115 towards 100 ppm TEA at 300 °C, which was about 20 times stronger than that of the pure SnO_2_ nanosheets. In addition, Xu et al. also fabricated TEA sensors from Au NPs functionalized SnO_2_/α-Fe_2_O_3_ core–shell nanoneedles on Al_2_O_3_ tubes. According to Xu et al. [270], the Au@SnO_2_/α-Fe_2_O_3_ nanoneedles exhibited excellent gas sensing performance, such as superior selectivity to TEA and linearity (R = 0.9975) of sensing properties than of pristine SnO_2_ and SnO_2_/α-Fe_2_O_3_ sensors. The gas sensing mechanism of the above two TEA sensors with different Au NPs modified heterostructures can be explained as Schottky contact between Au and shell layer, as well as the formation of n–n heterojunction. Such TEA sensors can be significant candidates to realize an accurate detection of TEA in food industries, chemical, biomedical, and our daily life.

UV photoactivation can perform to reduce the operating temperature of SMO-based gas sensors. For example, Kwon et al. [271] prepared Au NPs decorated ZnO/TiO_2_ core–shell nanorods for NO_2_ gas detection. The fabricated ZnO/TiO_2_ nanorods decorated with Au NPs demonstrated 9 times higher gas sensitivity and shorter response/recovery time than ZnO nanorods based sensors. The enhanced NO_2_ sensing performance is attributed to both the formation of ZnO/TiO_2_ heterojunction and the catalytic sensitization effect of Au NPs. Under UV light irradiation, the decorated Au NPs will absorb the light produced by UV-LED photo-activated ZnO and TiO_2_ defect layer, and transfer the electrons remaining in the defect layer to the conduction band due to localized surface plasmon resonance (LSPR) effect, so as to increase the number of high-energy electrons (Fig. 19f). According to Kim et al. [272], a self-heating CO gas sensor composed of Au-decorated SnO_2_-ZnO core–shell nanowires was developed. As shown in Fig. 19g, the temperature rise caused by Joule heating effect enhanced the sensor response at high applied voltage. The CO sensor not only demonstrated excellent selectivity without the external heater, but also had extremely low power consumption in the range of 0.81 nW-8.3 μW at 1–20 V. This extremely low power sensor is a promising choice for integrated sensor arrays and wireless sensor applications. In a word, Au NPs decoration provides an idea for improving the gas sensing performance of heterostructured gas sensors and promote the application in many fields (Table 3).

**Table 3:** Summary of the reported Au-decorated SMOs-based gas sensors

Material	Structure	Synthesis method	Target gas	O. T. (°C)	Conc. (ppm)	Response	t_res_/t_rec_	LOD	Refs.
ZnO	Nanowires	Hydrothermal	Ethanol	380	100	33.6^a^	3/1 s	2 ppm	[214]
ZnO	Nanowires	Hydrothermal	Ethanol	200	50	151.86^a^	22/23 s	5 ppm	[215]
ZnO	Nanowires	Hydrothermal	H_2_	25	5000	470^a^	10/10 s	20 ppm	[273]
ZnO	Thick films	Sputtering	C_2_H_2_	325	25	5.8^a^	5 /320 s	3 ppb	[216]
ZnO	Hierarchical nanostructure	UV-assisted deposition	Ethanol	220	1000	323.69^a^	–	–	[217]
ZnO	Nanosheets	Hydrothermal	Acetone	300	100	1012.6^a^	–	0.2 ppm	[209]
ZnO	Nanosheets	Hydrothermal	Ethanol	300	60	35%^b^	15 s/–	–	[219]
ZnO	Nanosheets	Solvothermal	Acetone	275	100	164^a^	3/5 s	5 ppm	[274]
ZnO	Nanofilms	Thermal evaporation	Methyl mercaptan	250	0.05	4.99%^b^	134/368 s	50 ppb	[203]
ZnO	Microspheres	Impregnation	CH_4_	250	100	4.16^a^	–	1.83 ppm	[275]
ZnO	Nanoplates	Hydrothermal	Ethanol	300	200	45^a^	13 s/–	5 ppm	[276]
ZnO	Nanoflowers	Hydrothermal	Acetone	270	100	74.41^a^	5/3 s	5 ppm	[221]
ZnO	Nanoflowers	Hydrothermal	TEA	200	10	276^a^	20/216 s	0.17 ppb	[277]
ZnO	Nanoparticles	Hydrothermal	Ethanol	400	100	525^a^	3 s/–	1 ppm	[220]
ZnO	Nanoparticles	Solvothermal	Isoprene	350	1	1371^a^	~ 50/ ~ 5 s	6 ppb	[278]
ZnO	Nanospheres	Photoreduction	Ethanol	200	50	159^a^	–	0.2 ppm	[279]
SnO_2_	3D microstructure	Hydrothermal	Ethanol	340	150	29.3^a^	5/10 s	5 ppm	[225]
SnO_2_	Nanosheets	Hydrothermal	CO	220	50	36.5^a^	1/4 s	1 ppm	[224]
SnO_2_	Nanoflowers	Hydrothermal	CH_4_	120	500	8^a^	–	5 ppm	[226]
SnO_2_	Nanoflowers	Self-reductive	Ethanol	200	100	123^a^	3/6 s	1 ppm	[210]
SnO_2_	Nanospheres	Direct thermal decomposition	TEA	50	5	5.16^a^	~ 30 s/–	0.11 ppm	[41]
SnO_2_	Nanowires	Electron-beam evaporation	Nitrobenzene Toluene Benzene	350	5	5^a^ 2.6^a^ 7.6^a^	–	0.25 ppm	[227]
SnO_2_	Nanorods	Hydrothermal	Xylene	279	50	7.9^a^	8/4 s	3 ppm	[228]
SnO_2_	Nanoparticles	*In-situ* reduction	DMMP	320	0.034	1.22^a^	–	34 ppb	[229]
SnO_2_	Nanoparticles	Hydrothermal	N-butanol	240	50	251.23^a^	3/11 s	1 ppm	[230]
SnO_2_	Nanofibers	Hydrothermal	NO_2_	22.4	5	42^a^	–	6 ppb	[231]
SnO_2_	Nanofibers	Reduction	N-butanol	340	300	667^a^	30/60 s	–	[280]
SnO_2_	Mesoporous spheres	Hydrothermal	N-butyl alcohol	300	300	154^a^	50/35 s	10 ppm	[281]
SnO_2_	Nanospheres	Sol–gel process	Ethanol	250	100	80^a^	16/53 s	1 ppm	[223]
WO_3_	Nanofibers	Hydrothermal	N-butanol	250	10	63.6^a^	31/9 s	1 ppm	[237]
WO_3_	Thin films	Vacuum thermal evaporation	HCHO	225	20	1303.5^a^	16/16 s	40 ppb	[238]
WO_3_	Thin films	Sputtering	NO_2_	150	1	96.0^a^	9/16 s	0.6 ppm	[239]
WO_3_	Mesoporous structure	Impregnation	TMA	268	100	41.56^a^	1 s/-	5 ppm	[240]
WO_3_	Macroporous spheres	Spray pyrolysis	Isoprene	275	0.1	11.3^a^	64/1204 s	0.2 ppb	[241]
WO_3_	Hollow microspheres	Wet impregnation	Toluene Xylene	340	10	6.7^a^ 8.5^a^	8/5 s 10/5 s	0.3 ppm	[242]
WO_3_	3D nanostructure	Sacrificial template	Acetone	410	100	10.76^a^	7/8 s	100 ppb	[243]
WO_3_	Nanowires	Sputtering	H_2_S	350	5	219^a^	2/20 min	0.17 ppb	[244]
WO_3_	Nanoplates	*In-situ* reduction	H_2_S	50	10	220^a^	> 200/ > 1000 s	0.5 ppm	[232]
WO_3_	Thin films	Spin-coating	NO_2_	150	5	249^a^	~ 240/ ~ 50 s	28 ppt	[233]
WO_3_	Nanowires	Solvothermal	N-butanol	250	100	147^a^	19/25 s	5 ppm	[234]
			Acetone		200	72^a^	17/24 s		
WO_3_	Nanoplates	Chemical reduction	NO	170	10	216^a^	24/26 s	0.5 ppm	[235]
WO_3_	Microspheres	Hydrothermal	NO_2_	50	5	15.6^a^	~ 50/ ~ 85 s	1 ppm	[282]
WO_3_	Nanosheets	Chemical reduction	NO_2_	175	5	212.3^a^	90/ ~ 110 s	50 ppb	[283]
MoO_3_	Nanobelts	*In-situ* reduction	1-butylamine	240	100	~ 300^a^	23/388 s	1 ppm	[248]
V_2_O_5_	Nanoflowers	*In-situ* reduction	1-butylamine	250	100	7.3^a^	48/11 s	5 ppm	[249]
α-Fe_2_O_3_	Nanorods	One-pot polyol reaction	TEA	40	50	17^a^	12/8 s	~ 1 ppm	[251]
ZnFe_2_O_4_	Nanostructures	Microwave-assisted hydrothermal	Acetone	120	40	25^a^	3/13 s	2.5 ppm	[250]
TiO_2_	Pecan-kernel-like nanostructure	Precipitation	Toluene	375	100	7.3^a^	4/5 s	10 ppm	[252]
TiO_2_	Nanoparticles	Laser irradiation	Ammonia Acetaldehyde Benzene	29	200	65^a^ 115^a^ 55^a^	–	–	[253]
Co_3_O_4_	Nanoparticles	Reduction	NO_2_	136	0.1	35%^b^	84/68 s	10 ppb	[261]
CuO	Nanowires	Sputtering	CO NO_2_	350 300	1	20%^b^ 61%^b^	–	–	[262]
Fe_2_O_3_	Nanorods	Impregnation	N-butanol	250	100	177^a^	126/49 s	5 ppm	[284]
Cr_2_O_3_/In_2_O_3_	Nanorods	Reduction	Isoprene Ethanol HCHO	180	1	6.4^a^ 10.6^a^ 4.3^a^	52/443 s 135/618 s –	50 ppb 200 ppb 200 ppb	[265]
SnO_2_/NiO	Thin films	Sputtering	NO_2_	200	5	180^a^	–	50 ppb	[266]
ZnO/In_2_O_3_	Belt-tooth shape	Sputtering	C_2_H_2_	90	100	5^a^	8.5 s/–	–	[267]
SnO_2_/ZnO	Nanowires	Vapor–liquid–solid growth	CO	300	0.1	26.6^a^	–	100 ppb	[268]
ZnO/SnO_2_	Nanosheets	Sputtering	TEA	300	100	115^a^	7/30 s	2 ppm	[269]
SnO_2_/α-Fe_2_O_3_	Nanoneedles	Sputtering	TEA	300	200	63^a^	–	10 ppm	[270]
ZnO/TiO_2_	Nanorods	Hydrothermal	NO_2_	RT	50	7.5^a^	–	10 ppm	[271]
WO_3_/SnO_2_	Nanofibers	Hydrothermal	Acetone	150	0.5	79.6^a^	–	0.2 ppm	[285]
ZnO/SnO_2_	Nanofibers	Reduction	H_2_S	350	1	~ 75^a^	36/786 s	0.1 ppm	[286]
SnO_2_/In_2_O_3_	Nanofibers	Sputtering	CO	200	50	29.2%^b^	< 30/ < 30 s	–	[264]

*O. T.* operating temperature; *Conc*.: concentration; *t*_*res*_*/t*_*rec*_ response time/recovery time; *LOD* limit of detection

^a^Response is defined as R_a_/R_g_ or R_g_/R_a_, R_a_: resistance of the sensor in air, R_g_: resistance of the sensor exposed to target gas

^b^Response is defined as ∆R/R_a_ $\times$ 100% or ∆R/R_g_ $\times$ 100%, ∆R: the change in resistance, which equals to |R_a_–R_g_|

### Other Noble Metal-Decorated SMOs-Based Gas Sensors

#### Ag-Decorated SMOs-Based Gas Sensors

Ag, as a relatively cheap metal compared with other noble metals, exhibits unique advantage in gas sensing performance because of its high solubility, larger size of ionic, and minimum orbital energy [287]. Ag NPs decoration could promote the sensitivity of the SMOs-based gas sensor, decrease the working temperature, and shorten the response/ recovery time due to the unique catalytic activity of Ag NPs [288]. By decorating Ag NPs on SMOs, more active sites were provided for adsorbed oxygen ions contributing to more efficient electron transfer in gas sensing applications. The adsorption activation energies of gases were reduced by decorating Ag NPs, and the active surface sites generated by Ag NPs accelerated the adsorption of the gas molecule, which promoted the gas sensing properties [289, 290]. Moreover, the Ag NPs decorated on the SMOs could catalyze the dissociation of the gas molecules and promote the surface reaction. On the basis of the chemical sensitization effect, the Ag NPs served as potential sites for the oxygen molecules which adsorbed or desorbed on the materials [291]. Particularly, the electronic sensitization was determined by the existence of a potential barrier between the SMOs and the Ag NPs. The different Fermi levels between the SMO material and Ag NPs lead to the accumulation or depletion of charge carriers [292]. In conclusion, decorating Ag NPs on SMOs is a potential strategy for enhancing gas sensing behavior. The Ag-decorated gas sensors were demonstrated to have excellent sensing behavior towards many kinds of target gases in the current studies. According to reported literatures**,** the Ag-decorated gas sensors were most extensively applied to detect ethanol, HCHO, and NO_2_.

**Fig. 20:**
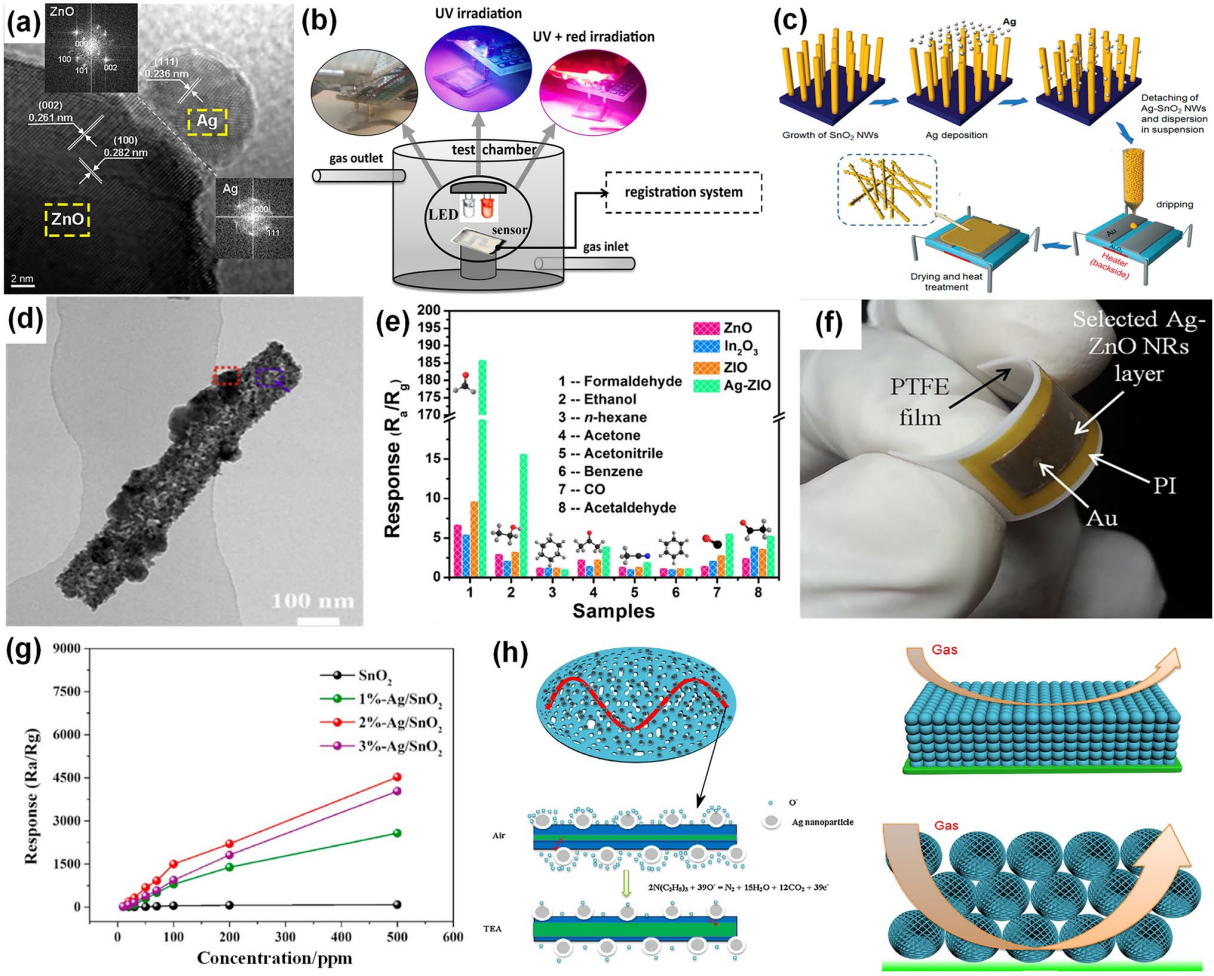
**a** TEM image of a single Ag NP decorated on a ZnO NP. Reproduced with permission from Ref. [301]. Copyright 2018, Elsevier. **b** Schematic of the gas-testing measurements of the Ag-decorated ZnO NPs gas sensor with UV and optimized red irradiation. Reproduced with permission from Ref. [303]. Copyright 2020, Elsevier. **c** Preparation process of the Ag-decorated SnO_2_ nanowires network gas sensor. Reproduced with permission from Ref. [42]. Copyright 2011, American Chemical Society. **d** TEM image of the Ag-ZnO/In_2_O_3_ heterojunction nanofiber. **e** Selectivity of the Ag-ZnO/In_2_O_3_ heterojunction nanofibers gas sensor towards various target gases. Reproduced with permission from Ref. [305]. Copyright 2021, Elsevier. **f** Photograph of the Ag-decorated ZnO flexible gas sensor. Reproduced with permission from Ref. [306]. Copyright 2016, Elsevier. **g** Linearity of the response of the Ag-decorated SnO_2_ nanosheets sensor and the gas concentration. Reproduced with permission from Ref. [307]. Copyright 2022, Elsevier. **h** Sensing mechanism of the 3D Ag-ZnO microspheres sensor with the help of the Ag NPs decoration. Reproduced with permission from Ref. [309]. Copyright 2018, Elsevier

Some strategies were developed to enhance the performance of the Ag-decorated SMOs-based gas sensors, such as designing special nanostructures, increasing surface defects, constructing heterostructures, and introducing light illumination. Different morphologies of Ag-decorated SMOs materials such as nanoparticles [293], nanorods [294], nanofibers [295], nanowires [42], nanosheets [296], microspheres [297], and nanoflowers [298], were studied for the enhancement of gas sensing properties with high sensitivity, lower operation temperatures, lower detection limit, and remarkable selectivity. Owing to uniform distribution and abundant surface oxygen vacancies, the Ag-decorated nanomaterials display strong catalytic activity compared with pure nanomaterials and high photocatalytic efficiency applied in light-assisted gas sensors. Wang et al. [299] fabricated the SnO_2_-reduced graphene oxide gas sensor decorated with Ag NPs through a two-step wet-chemical method, and the as-prepared material displayed good NO_2_ detection ability operating at RT. This work also showed that decorating Ag NPs was an efficient strategy to shorten the response and recovery time. Dong et al. [300] proposed an Ag-decorated In_2_O_3_/ZnO gas sensor via a nonaqueous route. The results exhibited that functionalizing Ag NPs with a reasonable Ag NPs concentration of 3 wt% could enhance the response about 842.9 towards 2000 ppm HCHO at 300 °C. The heterostructure between In_2_O_3_ and ZnO and the influence of Ag NPs decoration resulted in the excellent gas sensing properties. At present, many novel Ag-decorated nanomaterials have been synthesized to promote the gas sensing behavior due to their catalytic ability as well as the synergic interaction. However, the traditional Ag-SMOs-based sensors suffer from high operating temperature, which required the sensors to work under a specific temperature. Fortunately, the light wavelength makes many electrons accumulate at the surface of the nanomaterials and further increase the surface adsorbed oxygen. Therefore, the light-assisted gas sensor was potential to detect the target gases at the RT. Typically, some studies focused on the light-assisted Ag-decorated SMOs-based sensor for promoting the gas sensing behavior. Among them, ZnO-based gas sensors have accounted for much of this contribution. Zhang et al. [301] fabricated the Ag-loaded ZnO sensor through a modified polymer-network gel method. The TEM image exhibited in Fig. 20a showed that an Ag NP was well decorated on the ZnO NP, which improved the sensing behavior due to its synergistic effect. The as-prepared Ag-decorated ZnO sensor displayed strong and stable response signals even at a very low NO_2_ concentration of 500 ppb. The response of the Ag-decorated ZnO sensor towards 500 ppb NO_2_ was ~ 0.6. In this case, when changing the LED light sources, the maximum response can be acquired. The results revealed that the Ag-decorated ZnO sensor showed the highest response, remarkable stability, and excellent selectivity with the blue-green LED (470 nm, 75 mW cm^−2^). Zhang et al. [302] also synthesized a light-activated Ag-decorated ZnO sensors for NO_2_ detection. In this case, Ag decoration could enhance the visible-light-driven catalytic performances of ZnO nanocatalysts. Moreover, the gas sensing performance degraded slightly under visible light irradiation. Dilova et al. [303] formed Ag-ZnO NPs gas sensor via the PLD method. The Ag NPs decoration on the ZnO nanostructure enhanced the response of sensors and shortened the response/recovery time. The outcomes revealed that UV and optimized red irradiation promoted the sensing performance of the Ag-decorated ZnO NPs gas sensor towards CO, while inhibiting the response towards the target gases like NH_3_, ethanol, and acetone. Figure 20b exhibited the gas-testing equipment for the Ag-decorated ZnO NPs gas senor under light irradiation.

In addition to the 0D nanostructure materials mentioned above, the 1D materials such as nanowires, nanofibers, nanorods, and nanotubes have drawn attention from many researches. Zhou et al. [304] synthesized Ag modified ZnO nanorods-based C_2_H_2_ gas sensor through a solvothermal method. Ag NPs decoration could greatly promote the gas sensing behavior compared with the pristine ZnO nanorods. The high response of the Ag-decorated ZnO nanorods sensor towards 100 ppm C_2_H_2_ was 539 (*R*_a_/*R*_g_). In addition, the operating temperature of the Ag-decorated ZnO nanorods sensors was decreased to 175 °C, and the response of the sensor was increased by 33 times, which exhibited the highest value among most of sensors in detecting. Hwang et al. [42] fabricated high-response Ag-decorated SnO_2_ nanowires ethanol gas sensor. In this case, Ag NPs were decorated on the SnO_2_ nanowire via the e-beam evaporation. The preparation process of Ag-decorated SnO_2_ nanowire gas sensors was illustrated in the Fig. 20c. The SnO_2_ nanowire sensor modified by Ag NPs exhibited a 3.7-fold enhancement towards 100 ppm ethanol compared to the pristine SnO_2_ nanowires sensor. Furthermore, the formation of heterojunction structure was extensively employed to adsorb more oxygen molecules and target gases. Liu et al. [305] proposed a high-sensitivity Ag-decorated ZnO/In_2_O_3_ nanofibers through an electrospinning method. In Fig. 20d, the morphological feature of the Ag-ZnO/In_2_O_3_ nanofibers was exhibited. With the existence of multi-level heterojunctions, the high response of the as-prepared sensor was ~ 186 towards 100 ppm HCHO at 260 °C, which headed the list in HCHO gas sensor research. Beyond that, the as-prepared gas sensor could also detect the HCHO even at a low concentration of 9 ppb. The results shown in Fig. 20e obviously displayed that the Ag NPs decoration could vastly enhance the gas sensing behavior of the sensor towards various gases compared to the pristine ZnO nanofibers and the ZnO/In_2_O_3_ heterojunction nanofibers. Iftekhar Uddin et al. [306] proposed a novel flexible sensor based on Ag-decorated ZnO nanorods. The as-prepared flexible sensor exhibited a high response of 27.2 towards 1000 ppm C_2_H_2_ at 200 °C. As shown in Fig. 20f, the response of the Ag-loaded nanorod flexible gas sensor degraded by a tiny amount ~ 2.1% at a curvature angle of 90°. The successful immobilization of tiny sized Ag NPs and the remarkable flexibility of the Ag-loaded vertical ZnO nanorods gas sensor maintained stable C_2_H_2_ sensing behavior even under the extreme bending stress.

Recently, the morphologies of the nanomaterials formed by well-controlled synthesis processes demonstrated that both the 2D nanostructure and Ag NPs decoration could greatly promote the gas sensing behavior of SMO materials as well. Yu et al. [25] synthesized an Ag NPs-modified 2D WO_3_ nanosheets gas sensor for HCHO detection. The outcomes revealed that the Ag NPs modification made the sensors shorten approximately 3 times of the response time (5 s) and 2 times of the recovery time (10 s). After two months, the response of the Ag-modified WO_3_ nanosheets gas sensor was declined by 8.8% while the pristine WO_3_ sensor was declined by 31.6%, which showed excellent long-term stability. Zhang et al. [307] reported an Ag-decorated SnO_2_ nanosheets gas sensor based on a novel porous nanostructure like fish-scale via a one-step method. Since a reasonable noble metal concentration was crucial to promote the gas sensing behavior, the concentration of the Ag NPs decorated on the SnO_2_ nanosheets was investigated varying from 1 to 3 at% in this case. As shown in Fig. 20g, the 2%-Ag-decorated SnO_2_ nanosheets sensor displayed the highest response value when exposed to the same concentration TEA compared with 1 and 3%-Ag-decorated SnO_2_ nanosheets sensors. The outcomes revealed that the introduction of Ag NPs could greatly promote the adsorption of gas molecules, which could further promote the gas sensing behavior.

Besides, Ag NPs could also be combined with the 3D hierarchical nanostructures, which promoted the gas sensing properties because that they helped to adsorb the gas molecule and promote the electron exchange between the Ag-decorated SMOs and the target gases. Zhang et al. [308] reported the 3D Ag-ZnFe_2_O_4_ hollow sphere through a simple hydrothermal method. The as-prepared Ag-decorated ZnFe_2_O_4_ hollow sphere micromaterials with high specific surface area provided a large number of adsorption sites. Shen et al. [309] fabricated Ag-loaded ZnO microspheres gas sensor through a precipitation method for TEA detection. The high response to 100 ppm TEA at 183.5 °C was 6043, which was 14.8-fold higher than the pristine ZnO sensor. The mechanism of the 3D Ag-loaded porous ZnO microspheres sensor was displayed in Fig. 20h. Ag NPs decoration could promote the absorption of the oxygen species because of the spillover effect for Ag NPs. Ag NPs providing many active negative oxygen ions spill on to ZnO microspheres, which trap electrons from conduction band of ZnO, widen the electron depletion layer thickness, and further increase the resistance of the sensor.

#### Ru-Decorated SMOs-Based Gas Sensors

**Fig. 21:**
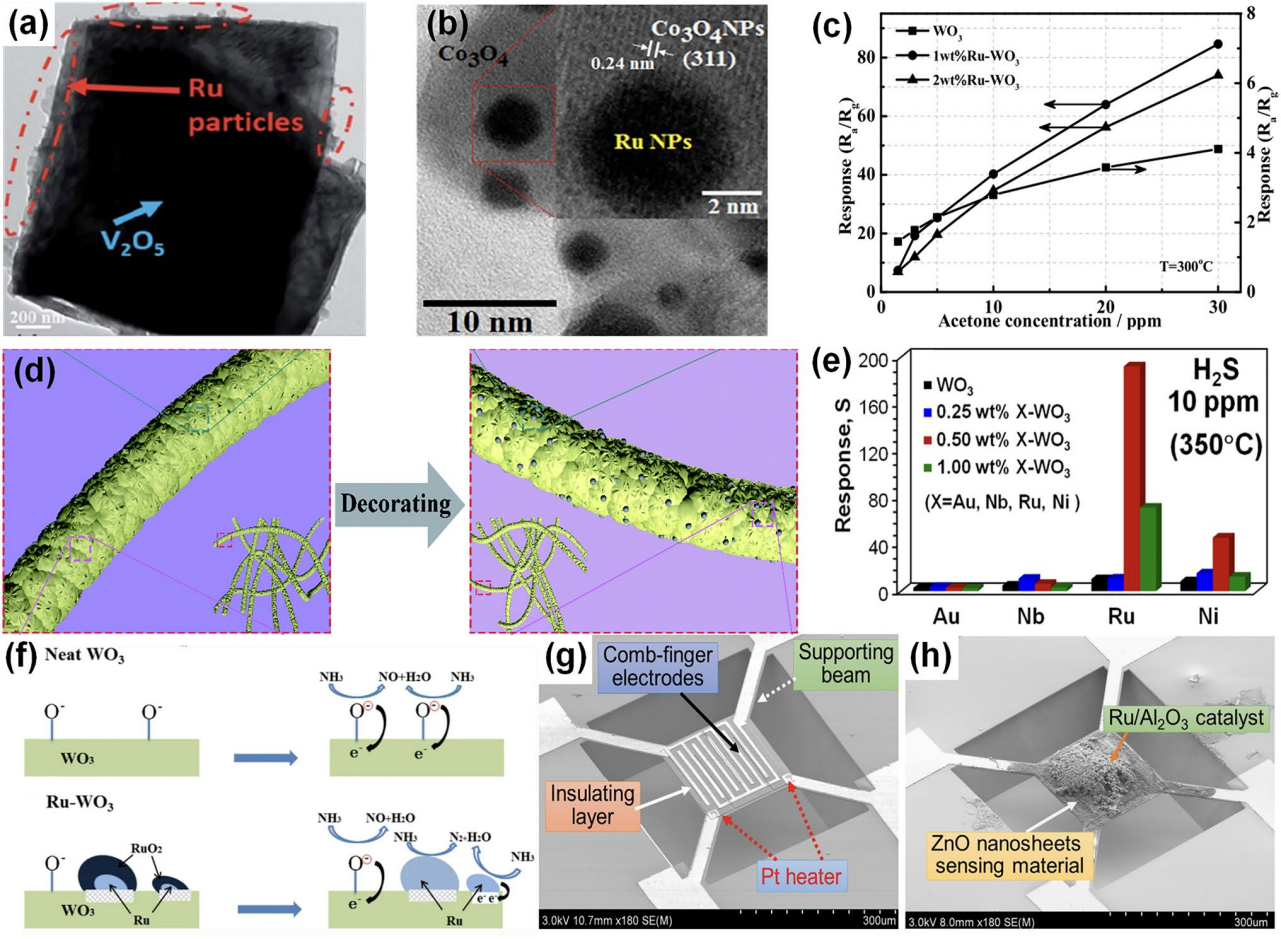
**a** Image of a typical particle in which V_2_O_5_ microparticles surrounded by Ru NPs. Reproduced with permission from Ref. [310]. Copyright 2019, Royal Society of Chemistry. **b** Image of the nanostructure decorated with Co_3_O_4_ NPs and Ru NPs. Reproduced with permission from Ref. [311]. Copyright 2018, Wiley–VCH. **c** Relationship between the response of Ru decorated WO_3_ nanoparticle sensor and acetone gas concentrations. Reproduced with permission from Ref. [312]. Copyright 2018, Elsevier. **d** 3D diagram of pure and Ru-decorated WO_3_ nanowires. Reproduced with permission from Ref. [313]. Copyright 2021, Royal Society of Chemistry. **e** Response of WO_3_ NRs decorated with different metals including Nb, Au, Ru and Ni. Reproduced with permission from Ref. [314]. Copyright 2015, Elsevier. **f** Mechanism of pristine WO_3_ and Ru-decorated WO_3_ sensors. Reproduced with permission from Ref. [316]. Copyright 2018, Springer Nature. **g-h** SEM images of the MEMS-based sensor with Ru-loaded-Al_2_O_3_/ZnO and unload sensing material. Reproduced with permission from Ref. [317]. Copyright 2018, Elsevier

Ru, as a catalyst metal, has been intentionally introduced for certain gas sensing, which improves the sensing behaviors especially the selectivity as well as time factors. Birajdar et al. [310] synthesized layer-structured V_2_O_5_ microparticles through a hydrothermal strategy, followed by a facile wet chemical route to modify Ru NPs. Figure 21a showed the morphology of a typical V_2_O_5_ particle decorated by Ru NPs. The response of the as-prepared gas sensor was 4% towards 130 ppm NH_3_ at RT. Compared with other RT gas sensors, the as-prepared sensor has short response/recovery time of 0.52/9.39 s. The presence of the RuO_2_/Ru clusters makes the water molecules dissociate less, and the greater sensitivity is obtained. Kruefu et al. [311] proposed Ru-loaded p-type Co_3_O_4_ NPs gas sensor through the precipitation and impregnation methods. As shown in Fig. 21b, the Ru NPs were successfully loaded on the Co_3_O_4_ NPs. Comparing the Co_3_O_4_ NPs sensors decorated with different amount Ru NPs, the 0.25 wt%Ru decoration was regarded as the optimal amount, which exhibited the highest response of 40 towards 1000 ppm ethanol at 350 °C. The Ru NPs serve as the catalyst to promote the oxidation rate of the ethanol around Ru sites. Besides, the product of the reaction will spill over to the surface of the adjacent Co_3_O_4_, which results to the improvement of the gas sensitivity. Li et al. [312] proposed WO_3_ NPs based on lamellar structure via an acidification method, followed by a modified impregnation method to decorate the Ru NPs. As shown in Fig. 21c, the Ru-decorated WO_3_ NPs sensors still have relatively sensitive signals even toward the low acetone concentration of 0.5 ppm. For instance, the response of the 1 wt% Ru-decorated WO_3_ sensor towards 0.5 ppm acetone was 7.3, which was 5 times higher than the pristine WO_3_ sensor. It was revealed that Ru NPs served as the catalyst to promote the activity of surface lattice oxygens due to the sensitization effect, which reacted with acetone and decrease the resistance of the sensor.

In addition to the nanoparticle structures and powder compounds described above, the 1D nanostructure was extensively applied for the enhancement of gas sensing properties. Li et al. [313] reported Ru-decorated 1D WO_3_ nanowires gas sensor via an electrospinning method. As shown in Fig. 21d, the Ru NPs were decorated onto WO_3_ nanowires as expected. The WO_3_ nanowires decorated with 4% Ru NPs exhibited the high response of ~ 120 towards 100 ppm ethanol, which was 47 times higher than the pristine WO_3_ nanowires. Moreover, the as-prepared sensor could detect as low as 221 ppb ethanol. Beyond that, the Ru-decorated WO_3_ nanowires could also increase O_C_, manifesting that more chemisorbed oxygen took part in the surface reaction. The activation energy of the target gas was reduced by the catalytic ability of RuO_2_, which could react with the adsorbed oxygen. Kruefu et al. [314] synthesized the Ru-functionalized WO_3_ nanorods via the hydrothermal and impregnation methods. The ultra-high response of the Ru-decorated WO_3_ nanorods gas sensor was ~ 192 towards 10 ppm H_2_S at 350 °C, with short response time of ~ 0.8 s. In addition, Ru-decorated WO_3_ nanorods were more selective for H_2_S, compared with the pristine WO_3_ nanorods. As shown in Fig. 21e, the Ru NPs decoration has higher catalyst selectivity towards H_2_S than other metals such as Ni, Nb, and Au. The Ru NPs could adsorb more oxygen species and H_2_S molecules, which would spill over to react with oxygen species on WO_3_ nanorods.

Moreover, 2D nanomaterials such as nanosheets decorated with Ru NPs were also currently employed to the field of gas sensing. Wang et al. [315] fabricated the Ru-decorated WO_3_ nanosheets gas sensor through a hydrothermal method. In compared with the pristine WO_3_ nanosheets sensor, the response of the 0.5 wt% Ru-decorated WO_3_ nanosheets sensor towards 100 ppm xylene was increased from 11 to 73, and the working temperature was reduced from 375 to 280 °C. Moreover, the as-prepared gas sensor was still sensitive towards the ultra-low concentration of 25 ppb. The outcome revealed that the sensor loaded with Ru NPs had better gas sensing performance than that loaded with Au, Pt, and Pd NPs. The catalytic property of the Ru NPs and the heterojunction between RuO_2_ and WO_3_ jointly determined the improvement of gas sensitivity. Thus, Ru NPs decoration was a valid strategy to promote the sensing behavior of the SMOs-based sensor. Qiu et al. [316] synthesized WO_3_ nanosheets through an acidification method, followed by a conventional impregnation process to decorate the Ru catalyst. The response of the Ru-decorated WO_3_ nanosheets gas sensor was ~ 18 towards 20 ppm NH_3_ at 300 °C, which was approximately twice that of the pure WO_3_ nanosheets. The Ru NPs decoration could promote the surface reaction due to the catalytic activity of Ru NPs. As shown in Fig. 21f, the catalytic effect of Ru NPs could make the NH_3_ more likely oxidize into N_2_, which inhibited the adsorption of NO_2_ molecule. Moreover, RuO_2_ could interact with the surface of WO_3_, and then form an additional depletion layer, which contributed to the sensitization effect. Currently, the construction of heterojunctions has been widely applied to improve the gas sensing properties because the interface states and the different energy band structures will change the carrier concentration. Liu et al. [317] synthesized a Ru/Al_2_O_3_/ZnO sensor through the inkjet printing technology. In this case, the Al_2_O_3_ loaded with Ru NPs served as the catalyst, which was deposited onto the surface of ZnO nanosheets. The response of the Ru/Al_2_O_3_/ZnO nanosheets gas sensor was ~ 65 towards 100 ppm SO_2_ at 350 °C. The as-prepared Ru/Al_2_O_3_/ZnO nanosheets were loaded onto the MEMS device, whose SEM image was exhibited in Fig. 21g–h. With the MEMS device, the suspended structure could improve the heat diffusion and maintain a consistent temperature with low power consumption.

#### Rh-Decorated SMOs-Based Gas Sensors

Rh, as a catalytically active transition metal, will form the metal–semiconductor contact between Rh NPs and nanomaterials, which can change the carrier concentration as well as oxygen vacancy and increase the chemisorbed oxygen. Rh NPs could promote the gas sensing behavior because of its strong electron mobility and great catalytic property for gas interaction. Unlike other noble metals like Pt, Pd, Au, and Ag, Rh NPs is a catalytically active transition metal of V period [318]. In addition, the size of NPs will affect the gas sensing behavior. However, Rh displays different gas sensing behavior compared to other noble metals. To be specific, the equilibration of the reaction with oxygen was promoted through larger Pt NPs, while Rh was opposite [319]. To sum up, decorating Rh NPs was an efficient method to enhance the sensing behavior.

**Fig. 22:**
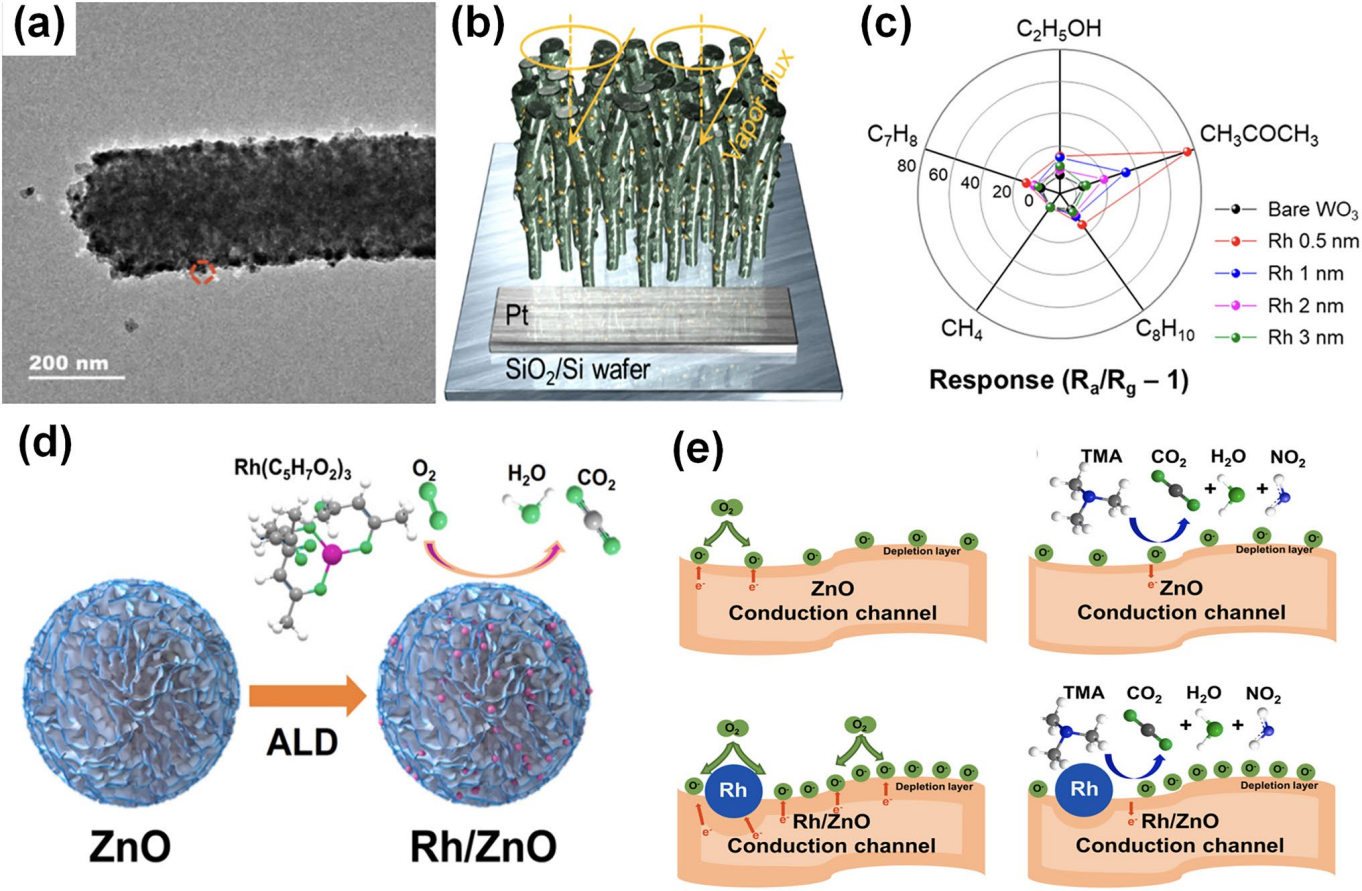
**a** TEM images of 5 mol% Rh-decorated NiO composite nanofibers. Reproduced with permission from Ref. [321]. Copyright 2021, Springer Nature. **b** The structure diagram of Rh-modified WO_3_ nanorods synthesized by the GLAD method. **c** The selectivity of the Rh-modified WO_3_ nanorods gas sensor towards various target gases (5 ppm) at 300 °C. Reproduced with permission from Ref. [44]. Copyright 2019, American Chemical Society. **d** Schematic synthesis of Rh-decorated ZnO nanoflowers through ALD method. **e** The sensing mechanism of Rh-decorated ZnO nanoflowers in air and in TMA. Reproduced with permission from Ref. [324]. Copyright 2022, Elsevier

Currently, the development of preparation technology lays a solid foundation for the development of metal modification. The sensitization mechanism is not only related to the type of noble metal, but also related to the loading methods [320]. How to decorate noble metals on the surface of nanomaterials efficiently and uniformly has become the focus of many scholars in recent years. Over the years, the electrospinning technique has been proved to be an economic and multifunctional method for mass production of 1D composite materials with high surface areas including nanofibers, nanotubes, and so on. Gao et al. [321] synthesized the Rh-decorated NiO nanofibers gas sensor through a facile electrospinning technique. The TEM image of 5 mol% Rh-NiO composite nanofibers exhibited in Fig. 22a demonstrated that the Rh NPs were decorated on the NiO nanofibers materials successfully. The outcomes revealed that the response of the Rh–decorated NiO nanofibers gas sensor working at 225 °C towards 200 ppm acetone was 70.10, which was 37.9-fold higher than the pristine NiO nanofibers sensor.

Besides, the GLAD technique has been extensively employed for the fabrication of Rh-decorated nanomaterials [322]. The morphology of Rh-decorated nanocolumnar structures was controlled through varying several deposition conditions [323]. Song et al. [44] fabricated a high sensing behavior acetone sensor based on the Ru-decorated WO_3_ nanorods gas sensor through the GLAD technique. When the glancing angle was set to 80°, the vertical-ordered Rh-decorated WO_3_ nanorods were synthesized successfully, as shown in Fig. 22b. Decorating Rh NPs could enhance the sensing performance through changing the morphological and producing the electronic sensitization as well as the chemical sensitization. As shown in Fig. 22c, the high response value of the as-prepared Rh-decorated WO_3_ nanorods was ~ 80 and exhibited strong selective detection towards acetone.

Nevertheless, achieving precisely controlled growth still remains difficult, and Rh NPs with the nanostructure tend to agglomerate, which affects their dispersity and utilization. Fortunately, the presence of the ALD technique could overcome the difficulty mentioned above. The ALD was a high controllable technique, which generated Rh-decorated nanomaterials at the atomic level with highly conformal and uniform. When using ALD technique to prepare Rh-decorated nanomaterials, the substrate was separately exposed to the precursor material, and an insert gas was used to purge between the precursor pulses. The unique property of depositing single monolayer makes it possible to control the deposition rate and form the film composition. Multi-layer nanocomposites could also be deposited controllably through changing different precursor materials. With these advantages, ALD became a powerful tool for depositing the Rh NPs onto the surface of the nanomaterials. Li et al. [324] reported a highly sensitive Rh-decorated ZnO flower-like nanostructures for TMA detection through the ALD technique. Figure 22d exhibited the synthesis of the Rh-decorated ZnO flower-like nanostructure. Specially, ALD technique has unique advantages to precisely control the Rh NPs deposited on the ZnO materials through changing the ALD growth cycles. The Rh/ZnO sensor with the 10 cycle Rh loaded by ALD showed that the best response to 10 ppm TMA at 180 °C was 11.3, which was threefold higher than that of pristine ZnO. The sensing mechanisms of ZnO and Rh/ZnO in air and in TMA were exhibited in Fig. 22e. The Rh NPs decoration could provide the oxygen molecules with more effective adsorption site, which increased the oxygen adsorption. The electron depletion layer will become thicker, resulting the increase of the resistance. In addition, the Rh NPs will decompose the TMA molecules into the high-activity radicals, facilitating the reaction between the TMA and the oxygen adsorption (Table 4).

**Table 4:** Summary of the reported other noble metal-decorated SMOs-based gas sensors

Materials	Structure	Synthesis method	Target gas	O. T. (°C)	Conc. (ppm)	Response	t_res_/t_rec_	LOD	Refs.
Ag/ZnO	Nanorods	Photochemical	Ethanol	280	50	34.8^a^	**–**	1 ppm	[325]
Ag/ZnO	Nanoparticles	Polymer-network gel	NO_2_	25	5	~ 4.2^a^	~ 150/180 s	0.5 ppm	[301]
Ag/ZnO	Microspheres	Photochemical	Ethanol	350	100	66.9^a^	< 7 s/–	500 ppb	[297]
Ag/ZnO	Nanosheets	Solvent reduction	Ethanol	300	0.1	~ 48%^b^	20 /25 s	1 ppb	[296]
Ag/ZnO	Microspheres	Precipitation	TEA	183.5	100	6043^a^	1 /60 s	1 ppm	[309]
Ag/ZnO	Nanocages	Calcination of MOFs	Ethanol	250	100	84.6^a^	510 s	23.1 ppb	[326]
Ag/ZnO	Nanoflowers	Hydrothermal	Ethanol	300	200	268^a^	2 /3 s	0.0178 ppm	[298]
Ag/ZnO	Nanocomposite	PLD	CO	25	40	~ 150%^b^	–	1 ppm	[303]
Ag/ZnO	Nanocomposite	Polymer-network gel	NO_2_	25	5	~ 3.4^a^	~ 75/160 s	1 ppm	[327]
Ag/ZnO	Nanoparticles	Polymer-network gel	NO_2_	25	5	~ 1.5^a^	~ 180/125 s	1 ppm	[302]
Ag/ZnO	Nanorods	Solvothermal	C_2_H_2_	250	100	255^a^	6/200 s	1 ppm	[304]
Ag/ZnO	Microspheres	Solvothermal	CO	130	100	~ 27^a^	2/25 s	44 ppb	[288]
Ag/ZnO	Nanoparticles	Gas-phase physical vapor deposition	Ethanol	250	100	~ 145^a^	–	10 ppb	[328]
Ag/SnO_2_	Nanowires	Electron-beam evaporation	Ethanol	450	25	228.1^a^	~ 0.4/80 s	5 ppm	[42]
Ag/SnO_2_	Nanocomposite	Wet-chemical	NO_2_	25	5	2.17^a^	49/339 s	1 ppm	[299]
Ag/SnO_2_	Nanosheets	Hydrothermal	TEA	170	100	1700^a^	6/15 s	10 ppm	[307]
Ag/SnO_2_	Nanowires	Sputtering	H_2_S	25	0.5	21.2^a^	18/980 s	0.25 ppm	[329]
Ag/WO_3_	Mesoporous	Impregnating	NO_2_	80	1	45^a^	303/147 s	100 ppb	[330]
Ag/WO_3_	Nanocomposite	Solution	NO	250	40	38.3^a^	–	–	[331]
Ag/WO_3_	Nanoplates	Photo-induced reducing	NO	170	5	~ 190^a^	~ 5/100 s	0.5 ppm	[332]
Ag/WO_3_	Nanosheets	Magnetic stirring	HCHO	300	100	20.83^a^	5/5 s	10 ppm	[333]
Ag/Fe_2_O_3_	Nanoparticles	Chemical coprecipitation	H_2_S	160	100	220^a^	42/26 s	50 ppm	[293]
Ag/In_2_O_3_	Nanoflowers	Chemical reduction	HCHO	200	20	~ 11^a^	0.9/14 s	5 ppm	[334]
Ag/ZnFe_2_O_4_	Microspheres	Hydrothermal	Acetone	175	100	33.4^a^	17/148 s	0.8 ppm	[308]
Ag/Bi_2_O_3_	Nanocomposite	Precipitation	Toluene	25	50	89.21^a^	~ 80/320 s	10 ppm	[335]
Ag/ZnCo_2_O_4_	Microspheres	Solution	Acetone	220	20	12^a^	4/20 s	0.25 ppm	[336]
Ag/CuO	Nanocomposite	Hydrothermal	NO_2_	~ 22	20	67.2%^b^	35/900 s	1 ppm	[289]
Ag/VO_2_	Nanorods	Stirring and ultrasonic dispersion	NO_2_	25	5	2.54^a^	104/306 s	2.54 ppm	[294]
Ag/ZnO/SnO_2_	Nanotubes	Electrospinning	HCHO	210	50	~ 100^a^	21/64 s	9 ppb	[337]
Ag/In_2_O_3_/ZnO	Nanocomposite	Nonaqueous	HCHO	300	1000	~ 300^a^	4/20 s	1 ppm	[300]
Ag/ZnO/In_2_O_3_	Nanofibers	Electrospinning and calcination	HCHO	260	100	186^a^	10/67 s	9 ppb	[305]
Ru/WO_3_	Nanowires	Electrospinning	Ethanol	200	100	~ 120^a^	23/58 s	221 ppb	[313]
Ru/Co_3_O_4_	Nanoparticles	Precipitation/impregnation/spin coating	Ethanol	350	100	~ 30^a^	0.08 s/–	10 ppm	[311]
Ru/WO_3_	Nanosheets	Hydrothermal	Xylene	375	100	73^a^	–	25 ppb	[315]
Ru/WO_3_	Nanosheets	Impregnation	H_2_	350	500	~ 100^a^	–	100 ppm	[338]
Ru/WO_3_	Nanoparticles	Acidification/impregnation	Acetone	300	0.5	6.4^a^	–	0.5 ppm	[312]
Ru/WO_3_	Nanosheets	Acidification/impregnation	NH_3_	300	5	~ 8^a^	–	3 ppm	[316]
Ru/WS_2_	Nanoflakes	Lithium intercalation	CO_2_	25	500	~ 17^a^	52/138 s	19 ppm	[339]
Ru/V_2_O_5_	Microparticles	Wet chemical	NH_3_	30	10	4^a^	1.5/9.3 s	10 ppm	[310]
Ru/WO_3_	Nanorods	Hydrothermal/impregnation	H_2_S	350	10	∼192^a^	~ 0.8 s/11 min	0.2 ppm	[314]
Rh/ZnO	Nanosheets	ALD	TMA	180	10	11.3^a^	63/106 s	55 ppb	[334]
Rh/NiO	Nanofibers	Electrospinning	Acetone	225	200	70.10^a^	134/222 s	0.25 ppm	[321]
Rh/MWCNTs	Nanotubes	Spray method	C_6_H_6_	25	0.5	~ 2^a^	–	50 ppb	[340]
Rh/WO_3_	Nanorods	GLAD	Acetone	300	5	~ 70^a^	11 s/–	0.2 ppm	[44]
Rh/MWCNTs	Nanotubes	CVD	Toluene	25	6	~ 7^a^	17/11 min	0.4 ppm	[341]
Rh/SnO_2_	Thin films	Spray pyrolysis	Ozone	250	~ 1	~ 20^a^	–	–	[318]

*O. T.* operating temperature; *Conc*. concentration; *t*_*res*_*/t*_*rec*_ response time/recovery time; *LOD* limit of detection

^a^Response is defined as R_a_/R_g_ or R_g_/R_a_, R_a_: resistance of the sensor in air, R_g_: resistance of the sensor exposed to target gas

^b^Response is defined as ∆R/R_a_ $\times$ 100% or ∆R/R_g_ $\times$ 100%, ∆R: the change in resistance, which equals to |R_a_–R_g_|

## Bimetal-Decorated SMOs-Based Gas Sensors

As discussed previously, the single noble metal has great enhancement to improve the gas sensitive performance like providing more active sites, promoting the dissociation of the gas molecules, and increasing chemisorbed oxygen. In comparison, bimetallic nanocrystals can not only combine two monometallic properties but also promote the physical and chemical characteristics owing to the synergistic effect between noble metals, which exhibits greater catalytic performance than the single metal decoration. Besides, the electronic structure and geometric configuration on the surface of bimetal particles can be changed by controlling the composition of the alloy [342]. Therefore, decorating bimetals was regarded as an effective strategy to further enhance the gas sensing performance.

The different nanostructure kinds of the bimetallic nanocatalysts were crucial to enhance the gas sensing performance. On the basis of the atomic ordering arrangement and construction of both two noble metals, diverse bimetallic nanostructures can be divided into three main types: alloy structures, core–shell structures, and heterostructures.

### Alloy Structure Bimetal Decoration

**Fig. 23:**
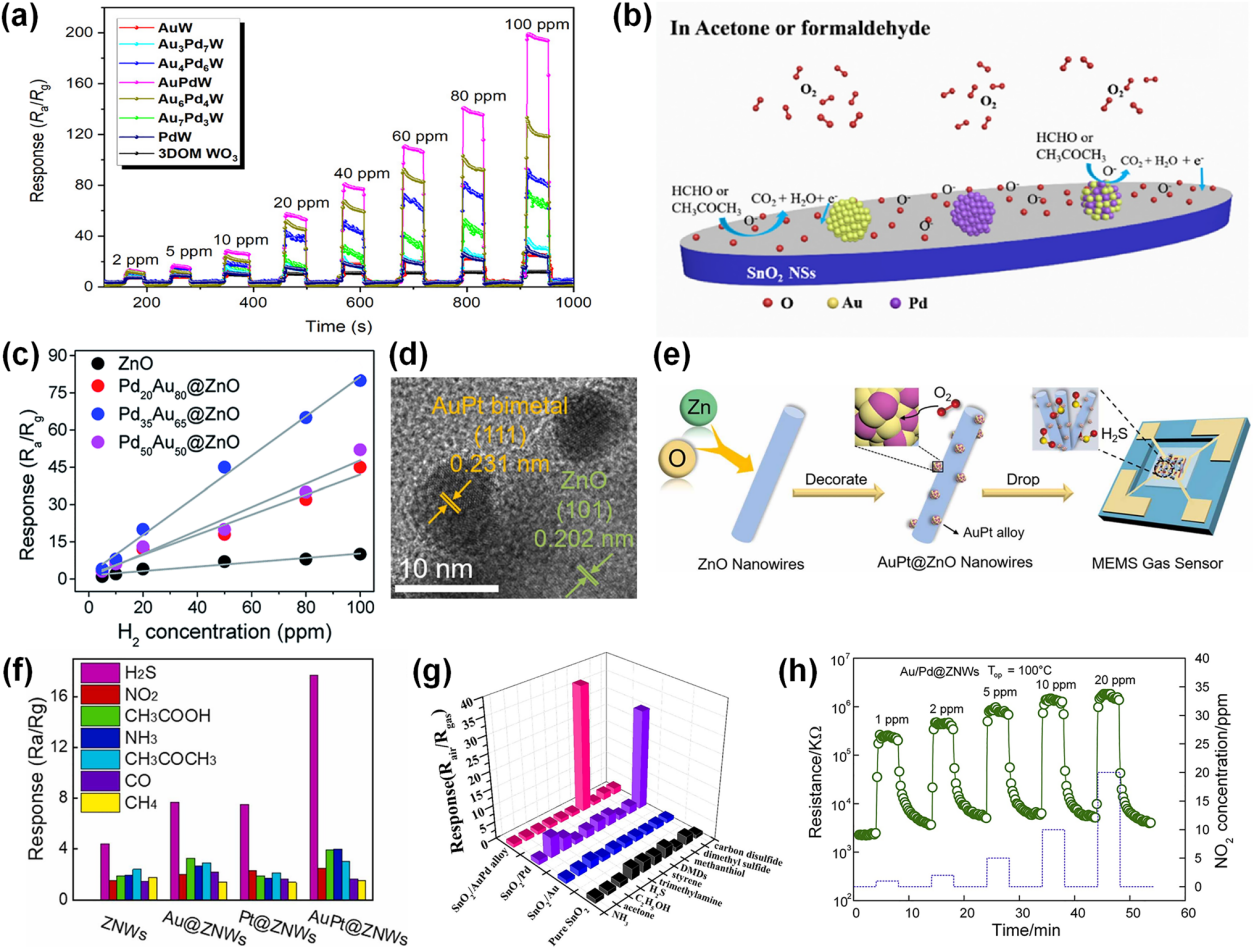
**a** Dynamic response curves of Au*y*Pd*z*W, AuW, PdW, and 3DOM WO_3_ sensors to 2 ~ 100 ppm acetone at 300 °C. Reproduced with permission from Ref. [343]. Copyright 2021, American Chemical Society. **b** Sensing mechanism diagram of Pd, Au, PdAu NPs decorated SnO_2_ NSs in acetone or formaldehyde ambience. Reproduced with permission from Ref. [344]. Copyright 2019, Elsevier. **c** Sensing response of ZnO, Pd_20_Au_80_@ZnO, Pd_35_Au_65_@ZnO, and Pd_50_Au_50_@ZnO in the range of 5–100 ppm H_2_. Reproduced with permission from Ref. [62]. Copyright 2020, Royal Society of Chemistry. **d** HRTEM image of the AuPt-decorated ZnO nanowires. **e** The fabrication of the AuPt-decorated ZnO nanowires. **f** Gas-sensing properties of the AuPt-decorated ZnO nanowires sensor. Reproduced with permission from Ref. [64]. Copyright 2022, Elsevier. **g** Selectivity comparison for pure SnO_2_, Au/SnO_2_, Pd/SnO_2_, and AuPd/SnO_2_ sensors toward 10 different interfering gases under a concentration of 10 ppm at 135 °C. Reproduced with permission from Ref. [47]. Copyright 2021, Elsevier. **h** Two reproductive cycles of Au/Pd@ZNWs were exposed to NO_2_ at 100 °C. Reproduced with permission from Ref. [59]. Copyright 2019, Elsevier

Typically, the alloy structure which contains inter-metallic and solid solution is most widely used in chemiresistive gas sensor. Zheng et al. [343] prepared AuPd/WO_3_ materials with different content of bimetallic AuPd alloy NPs on the surface of three-dimensional ordered macroporous (3DOM) WO_3_ and systematically investigated the effect of Au/Pd molar ratio on gas sensing properties. As shown in Fig. 23a, on the one hand, bimetallic AuPd alloy NP-decorated 3DOM WO_3_ exhibited higher acetone responses than monometallic-decorated one including AuW and PdW sensors. Meanwhile, the highest acetone responses were observed from the AuPdW sensor with the Au/Pd molar ratio of 1:1, revealing the optimum alloy ratio. In result, the AuPdW sensor with best Au/Pd molar ratio presented high response to trace acetone with fast response/recovery behaviors. Meanwhile, the AuPdW sensor had great selectivity and reproducibility. More importantly, the responses of the sensor towards acetone exhibited no obvious decline over 8 weeks, indicating a good long-term stability. Moreover, Li et al. [344] realized the decoration of Pd, Au and PdAu NPs on SnO_2_ nanosheets by *in-situ* reduction. The as-synthesized PdAu/SnO_2_ sensor displayed excellent gas performance, which can effectively detect acetone and HCHO as low as 45 and 30 ppb. The possible gas sensing mechanisms of SnO_2_ nanosheets decorated with PdAu bimetallic NPs were studied in detail, whose schematic diagram is shown in Fig. 23b. Compared with decorated with single Pd or Au, the improved response of PdAu NPs decorated SnO_2_ nanosheets may be attributed to the synergistic effect of PdAu bimetallic NPs. The PdAu/SnO_2_ sensor can detect low concentration of acetone and has good anti-interference to biomarkers and humidity in human breathing gas. Therefore, the prepared sensor has an appealing application in HCHO and breath acetone detection. Beyond that, the ZnO-based bimetal decorated SMO materials has made great contribution to the field of gas sensing The PdAu@ZnO core–shell nanoparticles (CSNPs) were successfully synthesized by Le et al. [62]. In the preparation process of hydrothermal method, the chemical composition of bimetallic PdAu nucleus was optimized by changing the reaction time to achieve the sensing purpose. The sensing response of pristine ZnO and PdAualloy@ZnO CSNPs in the range of 5–100 ppm H_2_ was shown in Fig. 23c. The Pd_35_Au_65_@ZnO sensor exhibited superior response of 80 to 100 ppm H_2_ at 300 °C. Furthermore, the obtained sensor had outstanding selectivity towards H_2_ and faster response/recovery time, which can be ascribed to the synergistic effects between Pd, Au, and ZnO. This work provides a promising design for sensitive and quick sensors to detect H_2_ leakage. Currently, the MEMS device was extensively employed to provide gas sensors with stable temperature condition and low power consumption. Liu et al. [64] fabricated the AuPt-decorated ZnO nanowires gas sensor based on MEMS devices for the H_2_S detection. As shown in Fig. 23d, the fringe spacing of the AuPt NPs were observed from the high-resolution TEM (HRTEM) images, which indicated that the Au and Pt NPs dissolved into each other and the metal atoms randomly mixed thoroughly. The fabrication of the MEMS-based AuPt-decorated ZnO nanowires gas sensor was exhibited in Fig. 23e. The AuPt-decorated ZnO nanowires were dropped onto the MEMS devices which could provide the heating condition for the gas sensor with low power consumption. As shown in Fig. 23f, the MEMS-based AuPt-decorated ZnO nanowires sensor exhibits the response value of 17.7 towards 20 ppm H_2_S at 300 °C, which is 4.0 times higher than the pristine ZnO sensor. Therefore, decorating AuPt NPs could enhance the sensitivity and selectivity of the sensors due to the electronic and chemical sensitization and the synergistic effect of the AuPt bimetal.

### Core–Shell Structure Bimetal Decoration

In addition to the alloy structure, the core–shell nanostructure of bimetallic nanocrystals has recently attracted the attention of many researchers due to a potential barrier generated at the interface, promoting the chemisorbed oxygen. The core–shell nanostructure is regarded as an ordered assembly structure, which is made up of a metal shell coated onto the other metal core through chemical bonds or other forces. Zhang et al. [345] employed the bimetallic Pd@Au core–shell nanoparticles (BNPs) to decorate W_18_O_49_ nanowires. The W_18_O_49_ nanowires/BNPs sensor had dual selectivity at different temperatures, which showed the response of 7.8 to 1000 ppm CH_4_ at 320 °C and response of 55.5 to 50 ppm H_2_S at 100 °C, respectively. This study is significant for detecting coal mine gases and appealingly applied to ensure coal mine safety production. Moreover, Hassan et al. [346] synthesized Pt/Pd bimetallic core–shell NP-decorated ZnO nanorod clusters (Pt/Pd-ZnO NRs) through hydrothermal method and PLD process. The Pt/Pd-ZnO NRs clusters displayed excellent selectivity among various gases. At the same time, the as-prepared sensor displayed a response of 58% and quick response of 5 s to 10,000 ppm H_2_ at 100 °C. In addition, Hassan et al. [347] also synthesized Pt/Pd bimetal decorated-vertical ZnO nanorods on the polyimide/polyethylene terephthalate (PI/PET) substrate for a flexible H_2_ sensor. The dynamic resistance changes of Pt/Pd-ZnO NRs with different bending angles was tested. It was found that the deviation of the response values was less than 4% not obvious after the bending to 90 degrees. The Pt/Pd-ZnO NRs with light weight and stable mechanical properties are expected to become an important component of flexible H_2_ sensors with fast response and great stability. Moreover, Liu et al. [47] demonstrated the modulation of both reaction kinetic process and electronic structure of the sensing surfaces through introducing bimetal alloy catalysts. The sensor of AuPd alloys modified SnO_2_ showed great gas response of 36.6 and significant selectivity towards dimethyl disulfide (DMDS) in Fig. 23g. The DFT calculations were used to confirm the surface adsorption enhancement of DMDS and charge transfer from AuPd alloy surface to SnO_2_, which may be the main reason for DMDS gas sensing response enhancement. Li et al. [60] used self-assemblies to obtain the PdPt NPs-decorated SnO_2_ nanosheets sensor, which showed temperature-dependent dual selectivity towards CO and CH_4_. The sensor also had great long-term stability and anti-humidity property. The sensing mechanism based on the diffusion depth of the measured gas in the PdPt/SnO_2_ nanosheets sensing layer was discussed. The enhancement of sensing response can be attributed to the electron sensitization of PdO, the catalytic activity of PdPt bimetal, and the Schottky barrier between SnO_2_ and PdPt NPs. The prepared sensor shows great application potential in coal mine gas monitoring. Specially, the bimetal hollow nanoframe was proposed to further enhance the gas sensing properties, which could increase the specific surface area, provide more active sites, and form the heterojunction between both two metals. Luo et al. [45] reported a Pd-Rh-decorated ZnO hollow nanocube (HC) with Rh-rich hollow frame and Pd-rich core frame using chemical method to etch the Pd-Rh bimetal solid nanocube (SC) in HNO_3_ aqueous solution. The unique Pd-Rh-decorated ZnO HC nanostructure could promote the electron transport during the sensing process. The enhanced H_2_S sensing properties could be ascribed to the excellent conductivity of Rh-rich frame increasing the specific surface area and gas diffusion, the remarkable catalytic ability of Pd-Rh bimetal, and the formation of Schottky barrier-type junctions and defect. Specially, PdRh-decorated ZnO HC sensor exhibited better response value of 185 towards 1 ppm H_2_S at 260 °C, which was 1.7-fold higher than the PdRh-decorated ZnO SC sensor.

### Heterostructure Bimetal Decoration

In addition to several structures described above, heterostructure is also a widely used structure. Two kinds of metals were deposited on the surface of the material, which formed the heterojunction at the interface of two metals and enhanced the gas sensing properties. Feng et al. [348] synthesized the Ag-Pt-decorated WO_3_ NPs through the hydrolysis and hydrothermal strategies. The as-prepared sensors exhibited the response of 250 (*R*_a_/*R*_g_) towards 100 ppm acetone at 140 °C, which was 6.1-fold compared to the pristine WO_3_ NPs under the same test condition. The enhanced gas sensing properties were owing to the chemical and electronic sensitization of Ag and Pt NPs to WO_3_ NPs, which increased the adsorption of oxygen, accelerated the reaction speed, and further promoted the gas sensing response. According to Choi et al. [46], the networked SnO_2_ nanowires were modified by bimetallic Pd/Pt NPs via the sequential γ-ray radiolysis method. The as-prepared sensor exhibited significant sensitivity of 80 and great selectivity towards 0.1 ppm NO_2_. Comparing with Pt-ZnO and Pd-ZnO nanowires, the bimetallic Pd/Pt NPs functionalized SnO_2_ nanowires sensor realized shorter response/recovery (13/9 s) times, which is beneficial from the synergic effect of Pd and Pt NPs. According to Chen et al. [59], the ZnO nanowires with Au/Pd bimetallic NPs decoration (Au/Pd@ZNWs) were synthesized for NO_2_ detecting. As shown in Fig. 23h, two reproductive cycles of Au/Pd@ZNWs were exposed to NO_2_ at 100 °C. Due to Au/Pd NPs decoration, the optimal working temperature of Au/Pd@ZNWs was reduced from 150 to 100 °C. At the same time, the Au/Pd@ZNWs exhibited higher gas response of 94.2 and shorter response/recovery time to NO_2_ than the Au@ZNWs and Pd@ZNWs. This study provided a feasible way for preparing bimetallic NPs modified 1D metal-oxide nanostructures for low-consumption and high-performance gas sensor application. Furthermore, Liu et al. [349] applied hydrothermal and *in-situ* reduction to fabricate the Ag/Pd@In_2_O_3_-based sensor. The decoration of Ag/Pd NPs improved the gas sensing performance with excellent sensitivity, quick response/recovery speed, and low detection limit of 20 ppb to toluene. Additionally, excellent selectivity and reproducibility were acquired. The sensor develops a low-cost, and more competitive approach to the detection of toluene gas, with significant benefits for healthcare and public safety monitoring (Table 5).

**Table 5:** Summary of the reported bimetal-decorated SMOs-based gas sensors

Materials	Structure	Synthesis method	Target gas	O. T. (°C)	Conc. (ppm)	Response	t_res_/t_rec_	LOD	Refs.
PtAu/ZnO	Nanorods	Hydrothermal	H_2_	130	250	157.4^a^	–	–	[58]
AuAg/MWCNTs/WO_3_	Composite films	Hydrothermal	NO_2_	25	1	~ 1700%^b^	267 s/–	45 ppb	[350]
AgPt/WO_3_	Nanoparticles	Hydrolysis and hydrothermal	Acetone	140	100	250^a^	41/13 s	–	[348]
AuPt/ZnO	Nanowires	Hydrothermal	H_2_S	300	20	17.7^a^	17/151 s	–	[64]
AgPt/TiO_2_/CuO/Cu_2_O	Thin films	Sputtering and hydrothermal	N-butanol	300	100	~ 200%^b^	12.4/4.2 s	–	[351]
PdRh/ZnO	Nanochips	Hydrothermal and stirring	H_2_S	260	1	185^a^	28 s/–	15 ppb	[45]
AuPd/SnO_2_	Nanosheets	*In-situ* reduction	Acetone HCHO	250 110	2 100	6.5^a^ 125^a^	5/4 s 68/32 s	45 ppb 30 ppb	[344]
AuPd/SnO_2_	Hollow spheres	*In-situ* reduction	DMDS	135	10	36.6^a^	23.5/26.5 s	5 ppm	[47]
AuPd/WO_3_	Nanorods	*In-situ* reduction and UV irradiation	Acetone	1000	200	4.53^a^	98/112 s	–	[352]
AuPd/WO_3_	Macroporous structure	Solution reduction	Acetone	300	10	12^a^	8/5 s	0.1 ppm	[343]
AuPd/ZnO	Nanowires	Dipping & heating evaporation	NO_2_	100	1	94.2^a^	35/30 s	1 ppm	[59]
PdAu/ZnO	Nanoparticles	Hydrothermal	H_2_	300	100	80^a^	0.6/12 min	5 ppm	[62]
PdAu/W_18_O_49_	Nanowires	Solvothermal	H_2_S CH_4_	100 320	50 1000	55.5^a^ 7.8^a^	10.2/8.5 s 24.8/14.5 s	–	[345]
PtPd/ZnO	Nanorod clusters	PLD	H_2_	100	10,000	58%^b^	5 s/–	0.2 ppm	[346]
PtPd/ZnO	Nanorods	PLD	H_2_	100	10,000	69.8^a^	5 s/–	0.2 ppm	[347]
PdPt/SnO_2_	Nanosheets	Hydrothermal	CO CH_4_	100 320	50 50	30^a^ 5.2^a^	30/78 s 5 s/–	–	[60]
PdPt/SnO_2_	Nanowires	γ-ray radiolysis	NO_2_	300	0.1	880^a^	13/9 s	0.1 ppm	[46]
AgPd/In_2_O_3_	Microsphere	*In-situ* reduction	Toluene	180	1	15.9^a^	7/13 s	20 ppb	[349]

*O. T.* operating temperature; *Conc*. concentration; *t*_*res*_*/t*_*rec*_ response time/recovery time; *LOD* limit of detection

^a^Response is defined as R_a_/R_g_ or R_g_/R_a_, R_a_: resistance of the sensor in air, R_g_: resistance of the sensor exposed to target gas

^b^Response is defined as ∆R/R_a_ $\times$ 100% or ∆R/R_g_ $\times$ 100%, ∆R: the change in resistance, which equals to |R_a_–R_g_|

## Challenges and Future Perspectives

Although significant progress has been achieved in the development of noble metal-decorated SMOs-based gas sensors, there still remain many challenges in the exploration of crucial performance enhancement and thorough mechanism investigation. Herein, the related challenges and future prospects of noble metal-decorated SMOs-based gas sensors are summarized below:

1. The gas sensing performance varies greatly depending on the kinds of noble metals decorating on the SMOs, and bimetals-decorated SMOs have been demonstrated to exhibit much better performance than that of single noble metal decoration. However, previous studies were mostly focused on monometallic decorations, and fewer reports on bimetallic or even polymetallic decorations have been published. Due to optimized electronic structures and geometric configurations as well as special synergistic effects, the bimetallic and polymetallic decorations are expected to be a promising research direction for further enhancing the gas sensing performance in the future.
2. With the development of IoTs, chemiresistive gas sensors with small size, high integration, and low power consumption are urgently required for the increasing demands in smart cities, healthcare, and especially smart phones. For example, power consumptions of lower than 10 mW are universal demands for battery-powered smart phone applications. Accordingly, MEMS-type and FET-type gas sensors provide promising and feasible avenues. Furthermore, since noble metals exhibit high catalytic activities, exploring noble metal-decorated SMOs-based gas sensors operating at RT is another promising development trend. Besides, with the help of light irradiation, highly photocatalytic noble metal-decorated SMOs have achieved great gas sensing performance at RT. Therefore, configuration with low-power micro-LEDs for photosensitization of sensing materials is also a feasible sensitization method.
3. The long-term stability is one of the crucial indicators for reliable applications of all the gas sensors, especially for noble metal-decorated SMOs-based sensors. However, noble metal nanoparticles suffer from shortcomings like easily being oxidized in air and agglomeration at relatively high operating temperature, which are major issues for the long-term stability. Moreover, as for practical applications, the anti-humidity of the gas sensors also needs close attention since the humidity varies greatly in different environments like dry desert region, coastal region, and even human exhaled breath. Therefore, more efforts are expected to effectively improve the long-term stability and anti-humidity of noble metal-decorated SMOs-based sensors. For example, developing high performance RT gas sensors is considered as a feasible strategy. Besides, establishing the accurate relationship between environment humidity and gas sensing performance by systematically recording different sensing behaviors under different environment humidity and then introducing applicable neural network algorithms to realize effective humidity compensation is a promising approach to minimize the humidity interference.
4. As is well known, related interfering gases existing in the actual environment will definitely affect the sensing performance of single gas sensors with poor selectivity. Thus, satisfactory selectivity is of vital importance for the practical application of noble metal-decorated SMOs-based gas sensos. Fortunately, the well-designed noble metal-decorated SMOs-based gas sensor arrays combined with machine learning algorithms for data processing have been broadly proposed, which demonstrate excellent abilities to recognize complex gas mixtures and even identify the concentrations of different gas components. Accordingly, it is possible to substantially improve the selectivity of noble metal-decorated SMOs-based gas sensors in combination with excellent neural network algorithms, which is unimaginable for traditional single sensors. However, at present, the mostly used algorithms for gas sensors adopt simple models, such as PCA and support vector machines, limiting the more effective utilization of sensing data. Therefore, more sophisticated algorithms like convolutional neural networks are extensively studied recently for further utilization of sensing data and substantial enhancement of sensing performance of gas sensor arrays. The integrated configuration of gas sensor arrays is a promising future trend for constructing functional electronic noses with satisfactory selectivity.
5. This review summarizes the related sensing mechanisms that have been proposed for noble metal-decorated SMOs-based gas sensors. However, different noble metals have their unique sensing mechanisms due to their specific chemical-physical properties. For instance, Pd will react with O_2_ to uniquely produce weakly bonded PdO, which could be easily dissociated and reproduce O_2_, thus improving sensing responses and accelerating response/recovery speed. In addition, the specific mechanisms of bimetallic and polymetallic decorations like special synergistic effects also remain unclear and need further exploration Recently, scientists have tried to conduct diverse *in-situ* characterizations including *in-situ* TEM and synchrotron radiation to explore the deeper sensing mechanisms of noble metal decorations. Nevertheless, more comprehensive and in-depth investigation is still needed in favor of explaining some special sensing behaviors and phenomena.
6. The repeatability on the mass production is one of the crucial issues between laboratory research and industry production, which will affect the stability of the sensors as well. Among various fabrication processes, screen printing, inkjet printing, and *in-situ* fabrication methods are promising for mass production with satisfactory repeatability. However, screen printing and inkjet printing are typical physical coating processes at first, which may cause worse stability of the sensors owing to the intrinsic limitation of poor contact. Meanwhile, the consistency between devices is also limited by the uniformity of slurries and inks, which remains a difficult technical issue due to agglomeration. Therefore, various *in-situ* fabrication processes like ALD, hard-template methods, and *in-situ* solution reaction methods, have been widely applied to synthesize advanced noble metal-decorated SMOs *in-situ* on MEMS or other gas sensor substrates, attracting extensive attention with increasing demand. The satisfactory consistency and great contact contribute to the excellent repeatability of mass production and great long-term stability of sensors. Therefore, the *in-situ* fabrication processes are prospective strategies for future industry mass production of gas sensor chips or devices.

## Conclusions

In this review, the recent progress of various noble metal-decorated SMOs-based chemiresistive gas sensors have been systematically summarized. Our attention has concentrated on the comparative review of different kinds of noble metals involving Au, Pt, Pd, Ag, Rh, Ru, and bimetals. Specifically, the preparation processes, gas sensing properties, and practical applications of various SMO materials, including n-type SMOs (ZnO, SnO_2_, WO_3_, In_2_O_3_, Fe_2_O_3_, TiO_2_, MoO_3_, CdO, CeO_2_, V_2_O_5_, etc.), p-type SMOs (CuO, NiO, Co_3_O_4_, Ga_2_O_3_, etc.), and their heterostructures, with noble metal decoration are comprehensively covered in this review. Meanwhile, the general sensing mechanisms for the performance improvement caused by noble metal decoration, including the electronic sensitization effect and the chemical sensitization effect, are also paid specific attention and discussed in detail. Furthermore, challenges and perspectives toward highly sensitive and selective gas sensors with low power consumption and excellent long-term stability have been comprehensively analyzed and discussed. This review is of considerable reference value for the design, fabrication, and development of noble metal-decorated SMOs-based gas sensors.

In general, noble metal-decorated SMOs have the advantages of facile synthesis, improved response values, fast response/recovery, fabulous selectivity, and excellent stability. The key conclusions are summarized as follows:

1. Noble metal decoration could effectively enhance the gas sensing performance of SMOs-based gas sensors through changing the surface nanostructure and morphology of SMO materials, enhancing the surface lattice oxygen activity, generating more oxygen vacancies, and increasing the gas molecules adsorption capacity. On the one hand, noble metals with excellent catalytic properties help to reduce the activation energy of the reaction between the gas molecules and adsorbed oxygens, thus accelerating the dynamic equilibrium between oxygen adsorption and desorption. On the other hand, Schottky barriers are expected to be formed when some noble metals with high work functions are in close contact with SMOs, which will cause the change of electron distributions and energy band structures in SMOs, and then lead to the increase in the thickness of electron depletion layer. More broadly, the decoration of noble metals on SMOs is not limited to gas sensors. It could also be extended to other functional applications including catalysis, CO_2_ reduction, and so on.
2. The design and control of the morphology of noble metal-decorated SMOs is well established. The development of preparation techniques has provided the basis for the advancement of noble metal decoration. Hydrothermal, UV reduction, ALD, electrochemical deposition, electrospinning, precipitation, magnetic sputtering, flame spray pyrolysis, and other preparation methods with unique characteristics have been widely applied to synthesize various noble metal decorated SMOs.
3. Bimetallic decoration has exhibited much better gas sensing performance than single noble metal decoration due to the synergetic benefits. Besides, the electronic structure and geometric configuration of bimetallic nanoparticles can be modulated by controlling the composition proportion of the bimetals to achieve the best sensing performance.
4. Different noble metals are specific for detecting different certain gases. For instance, Pd-decorated SMOs have a particularly high selectivity to H_2_ due to its unique reversible product of PdH_x_, while Ag-decorated SMOs are more sensitive to ethanol and HCHO as reported. Furthermore, the introduction of well-designed sorption, size-selective, or catalytic filters upstream of the noble metal-decorated SMOs-based gas sensors has been demonstrated to significantly improve the selectivity, which greatly benefits various practical applications especially in exhaled breath analysis with high humidity.
5. Diverse effective methods like light irradiation and morphological innovations have been developed to improve the catalytic activation of noble metals, further enhancing the gas sensing performance of noble metal-decorated SMOs-based gas sensors, such as reducing the operating temperature even down to RT, accelerating the response/recovery speed, and lowering the detection limit even down to ppb-level.

Thanks to the introduction of emerging functional materials, novel nanostructures, as well as advanced fabrication process, high-performance noble metal-decorated nanomaterials-based gas sensors have been more easily achieved. For the further step, the development of materials and devices with excellent consistency and long-term stability become even more crucial for future practical applications. Furthermore, the realization of long-term stable noble metals-decorated SMOs-based gas sensor arrays, together with the arrival of the artificial-intelligence (AI) era, should allow real-life electronic olfactory sensing in the future.
